# The Pine Cone Optimization Algorithm (PCOA)

**DOI:** 10.3390/biomimetics9020091

**Published:** 2024-02-01

**Authors:** Mahdi Valikhan Anaraki, Saeed Farzin

**Affiliations:** Department of Water Engineering and Hydraulics Structures, Faculty of Civil Engineering, Semnan University, Semnan 35131-19111, Iran; mvalikhan@semnan.ac.ir

**Keywords:** optimization, nature-inspired, pine tree, pine cone, mathematical benchmark functions, engineering problems, swarm intelligence

## Abstract

The present study introduces a novel nature-inspired optimizer called the Pine Cone Optimization algorithm (PCOA) for solving science and engineering problems. PCOA is designed based on the different mechanisms of pine tree reproduction, including pollination and pine cone dispersal by gravity and animals. It employs new and powerful operators to simulate the mentioned mechanisms. The performance of PCOA is analyzed using classic benchmark functions, CEC017 and CEC2019 as mathematical problems and CEC2006 and CEC2011 as engineering design problems. In terms of accuracy, the results show the superiority of PCOA to well-known algorithms (PSO, DE, and WOA) and new algorithms (AVOA, RW_GWO, HHO, and GBO). The results of PCOA are competitive with state-of-the-art algorithms (LSHADE and EBOwithCMAR). In terms of convergence speed and time complexity, the results of PCOA are reasonable. According to the Friedman test, PCOA’s rank is 1.68 and 9.42 percent better than EBOwithCMAR (second-best algorithm) and LSHADE (third-best algorithm), respectively. The authors recommend PCOA for science, engineering, and industrial societies for solving complex optimization problems.

## 1. Introduction

**Figure 1:**
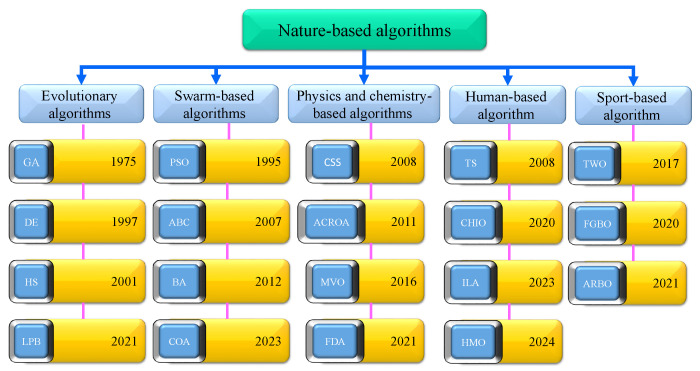
Nature-based optimization algorithms in various categories.

Nature-inspired optimization algorithms refer to the new optimization methods designed based on natural phenomena. These algorithms can solve optimization problems without the need to calculate the gradient of the objective function. Hence, nature-inspired optimization algorithms can solve non-derivative optimization problems. Nature-inspired optimization algorithms are flexible and can handle many constraints and decision variables. In contrast, the computation times of numeric optimization methods increase exponentially with the number of constraints and decision variables. Thanks to the design and development of nature-inspired algorithms, it is possible to solve complex problems that have not been solved so far. Although many nature-based algorithms have been proposed, new algorithms are still being developed due to the “no free lunch in search and optimization” theory [1]. According to this theory, each optimization algorithm can solve some optimization problems. Some problems still need to be solved, and new nature-inspired optimization algorithms can solve them with better accuracy. Researchers divide these algorithms into six categories based on the type of idea that inspires them: evolutionary, swarm-based, mathematical-based, physics and chemistry-based, sport-based, and human-based. Evolutionary algorithms are based on the principle of natural evolution. They begin by creating a random population, which then evolves through selection, crossover, and mutation operations. The most popular evolutionary algorithms are Genetic Algorithm (GA) (developed in 1975) [2]; Differential Evolution (DE) (developed in 1997) [3]; Harmony Search (HS) (developed in 2001) [4]; Black Widow Optimization Algorithm (BWO) (developed in 2020) [5]; Learner Performance Based Behavior algorithm (LPB) (developed in 2021) [6]; Evolutionary Mating Algorithm, based on the adoption of the random mating concept from the Hardy–Weinberg equilibrium and crossover index [7]; and One-Dimensional Subspaces Optimization Algorithm (1D-SOA) [8]. Although evolutionary algorithms have seen successful application in some problems, these algorithms slowly converge to the optimal solutions and become stuck in local optima. Swarm-inspired algorithms are another type of nature-inspired algorithms. These algorithms are designed based on the moving methods of swarms. The most popular swarm-based algorithm is the Particle Swarm Optimization algorithm (PSO) (developed in 1995) [9]. Moving swarms of birds or fish inspire the search processing in PSO. Researchers introduced the Artificial Bee Colony (ABC) in 2007 [10], Firefly Algorithm (FA) [11] in 2008, Bat Optimization Algorithm (BA) in 2012 [12], Krill-Herd Algorithm (KHA) in 2012 [13], Gray Wolf Optimizer (GWO) in 2014 [14], Whale Optimization Algorithm (WOA) in 2016 [15], Harris Hawks Optimization Algorithm [16] in 2019, Coati Optimization Algorithm (COA) in 2023 [17], Dung Beetle Optimizer (DBO) in 2023 [18], Kookaburra Optimization Algorithm (KOA) in 2023 [19], Giant Armadillo Optimization (GAO) in 2023 [20], Lyrebird Optimization Algorithm (LOA) in 2023 [21], and Humboldt Squid Optimization Algorithm (HSOA) in 2023 [22]. In this category, algorithms such as PSO have poor local optimization ability. The bat algorithm has yet to establish a good balance between global and local search ability. KH has two main limitations: stagnation in local optima and a slow convergence speed. GWO and WOA use adaptive weights to control the search scope and balance global and local search capabilities. However, the global search ability of these algorithms is weak. The other types of nature-inspired optimization algorithms are physics and chemistry-based algorithms. These algorithms are designed based on the laws of physics or chemistry. Some of the well-known algorithms in this category include Central Force Optimization (CFO) (developed in 2008) [23], Charged System Search (CSS) Optimization Algorithm (developed in 2010) [24], Artificial Chemical Reaction Optimization Algorithm (ACROA) (developed in 2011) [25], Multi-Verse Optimizer (MVO) (developed in 2016) [26], Flow Direction Algorithm (FDA) (developed in 2021) [27], and Rime Optimization Algorithm (RIME) (developed in 2023) [28]. Certain optimization problems may pose challenges for physics and chemistry-based algorithms. In particular, ACROA may suffer from slow convergence as a result of unbalanced local and global searches, while MVO may lack diversity, resulting in trapping and premature convergence. Human-based optimization algorithms are another category of nature-inspired optimization algorithms. These algorithms work based on human-based phenomena. In 1989, Tabu Search (TS) was developed, using the mechanics of human memory [29]. The other well-known algorithms in this category consist of the Poor and Rich Optimization (PRO) algorithm (developed in 2019) [30], Coronavirus Herd Immunity Optimizer (CHIO) (developed in 2019) [31], Incomprehensible but Intelligible-in-Time Logics: Theory and Optimization Algorithm (ILA) (developed in 2023) [32], Technical and Vocational Education and Training-Based Optimizer (TVETBO) (developed in 2023) [33], and Human Memory Optimization (HMO) (developed in 2024) [34]. The category of sport-based algorithms includes optimization algorithms that are motivated by sports methods. The best known of these algorithms are Tug of War Optimization (TWO) (developed in 2017) [35], Football Game Based Optimization (FGBO) (developed in 2020) [36], and Athletic Run Based Optimization (ARBO) (developed in 2021) [37]. Efforts to develop and create new optimization algorithms are still ongoing. In this regard, researchers developed the Polar Bear Optimization Algorithm [38]; a modified Whale Optimization Algorithm, based on different searching paths and perceptual disturbance [39]; an Improved Butterfly Optimization Algorithm, based on the cross-entropy method [40]; and an improved Chaotic Particle Swarm Optimization Algorithm, with a more symmetric distribution [41]. Figure 1 shows nature-inspired optimization algorithms in the different categories.

Although researchers have studied various natural phenomena to design nature-based algorithms, phenomena related to the life of plants, despite their high potential for designing nature-based algorithms, have received less attention. The studies in this field are reviewed in the following section. Researchers developed the Invasive Weed Optimization (IWO) algorithm in 2006, based on colonizing weeds [42]. IWO selects better fitness seeds and distributes them in a search space. It uses normal-distribution random numbers with a large standard deviation in the exploration phase and decreases the standard deviation in the exploitation phase. IWO uses adaptive standard deviation but does not share information between seeds. The Flower Pollination Algorithm was introduced in 2012. The pollination mechanism of flowers inspires this algorithm [43]. FPA uses Levy flight to balance exploration and exploitation. It updates the position of pollen based on the best position, the current position, and Levy distribution. When exploiting, it updates the position by randomly choosing two pollen grains and generating a random number. It does not use dispersing seeds and assumes an equal chance for exploration and exploitation. In 2015, the Tree Seed Algorithm (TSA) was motivated by the relationship between trees and their seeds for continuous optimization [44]. TSA uses the best tree and seed positions as selection operators. TSA generates multiple seeds around the current and a random tree during exploration. During exploitation, TSA generates numerous seeds around the tree based on the best position of all trees, a random tree, and the current position of the tree. TSA uses the wind impact to disperse seeds but does not involve animals, pollination, or Levy flight. Researchers in 2018 developed the Tree Growth Algorithm (TGA) based on the competition among trees for acquiring light and food [45]. TGA categorizes trees into four fitness groups and uses different methods for exploration and exploitation. It balances exploration and exploitation but does not use dispersing seed strategies, a pollination process, or Levy flight. In 2022, the Trees Social Relations Optimization Algorithm was inspired by the hierarchical and collective life of trees in the jungle [46]. This algorithm has advantages, such as dividing the search domain into sub-clusters. However, it does not incorporate pollination mechanisms or the roles of animals in seed dispersal. In 2023, researchers proposed the Orchard Algorithm (OA), based on the fruit gardening process [47]. This algorithm used a neighborhood search. However, it does not use clustering search space, pollination mechanisms, or dispersing seeds. The Tree Optimization Algorithm (TOA) was created by [48] in 2023. The growth of trees inspired this algorithm. However, TOA does not consider reproduction methods for one tree.

To the best of the authors’ knowledge, previous research on nature-inspired optimization algorithms has given less attention to the excellent life potential of plants, particularly their reproductive methods. While there have been some studies on developing nature-based algorithms, little work has been performed on exploring the different aspects of tree reproduction. As a result, this study introduces a new algorithm named the Pine Cone Optimization Algorithm (PCOA), which is based on the unique reproductive strategies of pine trees. These strategies have enabled the pine species to survive for millions of years, and simulating them in developing PCOA could offer solutions to scientific and engineering issues. The new contributions of this study are as follows:

- Considering different pollination mechanisms for simulating the impact of wind on pine tree pollination.
- Considering the impacts of animals and gravity on pine cone dispersal using new operators.
- Clustering the search domain to sub-cluster and search in each of them in parallel.
- Shrinking the search domain over course generations.
- Using both swarm-based and evolutionary operators in updating solutions to the PCOA.

PCOA is set apart from other existing algorithms due to these contributions. The remainder of the paper is structured as follows: Section 2 describes the pine tree’s reproduction methods, introduces the PCOA theory and its structure and flowchart, and discusses the mathematical and engineering problems. Section 3 presents the accuracy, convergence efficacy, and time complexity of PCOA in classic and new mathematical benchmark functions and engineering problems. Lastly, Section 4 presents the conclusions and future scope.

## 2. Materials and Methods

### 2.1. Inspiration: The Life Cycle of the Pine Tree

**Figure 2:**
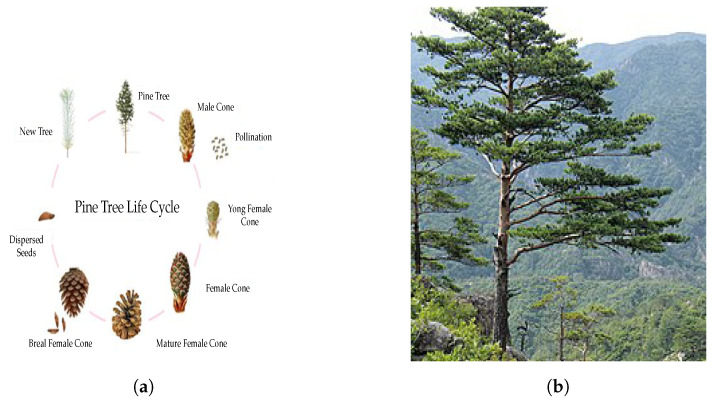
Pine tree life cycle and swarms in the forest. (**a**) Pine tree life cycle. (**b**) Pine tree forest.

Pines are coniferous trees in the Pinus family of the Pinaceae, which grow in a wide range of ecosystems. They grow to 50 m in height with dark green needles and 5–10 cm cones with rounded scales [49]. Some pines, like Scotch pine, are monoecious because they have female and male flowers on the same tree. Tree shoots have male flowers at their base. The wind pollinates female flowers in the early summer. The female flower develops into small cones that quickly grow and reach full size by early summer after more than a year. Then, gravity causes the cones to break away and fall to the ground, or animals eat them and disperse the seeds in spring. Eventually, the seeds turn into saplings, and new trees arise. Plants like the pine tree are pollinated only by the wind and, unlike other plants, insects do not affect their pollination [50,51]. Figure 2 shows the life cycle of pine trees and pine tree swarms in the forest.

### 2.2. Mathematical Modeling of Pine Reproduction

The pollination and dispersal of cone seeds is a critical phase of the life cycle of pine trees. Therefore, the present study focuses only on considering the pollination and dispersion of cones. In PCOA, gravity and animals scatter the cones, just like in nature. Continuing with the study, we mathematically express the PCOA, considering the pollination and scattering of cones through gravity and animals. According to previous studies, pine tree reproduction is significantly dependent on pollination [52], and dispersion of cone seeds by gravity [53] and animals [54]. Pollination in a pine tree is similar to solving an optimization problem in two aspects. In one aspect, the movement of pollen particles between cones of pine trees can be simulated by a swarm-based operator. In this case, pollen particles move driven by the wind. The atmosphere of the forest is the search space for problems, and the pollen particles are the search agents. Each particle has a position and objective function in the search space of the optimization problem. The movement of pollen particles from one pine tree cone to another is similar to searching for the optimal solution in the problem’s search space. In another aspect, pollen particles enter the female flower of the pine tree and cause the female flower to develop and create small cones. Therefore, this process can be simulated by evolutionary operators. The evolution of the pine cone from one state to another is similar to improving the problem solution and finding its optimal solution. The scattering of pine cone seeds by gravity and animals causes the creation and growth of pine seedlings in new situations. If the position of the seedling is suitable, it will become a pine tree. Similarly, in the PCOA, the answers are updated if a better position is found. The pine tree forest is equivalent to the problem search space, and the pine cone seeds are the search agents. Population-based operators can be used to simulate the dispersion of pine cone seeds by gravity or animals. Hence, population-based and evolutionary-based operators can simulate the mentioned biological principles and solve optimization problems. In this regard, the position of the pollen, seeds of cones, and pine trees are the search agents, and the best position for growing the pine tree is the global optimal problem.

### 2.3. Generating the Initial Population in PCOA

The PCOA has two populations: pine trees and their cones. The problem domain is divided into equal segments. In the algorithm initialization, there is a tree in the center of each segment, and multiple cones are generated around each tree. For this purpose, the boundary conditions for each part are given by

(1) $$LbS=lb+\left(i-1\right)\times \frac{ub-lb}{N_{tree}}$$

(2) $$UbS=ub+\left(i-1\right)\times \frac{ub-lb}{N_{tree}}$$

In Equations (1) and (2), lb and ub are the lower and upper bounds of the problem’s domain, and LbS and UbS are each segment’s lower and upper bounds. The pine cone population is generated as follows:

(3) $$CX_{j,i}=LbS_{i}+{\vec{rand}}_{1\times dim}\times \left(rand\times UbS_{i}-rand\times LbS_{i}\right)$$

In Equation (3), CX is the pine cone position, ${\vec{rand}}_{1\times dim}$ is a random vector with a normal distribution between 0 and 1, and rand is a random number with a normal distribution between 0 and 1. In operators of PCOA, *i* indicates the pine tree index and *j* shows the cone index. After initializing PCOA, the position of each tree is equal to the position of the best cone in that segment.

### 2.4. Pine Cone Dispersal by Gravity (Exploitation)

When the pine cones mature and their weight increases, gravity causes the pine cones to fall from the tree. The issue causes the cones to disperse in a limited area. The search space problem is divided into multiple super-cubes to simulate this process. Each of these super-cubes contains a tree inside. In order to accurately simulate the impact of gravitational forces on the dispersion of cones in the vicinity of trees, it is necessary to generate multiple solutions around each tree. In order to accomplish this objective, the following operator is utilized.

(4) $$CX_{j,i}^{new}=\left\{\begin{array}{cc} TX_{i}+w_{1}\times R_{1}\times \left(R_{2}\times \left(Ub_{s}^{i}-Lb_{s}^{i}-TX_{i}\right)\right), & if\;Control_{parameter}=0\\ CX_{j,i}+w_{1}\times R_{1}\times (R_{2}\times \left(Ub_{s}^{i}-Lb_{s}^{i}-Tpop_{all,r1}\right)-Tbest_{x,i}, & \mathrm{otherwise}. \end{array}\right.\;$$

In Equation (4), $CX_{j,i}^{new}$ is the new position of the pine cone, $CX_{j,i}$ is the updated position of the pine cone, $TX_{i}$ is the position of the tree, $Ub_{s}^{i}$ is the upper bound of the super-cube, $Lb_{s}^{i}$ is the lower bound of the super-cube, $Tpop_{all,r1}$ is a randomly selected solution in the memory of PCOA, $Tbest_{x,i}$ is the ith of the top solutions of PCOA, $w_{1}$ is an adaptive weight, r1 is a random integer number between 1 and the size of the PCOA memory, and $R_{1}$ and $R_{2}$ are normal random numbers between 0 and 1. In the operators of PCOA, i shows the pine tree index, and j indicates the cone index. The boundaries of the problem (lb and ub), LbS and UbS, shrink over the generations. In this approach, the research space is restricted to the closing area in the best surrounding position over the generations. This approach enables the PCOA to balance exploration and exploitation. To achieve this, we define the following equations:

(5) $$LbS=Lb+\left(i-1\right)\times \frac{Ub-Lb}{N_{tree}}$$

(6) $$UbS=Ub+\left(i-1\right)\times \frac{Ub-Lb}{N_{tree}}$$

(7) $$Lb=lb+Radius_{lb}\times W$$

(8) $$Ub=ub-Radius_{ub}\times W$$

(9) $$W=\min FES/FES_{max},0.5$$

(10) $$Radius_{lb}=X_{best}-lb$$

(11) $$Radius_{ub}=ub-X_{best}$$

In Equations (5)–(11), the algorithm updates the boundaries of the problem, Lb and Ub. It also determines the shrinking radius with $Radius_{lb}$ and $Radius_{ub}$. The adaptive weight *W* decreases from 1 to 0.5. $X_{best}$ represents the current best position. FES signifies the current generation, with $FES_{max}$ denoting the maximum number of generations.

### 2.5. Pollination (Exploration)

In pollinating pine trees, the agents are produced from male cones and drift to female cones. This movement can occur from one tree’s cones to those of another or within the same tree. As this movement is very complicated, it is possible to define different mechanisms to simulate it. In PCOA, two different methods are defined to mimic this motion. In the first method, Algorithm 1 is used to simulate wind pollination. In the second method, wind pollination is simulated according to the study by [55]. This method is formulated as follows:

(12) $$CX_{j,i}^{new}=CX_{j,i}+0.5\times \phi_{r_{1}}\times \left(Tbest_{j}-CX_{r_{1},i}\right)+0.5\times \phi_{r_{3}}\times \left(Tbest_{j}-CX_{r3,i}\right)$$

In Equation (12), ϕ is the chance of successful pollination of pine cones. ϕ is calculated as follows:

(13) $$\phi_{i}=1-e^{-\gamma *\sum_{j=1}^{n}a_{i,j}}$$

(14) $$a_{i,j}=\frac{\frac{\beta }{d_{i,j}^{\alpha }}-\frac{\alpha }{d_{i,j}^{\beta }}}{\beta -\alpha }$$

In Equations (13) and (14), $a_{i,j}$ is the effect of the ith cone on the jth cone, and $d_{i,j}$ is the Euclidian distance between the ith cone and jth cone. In addition, α, β, and γ are constant parameters that define the pollination process mathematically. According to the conducted study by [55], the values of α, β, and γ are set to 3, 40, and 0.62, respectively.
**Algorithm 1** Pollination**Input:** Pollination cycle ($P_{N}$), cones position (CX), cones fitness (CF)**Output:** Pine Cone’s position   *Wind’s pollination*:1:  **while**  *N* < $P_{N}$ **do**2:     N←N+13:     $Memory_{Index,rand}\leftarrow \left\lfloor dim*\vec{rnd}\right\rfloor$4:     $\mu_{W_{1}}=Memory_{W_{1}}\left(Memory_{Index,rand}\right)$5:     $\mu_{W_{2}}=Memory_{W_{2}}\left(Memory_{Index,rand}\right)$6:     $\mu_{W_{3}}=Memory_{W_{3}}\left(Memory_{Index,rand}\right)$7:     $\mu_{cr}=Memory_{cr}\left(Memory_{Index,rand}\right)$8:     $W_{1}\leftarrow \mu_{W_{1}}+\vec{Rnd_{1}}\times \tan\left(\pi \times \left(\vec{Rnd_{2}}-0.5\right)\right)$9:     $W_{2}\leftarrow \mu_{W_{2}}+\vec{Rnd_{3}}\times \tan\left(\pi \times \left(\vec{Rnd_{4}}-0.5\right)\right)$10:     $W_{3}\leftarrow \mu_{W_{3}}+0.1\times \tan\left(\pi \times \left(\vec{Rnd_{5}}-0.5\right)\right)$11:     $cr\leftarrow \mu_{cr}+0.1\times \vec{Rnd_{6}}$12:     **if** $W_{1}$ < 0 **then**13:      $W_{1}\leftarrow \mu_{W_{1}}+0.1\times \tan\left(\pi \times \left(\vec{Rnd_{7}}-0.5\right)\right)$14:     **end if**15:     **if** $W_{2}$ < 0 **then**16:      $W_{2}\leftarrow \mu_{W_{2}}+0.1\times \tan\left(\pi \times \left(\vec{Rnd_{8}}-0.5\right)\right)$17:     **end if**18:     **if** $W_{3}$ < 0 **then**19:      $W_{3}\leftarrow \mu_{W_{2}}+0.1\times \tan\left(\pi \times \left(\vec{Rnd_{9}}-0.5\right)\right)$20:     **end if**21:     $W_{1}\leftarrow \min\left(W_{1},1\right)$, $W_{2}\leftarrow \min\left(W_{2},1\right)$, $W_{3}\leftarrow \min\left(W_{3},1\right)$, cr←min(cr,1), cr←max(cr,0)22:     Generate three random integer number ($r_{1},r_{2},r_{3}$)23:     Choose $Tbest_{rate}\%$ of best solutions and save them on Tbest;24:     Choose $Tworst_{rate}\%$ of worst solutions and save them on Tworst;25:     **if** rand < 0.5 **then**26:      $CX^{new}\leftarrow XC+W_{1}\times \left(Tbest-CX_{r1}\right)+W_{2}\times \left(CX_{r1}-Tpop_{all,r_{2}}\right)$27:     **else**28:      W=max(W1×W2,(1−W1)×W2,1−W2)29:      $CX^{new}\leftarrow W2\times \left(W1\times CX+\left(1-W1\right)\times Tbest\right)+\left(1-W_{2}\right)\times CX_{r1}+W\times \left(CX_{r3}-Tpop_{all,r_{2}}\right))$30:     **end if**31:     Evaluatefitnessfunction32:     **if** $CF^{new}$ < CF || rand<cr **then**33:      $XC\leftarrow XC^{new}$, $XF\leftarrow XF^{new}$34:     **end if**35:     Update $Tpop_{all}$ (Add new solution to archive and remove duplicate or randomly remove some solutions to maintain the archive size);36:     Update $\mu_{W_{1}},\mu_{W_{2}},\mu_{cr}$37:  **end while**
38:  **return** Pine Cone’s position

### 2.6. Pine Cone Dispersal by Animals (Exploitation)

Animals eat pine cones and disperse them in the environment. For example, in the fall, squirrels bury pine cones to eat later on [56]. Birds and other animals like bears, deer, rats, and mice eat pine cones. In the PCOA, four operators are defined to simulate these behaviors. In the first operator, it is assumed that animals like the squirrel carry pine cones from a random position to a position with a better condition for burial. This operator employs the quadratic programming optimization method to find the best location. The initial point in this method is defined as follows:

(15) $$X_{initial}=X_{best}+rand\times \left(\bar{CX}-X_{best}\right)$$

In Equation (15), $X_{initial}$ is the initial position in quadratic programming, and $\bar{CX}$ is an average of all the pine cones’ positions. It should be noted that the quadratic programmatic search is performed under the updated boundary conditions of the problem. This approach allows the PCOA to approximate the global optimum better. The roles of animals in scattering pine cones are the basis for driving the second to fourth operators. The second operator is designed as follows:

(16) $$X_{animal}=\frac{X_{best}+X_{animal}}{2}+levy\times \left(levy\times \left(lb+ub-\frac{X_{best}+X_{animal}}{2}\right)-\frac{X_{best}+X_{animal}}{2}\right)$$

In Equation (16), $X_{animal}$ is known as the position of the pine cones carried by animals, a Levy random number generated by the Levy distribution. In the third operator, it is assumed that animals carry pine cones from their position to near a tree’s position. The position of the pine cone is updated by the third operator as follows:

(17) $$X_{animal}=CX+\left(1-w_{d}\right)\times \bar{TX}+w_{d}\times levy\times \left(levy\times \left(lb+ub-\bar{TX}\right)-\bar{Tree_{x}}\right)$$

In Equation (17), $\bar{TX}$ is the average position of the pine trees, $w_{d}$ is an adaptive weight that is given by [57]:

(18) $$w_{d}=exp-20\times \frac{FES}{FES_{max}}$$

$w_{d}$ limits the search domain over increasing function evaluations and leads to converting exploration into exploitation. The fourth operator assumes that an animal carries a pine cone in a nearer position:

(19) $$X_{animal}=CX+w_{d}\times levy\times \left(levy\times \left(lb+ub-CX\right)-CX\right)$$

In PCOA, exploitation falls under the responsibility of all four defined operators. Algorithm 2 illustrates how animals in PCOA disperse pine cones.
**Algorithm 2** Dispersing pine cone by animals**Input:** **Input:** $P_{1}$, $P_{2}$, CX, TX, FES and $FES_{max}$**Output:** Pine Cone’s position  *Dispersing pine cone by animals*: 
1: **if** $and\;(FES>P_{2}\times FES_{max},rand<0.9)$ || $and\;(FES<P1\times FES_{max},rand<0.9)$ **then**2:    Calculate $X_{initial}$ using Equation (12)3:    Run quadratic programming optimization method based on the $X_{initial}$4:    Update $X_{best}$ if solution of quadratic programming was better5:    Calculate $X_{animal}$ using Equation (13)6: **else**7:    Compute $w_{d}$ using Equation (15)8:    **if** rand < 0.5 **then**9:     Calculate $X_{animals}$ using Equation (14)10:    **else**11:     Calculate $X_{animals}$ using Equation (16)12:    **end if**13: **end if**14: **return** Pine Cone’s position

### 2.7. Assigning Pine Cones to Each Tree

Each tree in PCOA has cones that are updated exclusively near it. This approach is used to simulate the scattering of pine cones by gravity. In the first generation, every tree has an equal number of cones. However, in later generations, each tree receives the cones that are closest to it. Since most solutions converge towards the best solution through increasing generations, pines with a better position have more cones. This issue balances exploration and exploitation and estimates the absolute optimum more accuracy.

### 2.8. Flowchart of PCOA

**Figure 3:**
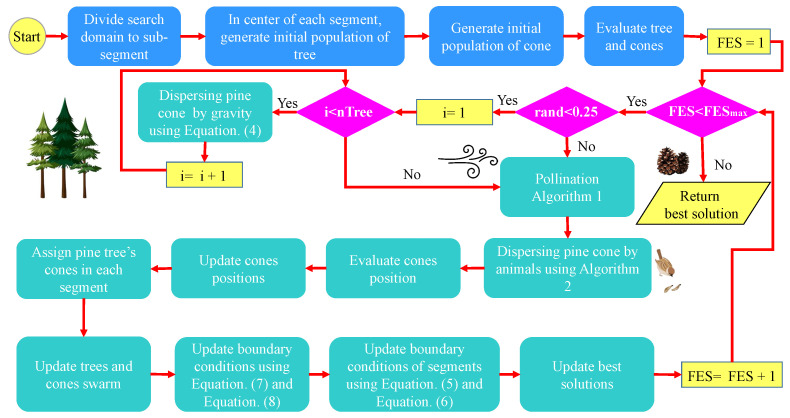
Pine Cone Optimization Algorithm (PCOA) flowchart.

In the PCOA, first, the algorithm generates the initial population. Then, it updates this population using the operators of pine cone dispersal by gravity, wind pollination, and pine cone dispersal by animals. Gravity dispersal of the pine cones and animal dispersal of the pine cones handle exploitation, and wind pollination is associated with exploration. In addition, the adaptive weight and reduced boundary conditions help the PCOA to achieve a balance between exploration and exploitation. Figure 3 shows the flowchart of PCOA.

## 3. Experimental Research Settings

PCOA was evaluated using mathematical benchmark functions and engineering problems. The mathematical benchmark functions comprise 23 classic benchmark functions, 29 CEC-BC-2017 benchmark functions in 10, 30, 50, and 100 dimensions [58], and 10 CEC-C06 2019 benchmark functions [58]. The engineering problems include 6 CEC2006 and 17 CEC2011 problems [59]. PCOA and other algorithms were implemented using Matlab R2020b, executed on Windows 10, Intel Xeon 2.2 GHz CPU, and 40 G RAM. This study uses the following assumptions to evaluate PCOA and other competitors:

- In order to achieve a fair comparison of optimization algorithms, we set the maximum number of function evaluations ($FES_{max}$) to the same value. $FES_{max}$ for the classic benchmark functions, CEC2017, CEC2006, and CEC2011 is equal to 150,000, 10,000.Dim [60], 15,000, and 150,000 [59], respectively. For CEC2019, the $FES_{max}$ for first to tenth benchmark functions are equal to 90,000, 320,000, 1,800,000, 1,000,000, 100,000, 100,000, 1,000,000, 1,000,000, 1,000,000, and 600,000, respectively.
- The researchers set the independent run of algorithms for the classic benchmark functions, CEC2017, CEC2019, and CEC2006, to 51. For CEC2011, this value was 25. These values are based on [22,59,60]. In the following subsections, more information about the experimental settings is presented.

### 3.1. Mathematical Benchmark Functions

Sixty-two benchmark functions were employed to evaluate the performance of PCOA and the other investigated algorithms. The classic benchmark functions comprise seven unimodal (1 to 7), six multi-modal (8 to 13), and ten multi-modal fixed-dimension (14 to 23) functions. The CEC2017 test suite includes two unimodal (1 and 3), seven multi-modal (4–10), ten hybrid (11–20), and ten composite (21–30) benchmark functions. Because of the unstable behavior, the second CEC2017 benchmark function was removed. Algorithms with better performance in solving unimodal and multi-modal benchmark functions have more ability in exploration and exploitation. An algorithm with more accuracy in optimizing hybrid and composite functions has more ability to escape from local optima. The global optimum for CEC2017_01 and CEC2017_03–30 are 100, 300, 400, 500, 600, 700, 800, 900, 1000, 1100, 1200, 1300, 1400, 1500, 1600, 1700, 1800, 1900, 2000, 2100, 2200, 2300, 2400, 2500, 2600, 2700, 2800, 2900, and 3000, respectively. The global optima of the classic benchmark functions and CEC2019 and their characteristics are listed in Table A1 and Table A2 in the Appendix A.

### 3.2. Engineering Design Optimization Problems

The analysis of PCOA’s and its competitors’ performance in optimizing engineering design problems involved using twenty-three problems. CEC2006 comprises a tension/compression spring [61], gear train [27], speed reducer [62], and three models of a 25-bar truss [15]. CEC2011 contains constrained problems, and several linear and non-linear constraints increase their complexity. Therefore, using these problems shows the ability of PCOA to solve engineering problems. These problems comprise parameter estimation for frequency-modulated (FM) sound waves (CEC11_01); the Lennard–Jones potential problem (CEC11_02); optimal control of a non-linear stirred tank reactor (CEC11_03); the transmission network expansion planning (TNEP) problem (CEC11_04); the large-scale transmission pricing problem (CEC11_05); the circular antenna array design problem (CEC11_06); the static economic load dispatch (ELD) problem (CEC11_07); different models of dynamic economic dispatch (DED) problems (CEC11_08–12); different models of hydrothermal scheduling problems (CEC11_13–15); the messenger: spacecraft trajectory optimization problem (CEC11_16); and the Cassini 2: spacecraft trajectory optimization problem (CEC11_17). This study uses the Lagrangian method to handle the constrained CEC2006 and CEC2011 problems.

### 3.3. PCOA Competitors

In this study, a range of well-known, new, and state-of-the-art algorithms are chosen for comparative reference to demonstrate PCOA’s performance in relation to various algorithms. Among the well-known algorithms are PSO, DE, and WOA; AVOA, RW_GWO, HHO, and gradient-based optimizer (GBO) are new algorithms; and linear population size reduction (LSHADE) and Effective Butterfly Optimizer with Covariance Matrix Adapted Retreat Phase (EBOwithCMAR) represent state-of-the-art optimization algorithms.

### 3.4. PCOA Competitors’ Parameter Settings

Table 1 tabulates the parameter settings of PCOA’s competitors. The population size and maximum number of iterations for the investigated algorithms were selected so that $FES_{max}$ was the same for them. In this table, the parameters of AVOA, RW_GWO, WOA, HHO, and GBO are fixed; no sensitivity analysis is needed. The parameters for PSO and DE are determined based on sensitivity analyses from previous studies [22,63], which had experimental settings similar to the present study. The parameters of LSHADE and EBOwithCMAR are determined by the sensitivity analyses in the studies of [63,64].

### 3.5. Analyzing the Influence of the PCOA Parameters

In this study, we analyzed the influence of the parameters of the proposed algorithm using a sensitivity analysis. The method for analyzing the influence of the PCOA parameters was as follows:

- To perform a sensitivity analysis on the PCOA, select one parameter (either N_Tree, N_Cone, N_Cycle, P1, or P2).
- Determine one value for the selected parameter.
- Execute the PCOA algorithm multiple times and compute the fitness function.
- If there is another value for the investigated parameters, go to step 3; otherwise, go to step 5.
- Normalize the obtained fitness function using the map min–max method as follows:
  (20) $$fitness_{normalized,j}=\frac{\bar{fitness_{j}}-min\left(\bar{fitness_{j}}\right)}{max\left(\bar{fitness_{j}}\right)-min\left(\bar{fitness_{j}}\right)}$$
  In Equation (20), $fitness_{normalized,j}$ is the normalized fitness function for the jth parameter, $\bar{fitness_{j}}$ is the average fitness function for the jth parameter in the nth random run. The value of n equals the number of random runs for each benchmark function or engineering problem set.
- Consider the parameter setting with a minimum $fitness_{normalized,j}$ as the desired value.
- If there is another parameter for sensitivity analysis, go to step 2. For each parameter, plot the sensitivity analysis results. The plot displays the minimum value of the fitness function as closest to zero (green color), whereas values closer to one (red color) indicate the maximum values.

### 3.6. Statistical Tests

Wilcoxon signed-rank and Friedman tests, as non-parametric tests, were employed to compare PCOA with the other investigated algorithms. The study conducted by [67] recommended the Wilcoxon signed-rank test for pair-wise comparisons. The Wilcoxon test returns the number of victories and defeats of one algorithm against the others. The Friedman test determines the algorithm’s mean rank and is used in many studies, such as [66,67].

## 4. Results

In this section, a sensitivity analysis of PCOA is carried out, and the best parameters of PCOA are reported. Then, the robustness, convergence efficiency, and time complexity of PCOA are evaluated on 62 benchmark functions and 21 engineering design problems. The benchmark functions test suite comprises 23 classical benchmark functions, 29 CEC2017 in 10, 30, 50, and 100 dimensions, and 10 CEC2019. The engineering design problems include 6 CEC2006 and 18 CEC2011.

### 4.1. Sensitivity Analysis

**Figure 4:**
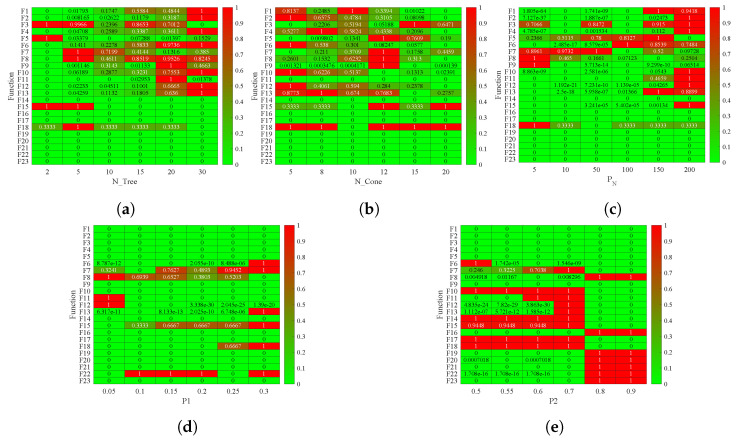
PCOA sensitivity analysis using classic benchmark functions. (**a**) N_Tree; (**b**) N_Cone; (**c**) N_Cycle; (**d**) P1; (**e**) P2.

**Figure A1:**
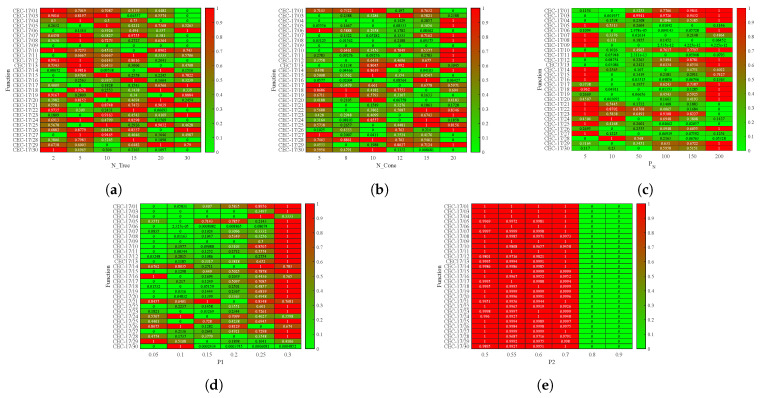
PCOA sensitivity analysis using CEC2017 benchmark functions on 10 dimensions. (**a**) N_Tree; (**b**) N_Cone; (**c**) N_Cycle; (**d**) P1; (**e**) P2.

**Figure A2:**
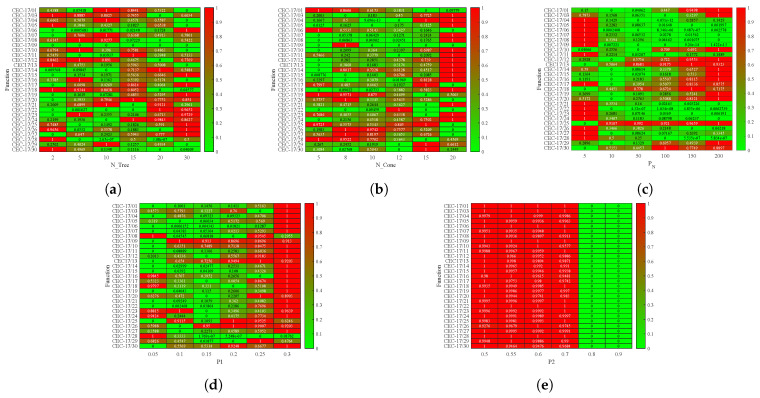
PCOA sensitivity analysis using CEC2017 benchmark functions on 30 dimensions. (**a**) N_Tree; (**b**) N_Cone; (**c**) N_Cycle; (**d**) P1; (**e**) P2.

**Figure A3:**
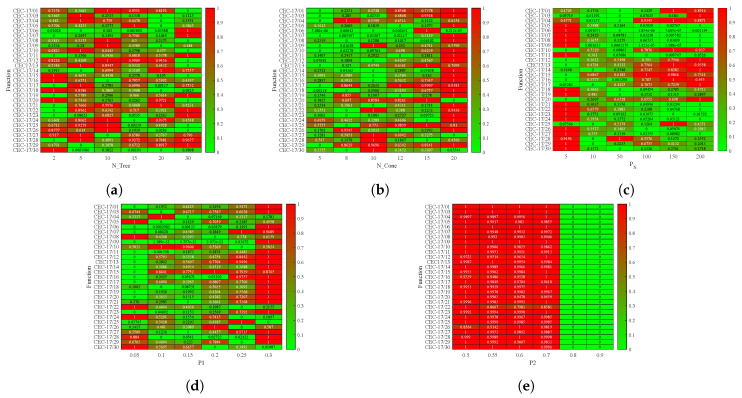
PCOA sensitivity analysis using CEC2017 benchmark functions on 50 dimensions. (**a**) N_Tree; (**b**) N_Cone; (**c**) N_Cycle; (**d**) P1; (**e**) P2.

**Figure A4:**
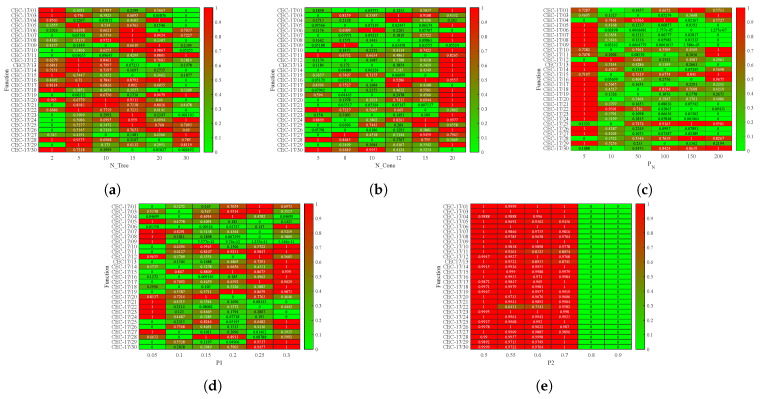
PCOA sensitivity analysis using CEC2017 benchmark functions on 100 dimensions. (**a**) N_Tree; (**b**) N_Cone; (**c**) N_Cycle; (**d**) P1; (**e**) P2.

**Figure A5:**
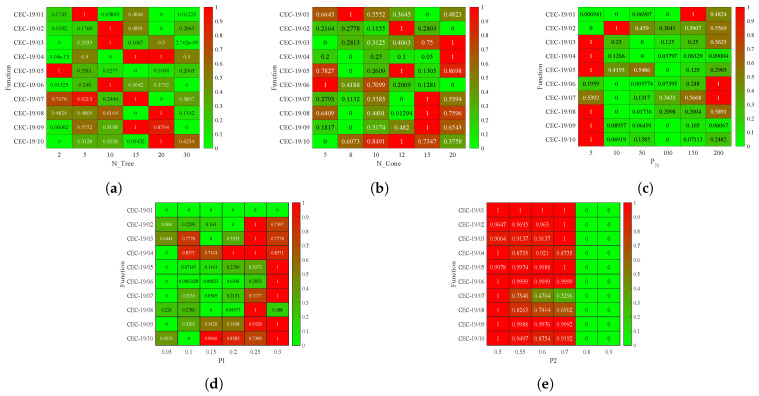
PCOA sensitivity analysis using CEC2019 benchmark functions. (**a**) N_Tree; (**b**) N_Cone; (**c**) N_Cycle; (**d**) P1; (**e**) P2.

**Figure A6:**
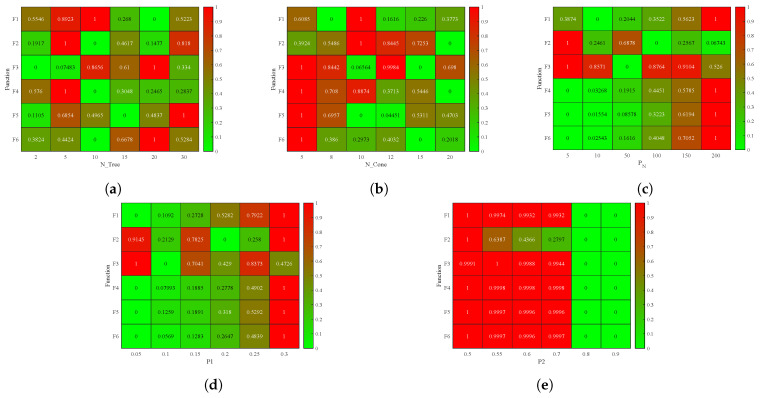
PCOA sensitivity analysis using CEC2006 benchmark functions. (**a**) N_Tree; (**b**) N_Cone; (**c**) N_Cycle; (**d**) P1; (**e**) P2.

**Figure A7:**
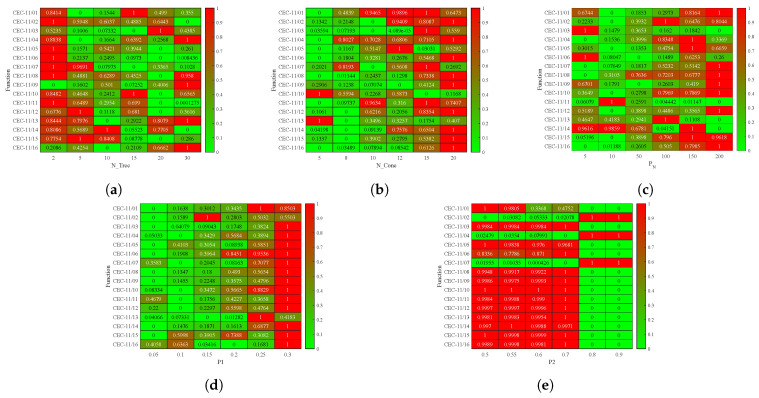
PCOA sensitivity analysis using CEC2011 benchmark functions. (**a**) N_Tree; (**b**) N_Cone; (**c**) N_Cycle; (**d**) P1; (**e**) P2.

In this study, the parameters of PCOA are obtained using a sensitivity analysis. The sensitivity analysis method presented by [22] is employed. This method uses the min–max normalization method to scale the objective function between 0 and 1. The results are clearly shown using this method. Figure 4 and Figure A1, Figure A2, Figure A3, Figure A4, Figure A5, Figure A6 and Figure A7 show the results of the PCOA sensitivity analysis. These figures clearly show the best results (represented by a green color) and the worst results (represented by a red color) which are obtained by employing the method proposed by [22] and using the min–max normalization method to scale the objective function between 0 and 1. As seen for the classic benchmark functions, CEC2019 and CEC2006, the best N_Tree was equal to 2. In contrast, the best N_Tree for CEC2017 was equal to 2 and 30. About CEC2006 and CEC2011, the best value of N_Tree was 20 and 10, respectively. About N_Cone, the best values for the classic benchmark functions, CEC2017, CEC2019, CEC2006, and CEC2011, were equal to 20, 5, 5, 20, and 5, respectively. The best values of N_Cycle for the classic benchmark functions, CEC2017, CEC2019, CEC2006, and CEC2011, were 5, 50, 100, 5, and 10, respectively. Considering P1, the best value was 0.05. The best value for P2 was equal to 0.8. Regarding all results, the default values for N_Tree, N_Cone, N_Cycle, P1, and P2 were 2, 5, 50, 0.05, and 0.8, respectively.

### 4.2. The Benchmark Set and Compared Algorithms

**Figure 5:**
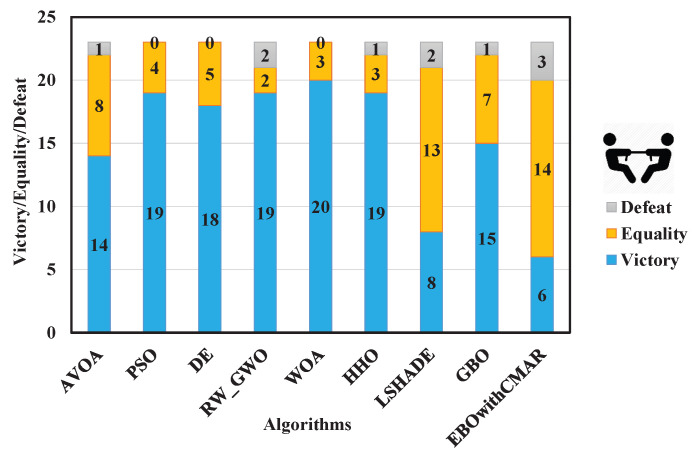
Comparison of Wilcoxon test for PCOA and other algorithms using the classic benchmark functions over 51 independent runs and 150,000 NFES.

**Figure 6:**
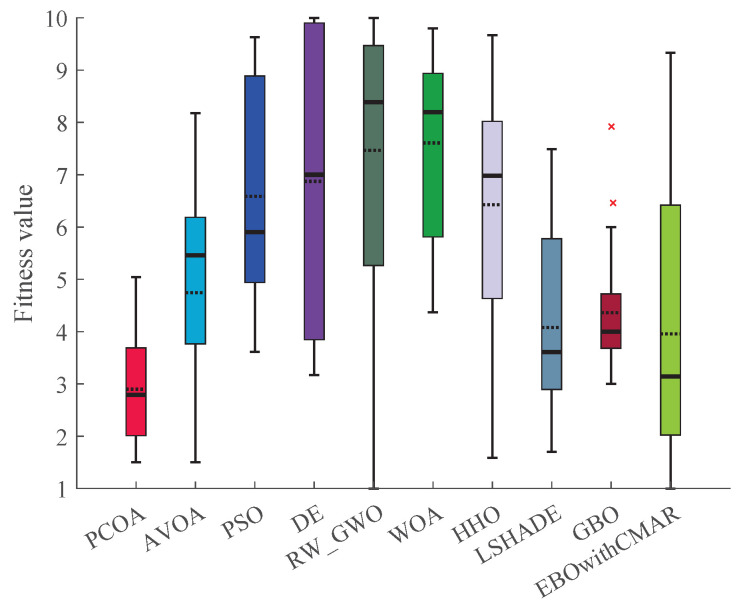
Comparison of Friedman ranking for PCOA and other algorithms using the classic benchmark functions over 51 independent runs and 150,000 NFES.

A comparison was made between PCOA and AVOA, PSO, DE, RW_GWO, WOA, HHO, LSHADE, GBO, and EBOwithCMAR to assess their ability to solve the classic benchmark functions, CEC2017 and CEC2019. Table A3 presents the minimum (min), mean, median, maximum (max), and standard deviation (std) results of PCOA and other approaches for solving the classical benchmark functions. PCOA reached the global optimum for F1–F6, F9, F11, F17–F18, F20, and F21. Regarding the remaining classical benchmark functions, PCOA approached the global optimum with high accuracy. In Figure 5, the Wilcoxon test determines the number of victories, equalities, and defeats of PCOA compared to other competitors. According to the results, the numbers of PCOA’s victories against AVOA, PSO, DE, RW_GWO, WOA, HHO, LSHADE, GBO, and EBOwithCMAR were equal to 14, 19, 18, 19, 20, 19, 8, 15, and 6, respectively. Based on the mentioned results, the superiority of PCOA compared to AVOA, PSO, DE, RW_GWO, WOA, HHO, and GBO is clear in optimizing the investigated classical benchmark functions. The equality results in this figure show that PCOA had 13 and 14 equalities versus LSHADE and EBOwithCMAR. Hence, in the classical benchmark functions, PCOA’s results were comparable to those for LSHADE and EBOwithCMAR. In Figure 6, the Friedman ranking test is employed to show the rank of each algorithm. The Friedman ranking analysis shows that PCOA was ranked first. Meanwhile, EBOwithCMAR and LSHADE rank second and third, respectively. The PCOA’s rank (2.90) was 28.92% and 26.77% less than LSHADE and EBOwithCMAR.

**Figure 7:**
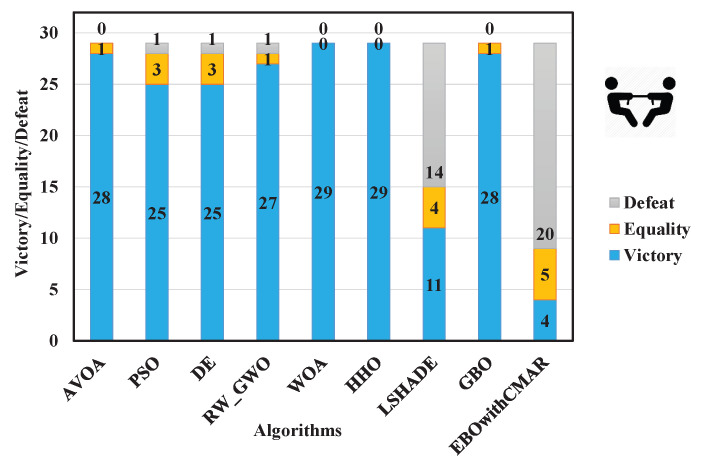
Comparison of Wilcoxon test for PCOA and other algorithms using the 10D CEC2017 benchmark functions over 51 independent runs and 100,000 NFES.

**Figure 8:**
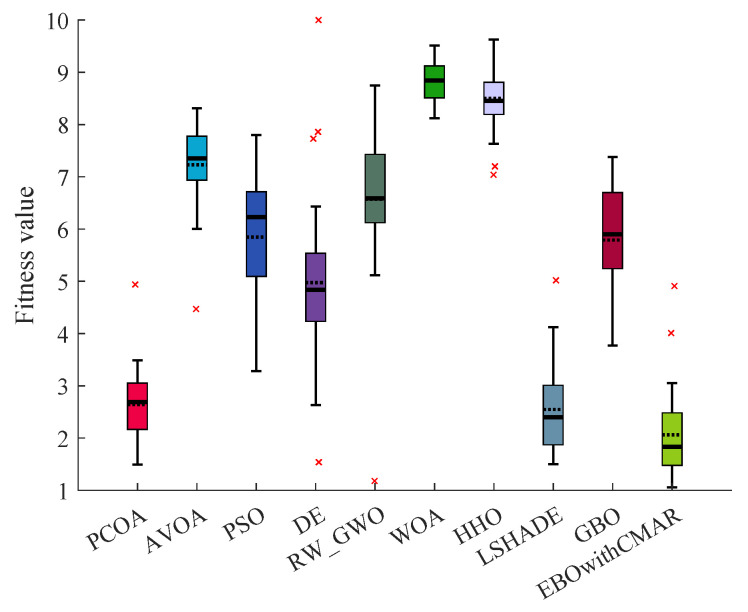
Comparison of Friedman ranking for PCOA and other algorithms using the 10D CEC2017 benchmark functions over 51 independent runs and 100,000 NFES.

Table A4 lists the statistical criteria of PCOA and other approaches for optimizing CEC2017 over 10 dimensions. Regarding unimodal benchmark functions (CEC17_01 and CEC17_02), PCOA could consistently find a global optimum solution. Regarding multi-modal benchmark functions (CEC17_04–10), PCOA could consistently find global optimum solutions for CEC17_04–06 and CEC17_08–09. PCOA approached the global optimum solutions with reasonable accuracy in the remaining multi-modal functions. Regarding hybrid benchmark functions (CEC17_11–20), PCOA could compute the global optimum of CEC17_11 consistently. In the remaining hybrid functions, the solutions of PCOA were close to the global optimum, and sometimes PCOA approached the global optimum solutions with 100% accuracy. About the composite benchmark functions (CEC17_21–30), PCOA became trapped in local optimum solutions in solving CEC17_21, CEC17_23–25, CEC17_27, and CEC17_29–30. This is an issue because of the many local optimums and complex structures of the mentioned functions. However, PCOA’s results were close to the optimum solutions in the remaining composite functions. PCOA could find the global optima of CEC17_22, CEC17_26, and CEC17_28 in some runs. A comparison of the results of PCOA and its competitors based on the Wilcoxon test (Figure 7) showed that PCOA had 28 victories over AVOA, 25 victories over PSO and DE, 27 victories over RW_GWO, 29 victories over WOA and HHO, and 28 victories over GBO. Compared to LSHADE, PCOA had 11 victories and 4 equalities. The number of victories and equalities of PCOA against EBOwithCMAR were equal to 4 and 5, respectively. The Friedman test results (Figure 8) showed that PCOA had a lower rank than other approaches, followed by LSHADE and EBOwithCMAR.

**Figure 9:**
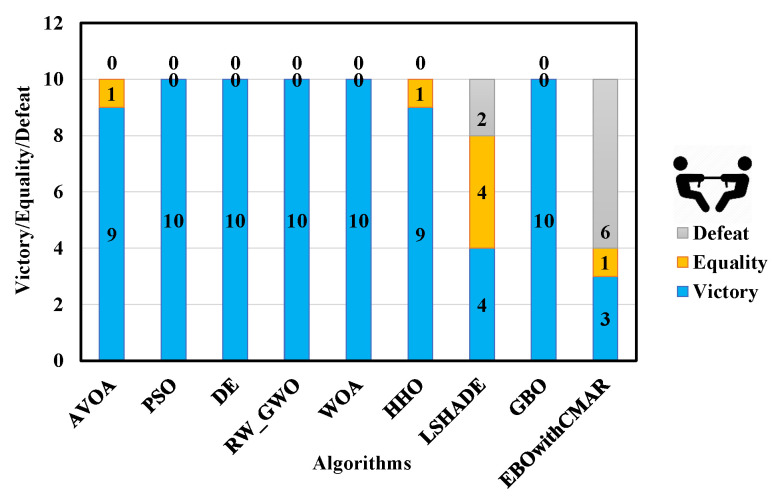
Comparison of Wilcoxon test for PCOA and other algorithms using the CEC2019 benchmark functions over 51 independent runs.

**Figure 10:**
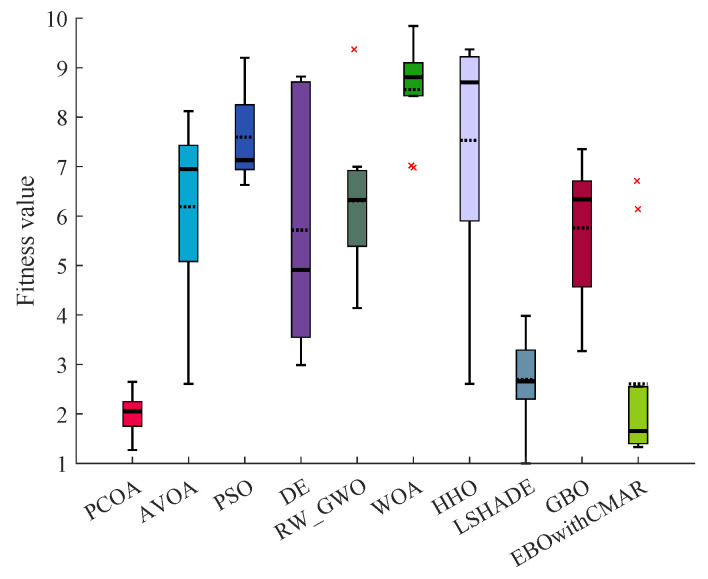
Comparison of Friedman ranking for PCOA and other algorithms using the CEC2019 benchmark functions over 51 independent runs.

**Figure A8:**
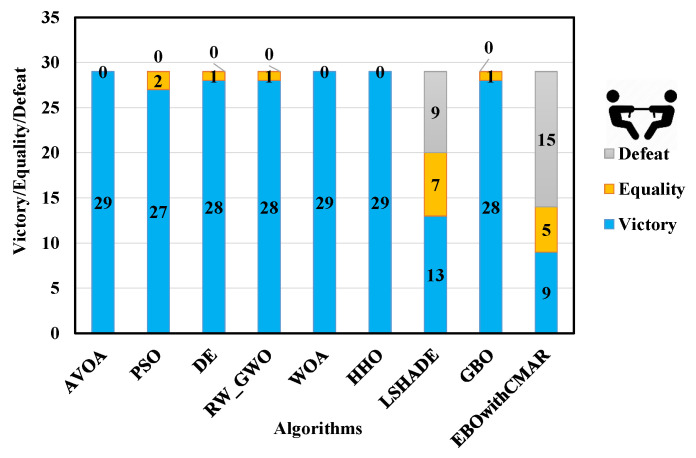
Comparison of Wilcoxon test for PCOA and other algorithms using the 30D CEC2017 benchmark functions over 51 independent runs and 300,000 NFES.

**Figure A9:**
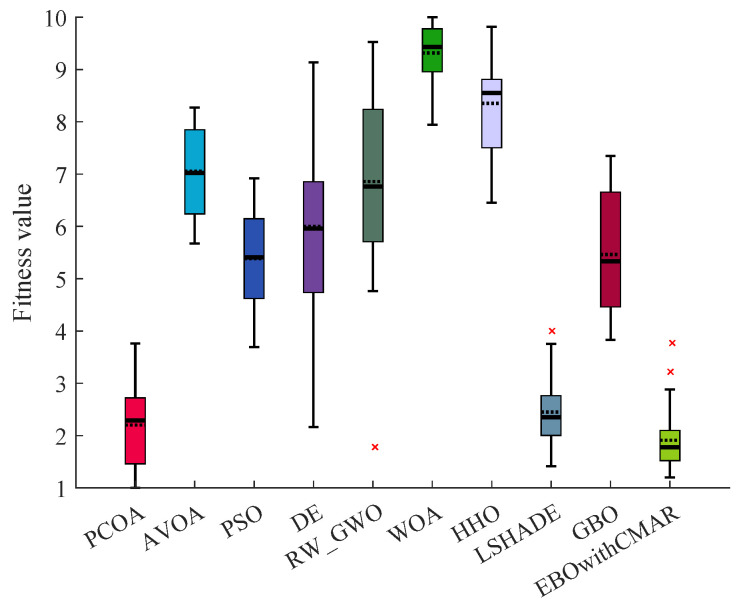
Comparison of Friedman ranking for PCOA and other algorithms using the 30D CEC2017 benchmark functions over 51 independent runs and 300,000 NFES.

**Figure A10:**
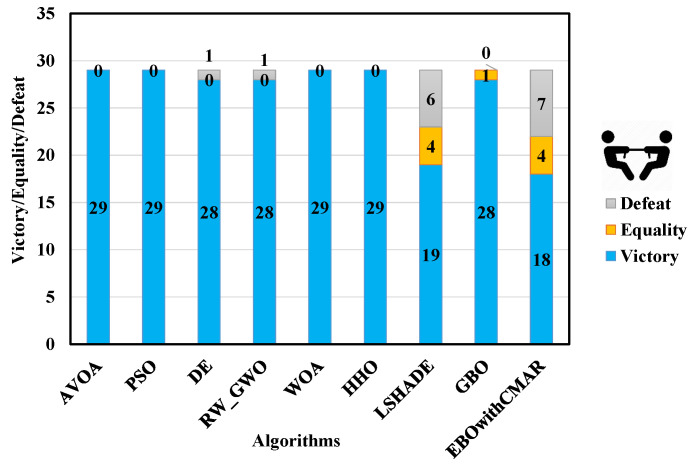
Comparison of Wilcoxon test for PCOA and other algorithms using the 50D CEC2017 benchmark functions over 51 independent runs and 500,000 NFES.

**Figure A11:**
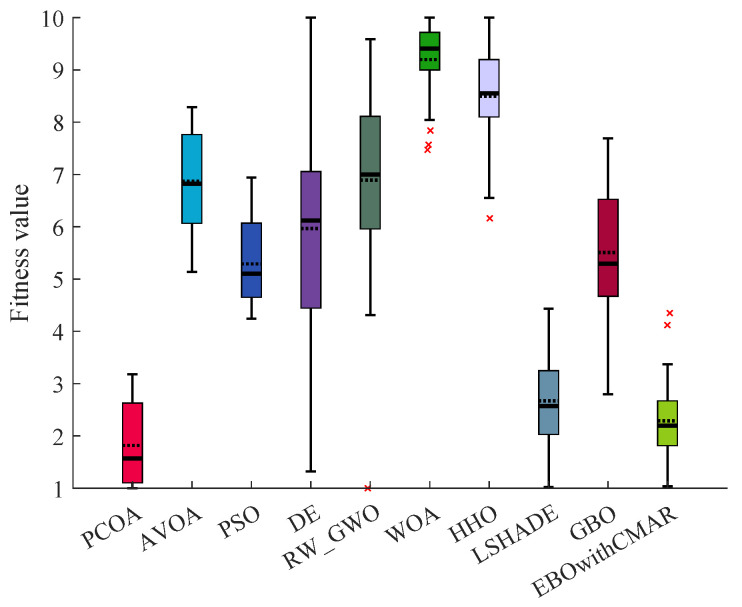
Comparison of Friedman ranking for PCOA and other algorithms using the 50D CEC2017 benchmark functions over 51 independent runs and 500,000 NFES.

**Figure A12:**
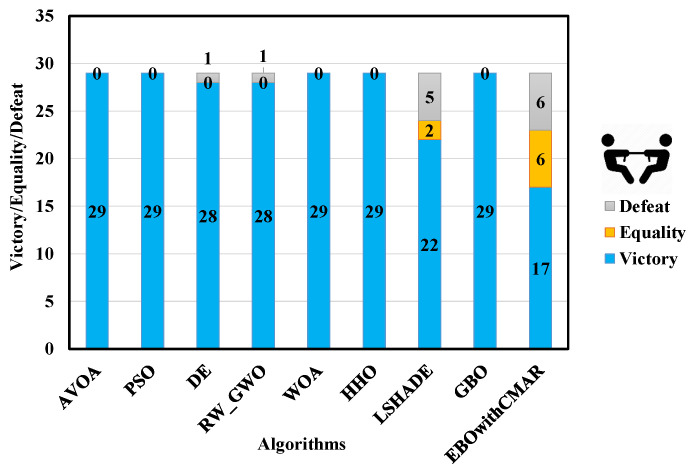
Comparison of Wilcoxon test for PCOA and other algorithms using the 100D CEC2017 benchmark functions over 51 independent runs and 1,000,000 NFES.

**Figure A13:**
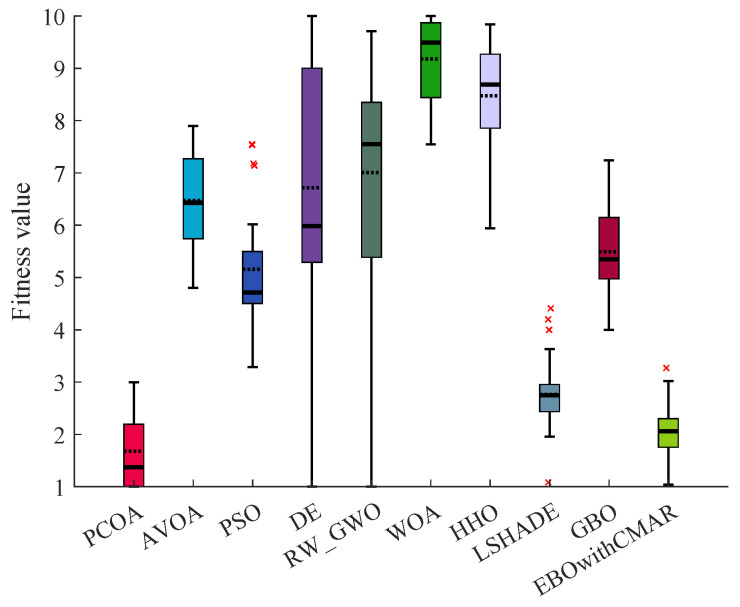
Comparison of Friedman ranking for PCOA and other algorithms using the 100D CEC2017 benchmark functions over 51 independent runs and 1,000,000 NFES.

Table A5 tabulates the results of solving CEC2017 over 30 dimensions using PCOA, AVOA, PSO, DE, RW_GWO, WOA, HHO, LSHADE, GBO, and EBOwithCMAR. On the unimodal benchmark functions, PCOA could find global optimum solutions for them in all runs. Regarding the multi-modal benchmark functions, PCOA consistently obtained the global optimum solutions of CEC17_04, CEC17_06, and CEC17_09. In the remaining unimodal functions, PCOA estimated optimum solutions with good precision. Regarding the hybrid benchmark functions, PCOA could find global optimum solutions of CEC17_11 and, in the remaining functions, approached the optimum solutions with reasonable accuracy. Regarding the composite benchmark functions, PCOA was trapped in a local optimum because of the large number of local optimum solutions and the challenging structure of these functions. However, the accuracy of PCOA in solving the composite functions was comparable with other algorithms. According to the Wilcoxon test results for CEC2017 over 30 dimensions (Figure A8), PCOA won in 29, 27, 28, 28, 29, 29, 13, 28, and 9 functions, respectively. According to the Friedman test results (Figure A9), PCOA achieved the highest rank, followed by EBOwithCMAR. Table A6 displays the results of solving CEC2017 over 50 dimensions using PCOA, AVOA, DE, RW_GWO, WOA, HHO, LSHADE, GBO, and EBOwithCMAR. Based on the results, for unimodal CEC2017, PCOA achieved the global optimum solutions consistently. Regarding multi-modal CEC2017, PCOA reached the global optimum of CEC17_04, CEC17_06, and CEC17_09 with an accuracy of 100%. PCOA’s precision in approximating global optimum solutions in the remaining multi-modal CEC2017 was reasonable compared to other competitors. Regarding hybrid and composite CEC2017, PCOA had a challenge finding global optimum solutions. However, the results of PCOA competed with the other investigated algorithms. The Wilcoxon test (Figure A10) and the Friedman test (Figure A11) showed the superiority of PCOA over the other competitors. PCOA was the winner in 19 and 18 functions over LSHADE and EBOwithCMAR. In comparison with the state-of-the-art algorithms, the Friedman’s ranking of PCOA was 31.91% and 20.62% better than LSHADE and EBOwithCMAR. Table A7 compares PCOA and its competitors using CEC2017 over 100 dimensions. PCOA obtained global optimum solutions (CEC17_01 and CEC17_02) and some multi-modal CEC2017 (CEC17_04, CEC17_06, and CEC17_09). In the remaining CEC2017, PCOA performed better than the other competitors in most conditions. The Wilcoxon test results (Figure A12) showed that PCOA had 29 victories over AVOA, PSO, WOA, HHO, and GBO, 28 victories over DE and RW_GWO, 22 victories over LSHADE, and 17 victories over EBOwithCMAR. Figure A13 shows the Friedman ranking results in solving CEC2017 with 100 dimensions. As seen, PCOA took the first rank (1.68), which was 39.37% and 18.43% less than the third- (LSHADE) and second- (EBOwithCMAR) best algorithms. Considering the CEC2017 benchmark functions in all dimensions, PCOA had superiority over AVOA, PSO, DE, RW_GWO, WOA, HHO, and GBO. In 10 and 30 dimensions, the results of PCOA were close to the winners of CEC2017, LSHADE and EBOwithCMAR, while in the remaining dimensions, PCOA had more accuracy. Regarding the quality of PCOA’s results, it can be recognized that PCOA’s results were comparable to those of the other investigated algorithms. Table A8 presents the min, mean, median, max, and std results of solving the CEC2019 benchmark functions using PCOA and the other approaches. Based on the findings of this table, the proposed algorithm performed better than AVOA, PSO, DE, RW_GWO, WOA, HHO, LSHADE, GBO, and EBOwithCMAR. PCOA could find a global optimum for CEC19_01 and CEC19_03–CEC19_06. For other benchmark functions, the PCOA results were comparable with other investigated approaches. The Wilcoxon test in Figure 9 revealed that PCOA had more victories over AVOA, PSO, DE, RW_GWO, WOA, HHO, LSHADE, and GBO. In comparison with EBOwithCMAR, PCOA had three victories and one equality. According to the Friedman ranking, PCOA achieved the first rank. The Friedman ranking of PCOA (Figure 10) was 24.36% and 21.69%, better than LSHADE (the third-best algorithm) and EBOwithCMAR (the second-best algorithm).

According to the obtained results for the benchmark functions, in most benchmark functions PCOA had better performance than AVOA, PSO, DE, RW_GWO, WOA, HHO, LSHADE, GBO, and EBOwithCMAR. Comparing statistical criteria over 51 random runs (min, mean, median, max, and std), the Wilcoxon and Friedman ranking tests confirm this.

### 4.3. The Engineering Problems and the Compared Algorithms

**Figure 11:**
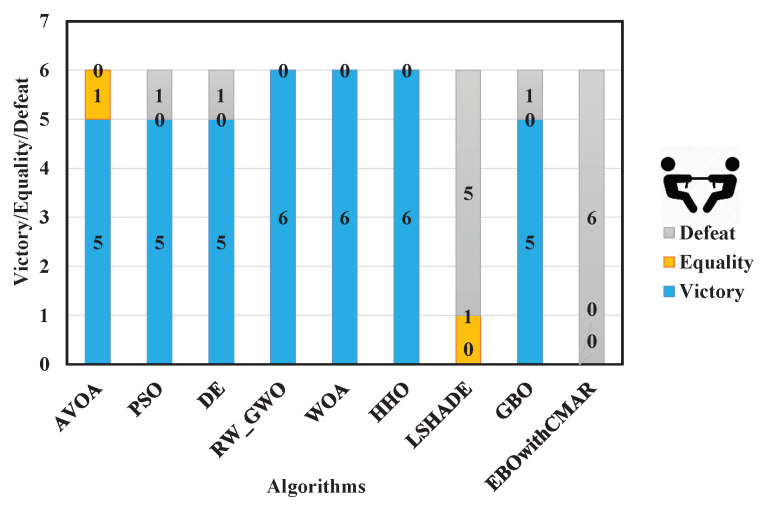
Comparison of Wilcoxon test for PCOA and other algorithms using the CEC2006 benchmark functions over 51 independent runs.

**Figure 12:**
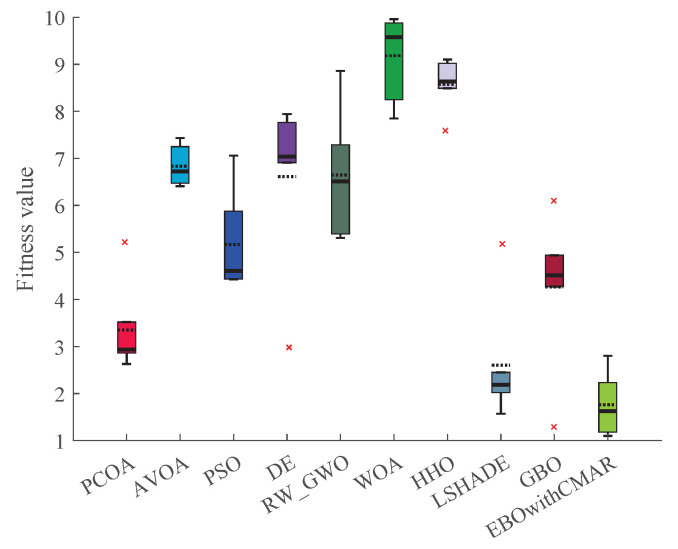
Comparison of Friedman ranking for PCOA and other algorithms using the CEC2006 benchmark functions over 51 independent runs.

A summary of the statistical results (min, mean, median, max, and std) of the PCOA and other approaches to solving the CEC2006 engineering problems is presented in Table A9. The results of this table are for 51 random runs. PCOA performed better than AVOA, PSO, DE, RW_GWO, WOA, HHO, and GBO in solving CEC06_01–02 and CEC06_04–06. For CEC06_03, the results of PCOA were better than AVOA, RW_GWO, WOA, and HHO. According to the Wilcoxon test (Figure 11) and Friedman test (Figure 12), PCOA was better than AVOA, PSO, DE, RW_GWO, WOA, HHO, and GBO.

**Figure 13:**
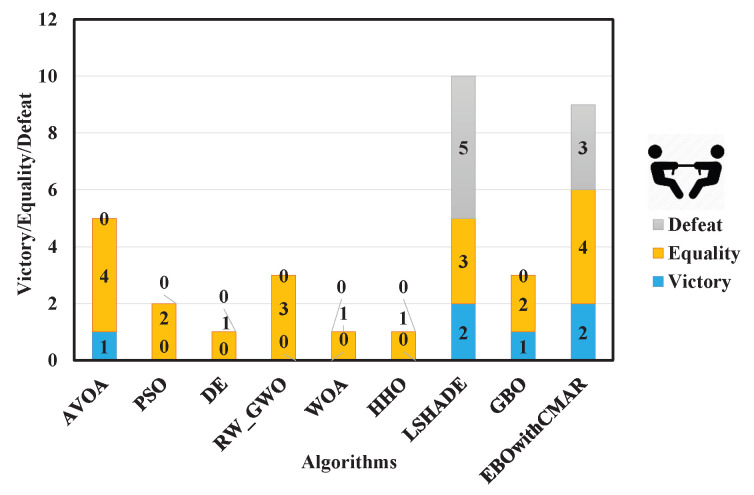
Comparison of Wilcoxon test for PCOA and other algorithms using the CEC2011 benchmark functions over 25 independent runs.

**Figure 14:**
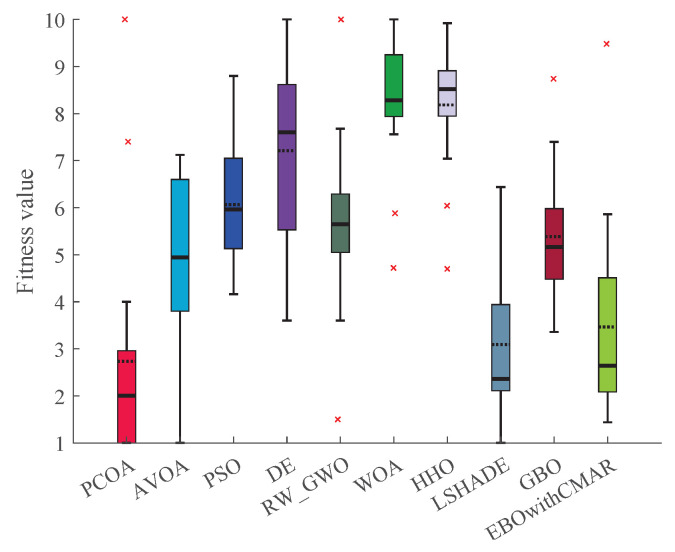
Comparison of Friedman ranking for PCOA and other algorithms using the CEC2011 benchmark functions over 25 independent runs.

Table A10 displays the optimization results for CEC2011 by PCOA and its competitors. PCOA outperformed its competitors in solving CEC11_03, CEC11_05, CEC11_07, CEC11_11–13, and CEC11_15–17. Considering the Wilcoxon test results (Figure 13), PCOA had 11, 14, 15, 13, 15, 15, 11, 13, and 10 victories over AVOA, PSO, DE, RW_GWO, WOA, HHO, LSHADE, GBO, and EBOwithCMAR. Regarding the Friedman test (Figure 14), the proposed algorithm was ranked first (rank = 1.94). LSHADE achieved the second-ranking (rank = 2.60) while EBOwithCMAR obtained the third-ranking (rank = 3.25). Therefore, the results show that PCOA performed competitively compared to the other investigated approaches in optimizing engineering problems.

### 4.4. PCOA Convergence Analysis

**Figure 15:**
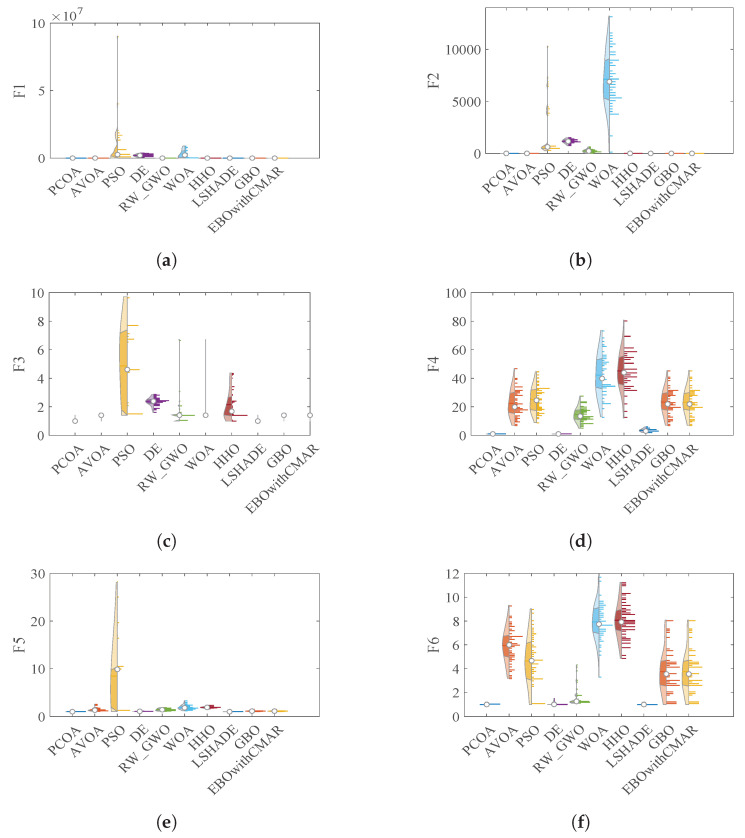

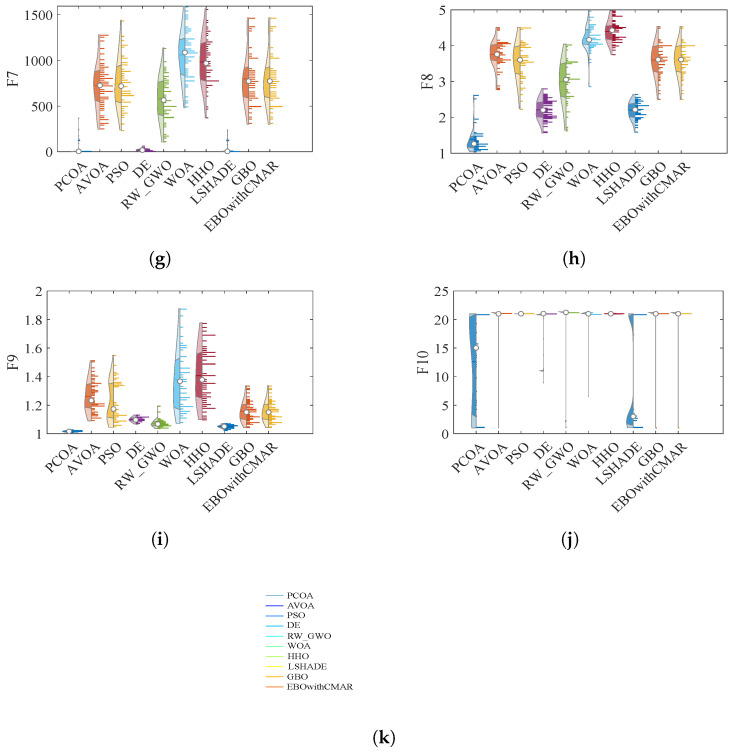
Violin plots of PCOA and competitors in optimizing CEC2019 benchmark functions. (**a**) CEC19_01; (**b**) CEC19_02; (**c**) CEC19_03; (**d**) CEC19_04; (**e**) CEC19_05; (**f**) CEC19_06; (**g**) CEC19_07; (**h**) CEC19_08; (**i**) CEC19_09; (**j**) CEC19_10; (**k**) Legend.

**Figure 16:**
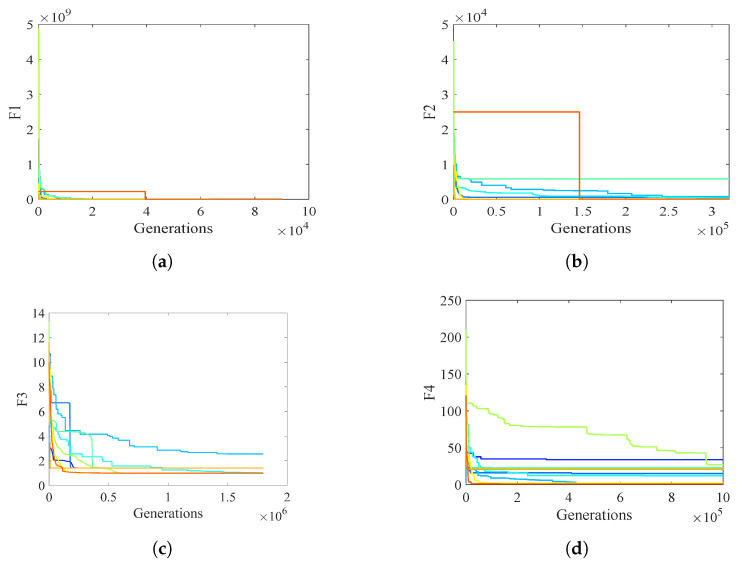

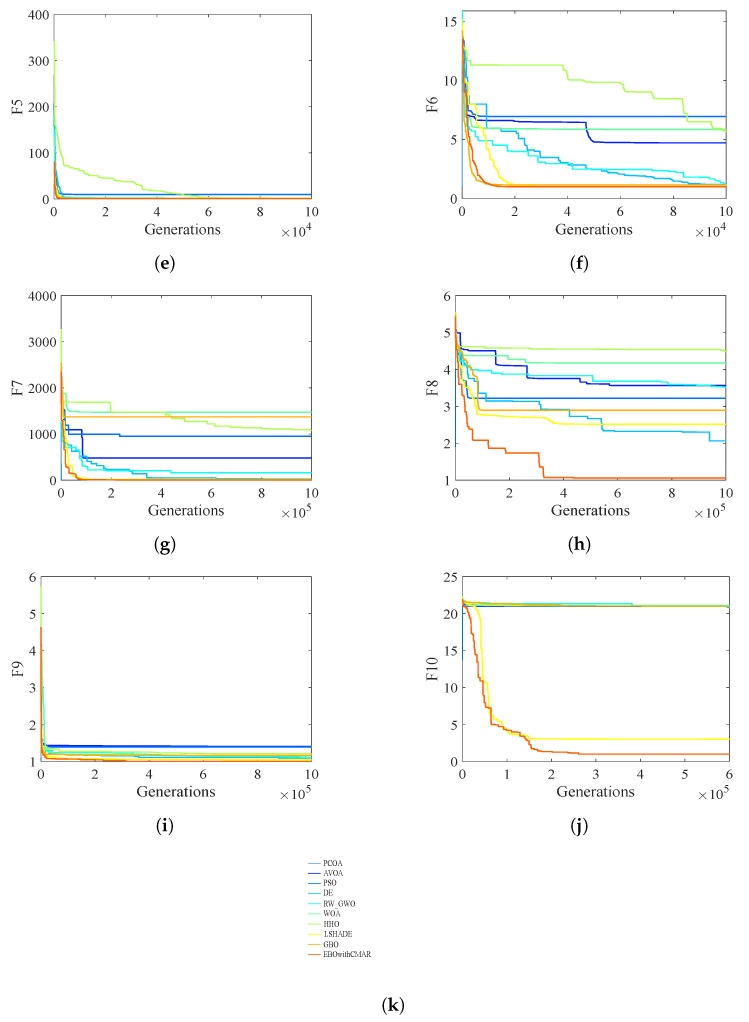
Convergence plots of PCOA and competitors in optimizing CEC2019 benchmark functions. (**a**) CEC19_01; (**b**) CEC19_02; (**c**) CEC19_03; (**d**) CEC19_04; (**e**) CEC19_05; (**f**) CEC19_06; (**g**) CEC19_07; (**h**) CEC19_08; (**i**) CEC19_09; (**j**) CEC19_10; (**k**) Legend.

This subsection analyzes the PCOA’s and competitors’ convergence efficacies using violin plots (Figure 15) and convergence curves (Figure 16). The violin plot shows the distribution of the algorithm’s results. A broader violin shows more uncertainty and less quality in this plot, and vice versa. Based on the violin plots shown in Figure 15, PCOA converged to optimum values in almost all conditions. The difference between the maximum and minimum solutions of PCOA was significantly less than other algorithms. This issue shows that the results of PCOA are consistent. Considering Figure 16, PCOA had a reasonable convergence speed compared to the competitors. The following equations are used to ensure that algorithms converge.

(21) $$Conv_{R}=|\frac{Obj_{opt}-\vec{Obj}}{Obj_{opt}-Obj_{0}}|$$

In Equation (20), $Obj_{opt}$ is globally optimal, the $\vec{Obj}$ is the objective function in the last generation, and $Obj_{0}$ is the objective function in the initial generation.

Table 2 shows the $Conv_{R}$ results for PCOA, AVOA, PSO, DE, RW_GWO, WOA, HHO, LSHADE, GBO, and EBOwithCMAR on distinct problems. According to the results of solving the classic, CEC2017, CEC2019, CEC2006, and CEC2011 functions, convergence is guaranteed in almost all conditions for PCOA. PCOA’s convergence rate ($Conv_{R}$) competes with other algorithms.

### 4.5. Complexity Evaluation

The complexity evaluation is carried out by estimating the $T_{0}$, $T_{1}$, and $T_{2}$ values. The computation time of calculating mathematical functions relates to $T_{0}$. $T_{1}$ is the computation time of calculating the 18th CEC2017 on 200,000 evaluations and all dimensions. $T_{2}$ is the average computation time for executing CEC17_18 with 200,000 evaluations five times. The time complexity is as follows:

(22) $$T_{Complexity}=\frac{T_{2}-T_{1}}{T_{0}}$$

Table 3 shows the time complexities of PCOA, AVOA, PSO, DE, RW_GWO, WOA, HHO, LSHADE, GBO, and EBOwithCMAR. In 10 and 30 dimensions, the algorithms’ complexities were close to each other. However, as the dimensions increased, the time complexity of the PCOA and the state-of-the-art algorithms increased, which is due to the greater efforts of these algorithms in finding optimal solutions to the problem.

## 5. Discussion

**Figure 17:**
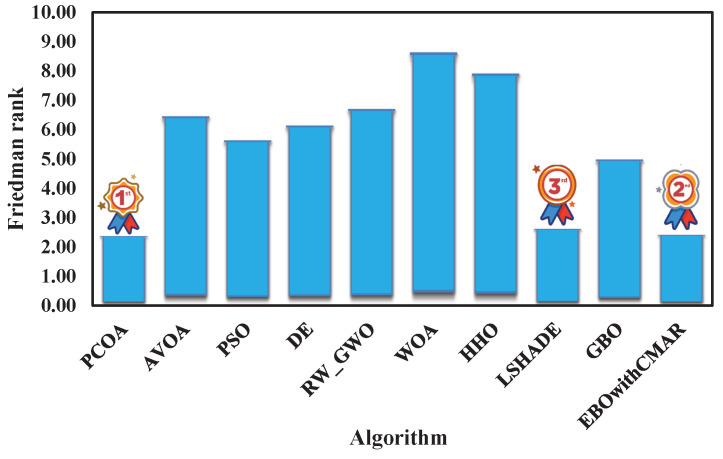
Average Friedman ranking for eighty-five benchmark functions and engineering problems.

Figure 17 shows the average of the Friedman’s ranking results for all eighty-six benchmark functions. As seen in Figure 17, PCOA, EBOwithCMAR, and LSHADE achieved ranks of 2.42, 2.46, and 2.67, respectively, placing them in the first, second, and third positions. Thus, in terms of accuracy, PCOA performed better than well-known optimization algorithms (PSO, DE, and WOA), new optimization algorithms (AVOA, RW_GWO, HHO, and GBO), and state-of-the-art algorithms (LSHADE and EBOwithCMAR). Regarding the convergence efficacy and time complexity, PCOA had competitive results compared to the other optimization algorithms. The outstanding performance of PCOA is for the following reasons:

- Using pollination operators in Equations (12)–(14) leads to good exploration and convergence to near the space of the global optimum.
- Considering new operators in pine cone dispersal by animals and gravity leads to an approximate global optimum with reasonable accuracy.
- Clustering the search domain into sub-clusters and shrinking the search space increases the chance of finding the global optimum faster and more accurately.
- Using adaptive weight creates a good balance between the exploration and exploitation ability.
- Employing Levy flight distribution and a mathematical optimizer generates diverse solutions and helps PCOA to approximate optimum solutions.

Although PCOA performed better than the other investigated algorithms, in some problems other algorithms performed better. This issue can be explained by the “no free lunch in search and optimization” theorem [1]. According to this theorem, each optimization algorithm performs better than others in some problems.

## 6. Conclusions

The present research proposed a new optimization algorithm called the Pine Cone Optimization Algorithm (PCOA). This algorithm works based on the pine tree’s reproduction methods. PCOA benefits from new operators for pollination and cone dispersal by gravity and animals. The utilization of sub-clustering methods within the search domain, coupled with the reduction in the search domain and the incorporation of adaptive weights, leads to a significant increase in the efficacy of the PCOA algorithm. This approach balances exploration and exploitation capabilities and enhances the algorithm’s performance. The performance of this algorithm was investigated in different mathematical benchmark functions and engineering optimization problems. The mathematical benchmark test suite included 23 classic benchmark functions, 29 CEC2017, and 10 CEC2019. There were six CEC2006 and seventeen CEC2011 problems in engineering optimization. The results showed that PCOA was superior to well-known optimization algorithms (PSO, DE, and WOA) and new optimization algorithms (AVOA, RW_GWO, HHO, and GBO). In addition, PCOA was a competitor for state-of-the-art algorithms (LSHADE and EBOwithCMAR). The Friedman rank for PCOA in solving all the test suite problems was 2.42, 1.68 and 9.42 percent better than EBOwithCMAR (second-best algorithm) and LSHADE (third-best algorithm). For executing PCOA, a sensitivity analysis is needed. However, in this study, the researchers reported the default values of the PCOA parameters. Users can use these default parameters to run PCOA with no sensitivity analysis. In order to use this algorithm, programming language software is needed. PCOA has a high potential for solving various complex science and engineering problems.

## Figures and Tables

**Table 1:** Parameter settings of PCOA’s competitors.

Algorithm	Parameters	Parameter Values
AVOA	$L_{1}$, $L_{2}$, *K*, $P_{1}$, $P_{2}$, $P_{3}$	0.8, 0.2, 2.5, 0.6, 0.4, and 0.6 [65]
PSO	$C_{1}$, $C_{2}$, inertia weight	2, 2, linearly decreasing from 0.9 to 0.1 [66]
DE	Lower bound of scaling factor ($\beta_{min}$), upper bound of scaling factor ($\beta_{max}$), crossover probability	0.2, 0.8, 0.2
RW_GWO	Convergence parameter (a)	Linearly decreasing from 2 to 0
WOA	Convergence parameter (a)	Linearly decreasing from 2 to 0
HHO	β	1.5
LSHADE	Archive size rate, initial population size, top individuals present, memory size	2, 20, 0.1, 5
GBO	$B_{min}$, $B_{max}$, Pr	0.2, 1.2, 0.5
EBOwithCMAR	Maximum primary population size, minimum primary population size, maximum secondary population size, minimum secondary population size, memory size, third population size, local search probability	18.dim, 4, 46.8.dim, 10, 6, 4+3.log(dim), 0.1

**Table 2:** Convergence rate analysis of PCOA and competitors using the classic, CEC2017, CEC2019, CEC2006, and CEC2011 benchmark functions.

Function	PCOA	AVOA	PSO	DE	RW_GWO	WOA	HHO	LSHADE	GBO	EBOwithCMAR
F1	1.00	1.00	0.99	0.97	1.00	1.00	1.00	0.99	1.00	0.99
F2	1.00	1.00	1.00	0.99	1.00	1.00	1.00	1.00	1.00	0.99
F3	1.00	0.99	0.98	0.55	0.99	0.81	1.00	0.99	1.00	0.99
F4	1.00	0.96	0.93	0.75	0.99	0.79	1.00	0.97	1.00	0.98
F5	1.00	1.00	1.00	0.98	1.00	1.00	1.00	1.00	1.00	0.99
F6	1.00	1.00	1.00	1.00	1.00	1.00	1.00	1.00	1.00	1.00
F7	1.00	1.00	1.00	0.98	1.00	1.00	1.00	1.00	1.00	1.00
F8	1.00	1.00	1.00	1.00	1.00	1.00	1.00	1.00	1.00	1.00
F9	1.94	0.56	0.43	0.45	0.53	0.59	0.58	0.49	0.46	1.22
F10	1.00	0.96	0.89	0.89	0.99	0.99	1.00	0.97	1.00	-
F11	1.00	1.00	1.00	1.00	1.00	1.00	1.00	1.00	1.00	1.00
F12	1.00	1.00	1.00	1.00	1.00	1.00	1.00	1.00	1.00	1.00
F13	1.00	1.00	1.00	0.99	1.00	1.00	1.00	1.00	1.00	1.00
F14	1.00	1.00	0.99	1.00	1.00	1.00	1.00	0.99	0.99	1.00
F15	1.00	1.00	1.00	1.00	1.00	1.00	1.00	1.00	1.00	1.00
F16	1.00	1.00	1.00	1.00	1.00	1.00	1.00	1.00	1.00	1.00
F17	1.00	1.00	1.00	1.00	1.00	1.00	1.00	1.00	1.00	1.00
F18	1.00	1.00	1.00	1.00	1.00	1.00	1.00	1.00	1.00	1.00
F19	1.00	1.00	1.00	1.00	1.00	1.00	1.00	1.00	1.00	1.00
F20	1.00	1.00	1.00	1.00	1.00	1.00	1.00	1.00	1.00	1.00
F21	1.00	1.00	1.00	1.00	1.00	1.00	1.00	1.00	1.00	1.00
F22	1.00	1.00	1.00	1.00	1.00	1.00	1.00	1.00	1.00	1.00
F23	1.00	1.00	1.00	1.00	1.00	1.00	1.00	1.00	1.00	1.00
CEC17_01	0.99	1.00	1.00	0.99	0.99	0.99	0.96	1.00	1.00	1.00
CEC17_03	0.77	0.99	0.95	0.96	0.97	0.98	0.94	0.99	0.99	0.99
CEC17_04	0.98	0.99	1.00	0.99	0.99	0.99	0.98	1.00	1.00	1.00
CEC17_05	0.95	0.80	0.87	0.74	0.75	0.76	0.64	0.91	0.82	0.95
CEC17_06	0.96	0.79	0.92	0.98	0.85	0.66	0.56	0.99	0.89	1.00
CEC17_07	0.94	0.88	0.96	0.91	0.90	0.86	0.82	0.96	0.86	0.95
CEC17_08	0.94	0.82	0.88	0.73	0.74	0.76	0.70	0.92	0.84	0.95
CEC17_09	0.98	0.91	0.98	0.96	0.96	0.89	0.89	1.00	0.95	1.00
CEC17_10	0.75	0.69	0.77	0.40	0.48	0.63	0.60	0.73	0.78	0.77
CEC17_11	0.97	0.99	1.00	0.98	0.98	0.99	0.97	0.99	1.00	0.99
CEC17_12	0.90	1.00	1.00	0.99	0.99	1.00	0.99	1.00	1.00	1.00
CEC17_13	0.97	1.00	1.00	1.00	1.00	1.00	0.99	1.00	1.00	1.00
CEC17_14	0.88	0.99	0.99	0.98	1.00	1.00	0.99	0.99	1.00	1.00
CEC17_15	0.89	1.00	1.00	1.00	1.00	1.00	0.99	1.00	1.00	1.00
CEC17_16	0.90	0.89	0.94	0.85	0.88	0.87	0.76	0.94	0.93	0.95
CEC17_17	0.88	0.98	0.94	0.96	0.96	0.95	0.94	0.97	0.96	0.99
CEC17_18	0.88	1.00	1.00	0.99	1.00	1.00	0.99	1.00	1.00	1.00
CEC17_19	0.97	0.98	1.00	1.00	1.00	1.00	0.99	1.00	1.00	1.00
CEC17_20	0.82	0.82	0.89	0.77	0.78	0.73	0.71	0.87	0.89	0.90
CEC17_21	0.47	0.65	0.65	0.59	0.60	0.54	0.44	0.74	0.69	0.74
CEC17_22	0.98	0.86	0.84	0.56	0.63	0.75	0.68	0.88	0.90	0.96
CEC17_23	0.75	0.66	0.65	0.64	0.61	0.60	0.46	0.68	0.60	0.71
CEC17_24	0.48	0.78	0.77	0.64	0.73	0.72	0.55	0.80	0.70	0.81
CEC17_25	0.65	0.97	0.92	0.86	0.91	0.97	0.93	0.93	0.90	0.89
CEC17_26	0.86	0.94	0.99	0.83	0.90	0.86	0.79	0.88	0.98	0.93
CEC17_27	0.62	0.73	0.76	0.72	0.78	0.74	0.66	0.74	0.70	0.72
CEC17_28	0.96	0.91	0.91	0.87	0.88	0.90	0.87	0.90	0.90	0.90
CEC17_29	0.82	0.92	0.87	0.87	0.90	0.88	0.83	0.91	0.90	0.89
CEC17_30	0.90	1.00	1.00	0.99	1.00	1.00	0.97	1.00	1.00	1.00
CEC19_01	1.00	1.00	0.99	0.97	1.00	0.97	1.00	0.99	1.00	0.56
CEC19_02	0.79	1.00	0.97	0.89	0.95	0.80	1.00	0.99	1.00	0.54
CEC19_03	0.94	0.96	0.90	0.78	0.93	0.91	0.97	0.98	0.96	0.99
CEC19_04	0.98	0.79	0.84	0.98	0.89	0.83	0.68	0.98	0.80	1.00
CEC19_05	0.84	0.99	0.95	0.99	0.99	0.99	0.93	0.99	0.99	1.00
CEC19_06	0.93	0.68	0.58	0.82	0.86	0.63	0.39	0.95	0.97	0.97
CEC19_07	0.94	0.79	0.62	0.94	0.90	0.31	0.59	0.98	0.46	0.99
CEC19_08	0.38	0.37	0.45	0.58	0.36	0.25	0.22	0.61	0.55	0.91
CEC19_09	0.67	0.89	0.90	0.97	0.96	0.94	0.95	0.98	0.93	0.99
CEC19_10	0.13	0.05	0.04	0.04	0.03	0.04	0.06	0.82	0.04	0.91
CEC06_01	0.99	0.99	0.90	0.94	0.98	0.99	0.98	0.98	0.99	0.97
CEC06_02	1.00	0.99	0.97	0.99	0.99	0.99	0.99	0.98	0.99	0.99
CEC06_03	0.70	0.65	0.69	0.78	0.99	0.99	0.80	0.79	0.68	0.55
CEC06_04	0.97	0.95	0.91	0.85	0.93	0.81	0.83	0.93	0.91	0.97
CEC06_05	0.99	0.96	0.92	0.91	0.96	0.92	0.78	0.94	0.96	0.96
CEC06_06	0.97	0.96	0.95	0.84	0.95	0.94	0.82	0.94	0.96	0.93
CEC11_01	1.00	0.93	0.69	0.25	0.70	0.62	0.44	0.89	0.98	0.91
CEC11_02	1.00	0.57	0.45	0.40	0.48	0.56	0.45	0.34	0.61	0.65
CEC11_03	1.00	0.94	0.99	0.91	0.00	0.91	0.85	0.97	0.91	0.83
CEC11_04	1.00	0.69	0.27	0.27	0.74	0.27		1.00	0.16	0.79
CEC11_05	1.00	1.00	0.85	0.78	0.59	0.62	0.34	0.62	0.85	0.68
CEC11_06	1.00	0.00	0.95	0.72	0.00	0.96	0.77	0.98	0.99	0.98
CEC11_07	1.00	1.00	0.86	0.94	0.72	1.00	0.99	0.99	0.98	0.11
CEC11_08	1.00	0.00	0.52	0.52	0.43	0.65	0.53	0.91	0.85	0.06
CEC11_09	1.00	1.00	1.00	1.00	1.00	1.00	0.99	1.00	1.00	0.53
CEC11_10	1.00	0.89	0.87	0.85	0.95	1.00	0.99	0.99	0.96	0.51
CEC11_11	1.00	1.00	1.00	0.97	1.00	0.99	0.97	0.94	0.96	0.51
CEC11_12	1.00	1.00	1.00	1.00	1.00	1.00	1.00	1.00	0.97	0.50
CEC11_13	1.00	0.99	0.83	0.69	0.69	0.59	0.76	0.99	0.99	0.47
CEC11_14	1.00	1.00	0.97	0.96	0.97	0.99	0.95	0.99	1.00	0.99
CEC11_15	1.00	0.99	0.97	0.96	0.96	0.99	0.95	0.99	1.00	0.99
CEC11_16	1.00	0.99	0.96	0.96	0.96	0.98	0.96	0.99	0.98	0.99

**Table 3:** Algorithm complexity for PCOA and other competitors.

Function	PCOA	AVOA	PSO	DE	RW_GWO	WOA	HHO	LSHADE	GBO	EBOwithCMAR
CEC_17 (dim = 10)	1.49	0.09	5.58	1.94	1.05	0.17	31.21	6.63	1.57	45.77
CEC_17 (dim = 30)	1.57	12.20	4.31	0.93	1.60	2.60	49.30	9.02	0.55	113.91
CEC_17 (dim = 50)	157.90	6.99	9.77	2.09	1.43	6.47	70.31	32.78	12.92	214.50
CEC_17 (dim = 100)	241.34	21.63	6.37	5.93	8.11	12.55	136.15	24.19	21.44	373.97

## Data Availability

The raw data supporting the conclusions of this article will be made available by the authors on request.

## Abbreviations

The following abbreviations are used in this manuscript:

CX	Pine cone’s current position	$W_{2}$	Adaptive weight
TX	Tree’s current position	$W_{3}$	Adaptive weight
$CX^{new}$	Updated position of pine cone	$\mu_{W_{1}}$	Average value for $W_{1}$
$TX^{new}$	Updated position of pine tree	$\mu_{W_{2}}$	Average value for $W_{2}$
$X_{best}$	The best global position	$\mu_{W_{3}}$	Average value for $W_{3}$
$\vec{CX}$	The average of all pine cones’ positions	cr	Crossover rate
$\vec{TX}$	The average of all trees’ positions	$\mu_{cr}$	Average value for cr
$X_{animal}$	The position of pine cones that are carried by an animal	$N_{tree}$	Number of tree
$X_{initial}$	The initial position in quadratic programming	Radius	Shrinking radius
$T_{best}$	The top best individuals (10%)	FES	Current function evaluation
$Tpop_{all}$	The memory of PCOA to save solutions	$FES_{max}$	Maximum number of function evaluations
$w_{1}$	Adaptive weight	Levy	Random number with Levy flight distribution
$w_{d}$	Adaptive weight	$P_{1}$	Control parameter
$W_{1}$	Adaptive weight	$P_{2}$	Control parameter
$P_{cycle}$	Number of pollination cycle		

## Appendix A

**Table A1:** Characteristics of classic benchmark functions.

Function Formula	[Lb,Ub]	$G_{\mathrm{best}}$	Dimensions
$F_{1}\left(x\right)=\sum_{i=1}^{n}x_{i}^{2}$	${\left[-100,100\right]}^{n}$	0	30
$F_{2}\left(x\right)=\sum_{i=1}^{n}|x_{i}|+\prod_{i=1}^{n}\left|x_{i}\right|$	${\left[-10,10\right]}^{n}$	0	30
$F_{3}\left(x\right)=\sum_{i=1}^{n}{\left(\sum_{j=1}^{i}\left(x_{j}\right)\right)}^{2}$	${\left[-100,100\right]}^{n}$	0	30
$F_{4}\left(x\right)=\max_{i}\left\{\right|x_{i}\left|,1\leq i\leq n\right\}$	${\left[-100,100\right]}^{n}$	0	30
$F_{5}\left(x\right)=\sum_{i=1}^{n}-1\left[100.{\left(x_{i+1}-x_{i}^{2}\right)}^{2}+{\left(x_{i}-1\right)}^{2}\right]$	${\left[-30,30\right]}^{n}$	0	30
$F_{6}\left(x\right)=\sum_{i=1}^{n}{\left(\left[x_{i}+0.5\right]\right)}^{2}$	${\left[-100,100\right]}^{n}$	0	30
$F_{7}\left(x\right)=\sum_{i=1}^{n}\left(i.x_{i}^{4}+random\left[0,1\right)\right])$	${\left[-1.28,1.28\right]}^{n}$	0	30
$F_{8}\left(x\right)=\sum_{i=1}^{n}\left(-x_{i}.sin\sqrt{|X_{i}|}\right)$	${\left[-500,500\right]}^{n}$	428.9829.n	30
$F_{9}\left(x\right)=\sum_{i=1}^{n}\left[x_{i}^{2}-10.cos\left(2.\pi .x_{i}\right)+10\right]$	${\left[-5.12,5.12\right]}^{n}$	0	30
$F_{10}\left(x\right)=-20.exp\left(-0.2.\sqrt{\frac{1}{n}\sum_{i=1}^{n}\left(x_{i}^{2}\right)}\right)-exp\left(\frac{1}{n}\sum_{i=1}^{n}cos\left(2.\pi .x_{i}\right)\right)+20$	${\left[-32,32\right]}^{n}$	0	30
$F_{11}\left(x\right)=\frac{1}{4000}.\sum_{i=1}^{n}x_{i}^{2}-\prod_{i=1}^{n}cos\left(\frac{x_{i}}{\sqrt{i}}\right)+1$	${\left[-600,600\right]}^{n}$	0	30
$F_{12}\left(x\right)=\frac{\pi }{n}\left\{10.sin\left(\pi .y_{1}\right)+\sum_{i=1}^{n-1}{\left(y_{i}-1\right)}^{2}\left[1+10.sin^{2}\left(\pi .y_{i+1}\right)\right]+{\left(y_{n}-1\right)}^{2}\right\}+\sum_{i=1}^{n}u\left(x_{i},10,100,4\right)$	${\left[-50,50\right]}^{n}$	0	30
$y_{i}=1+\frac{x_{i+4}u\left(x_{i},10,100,4\right)}{4}=\left\{\begin{array}{cc} k.{\left(x_{i}-a\right)}^{m} & x_{i}>a\\ 0 & 0<x_{i}<a\\ k.{\left(-x_{i}-a\right)}^{m} & x_{i}<-a \end{array}\right.$			
$F_{13}\left(x\right)=0.1.\left\{sin^{2}\left(3.\pi .x_{1}\right)+\sum_{i=1}^{n}{\left(x_{i}-1\right)}^{2}\left[1+sin^{2}\left(3.\pi .x_{i}+1\right)\right]+{\left(x_{n}-1\right)}^{2}.\left[1+sin^{2}\left(2.\pi .x_{n}\right)\right]\right\}+\sum_{i=1}^{n}u\left(x_{i},5,100,4\right)$	${\left[-50,50\right]}^{n}$	0	30
$F_{14}\left(x\right)={\left(\frac{1}{500}+\sum_{j=1}^{25}\frac{1}{j+{\left(x_{i}-a_{ij}\right)}^{6}}\right)}^{-1}$	${\left[-65.53,65.53\right]}^{2}$	1	2
$F_{15}\left(x\right)=\sum_{i=1}^{11}{\left[a_{i}-\frac{x_{1}.\left(b_{i}^{2}+b_{i}.x_{2}\right)}{b_{i}^{2}+b_{i}.x_{3}+x_{4}}\right]}^{2}$	${\left[-5,5\right]}^{4}$	0.00030	4
$F_{16}\left(x\right)=4.x_{1}^{2}-2.1.x_{1}^{4}+\frac{1}{3}.x_{1}^{6}+x_{1}.x_{2}-4.x_{2}^{2}+4.x_{2}^{4}$	${\left[-5,5\right]}^{2}$	−1.0316	2
$F_{17}\left(x\right)={\left(x_{2}-\frac{-5.1}{4.\pi^{2}}.x_{1}^{2}+\frac{5}{\pi }.x_{1}-6\right)}^{2}+10.\left(1-\frac{1}{8.\pi }\right).cos\left(x_{1}\right)+10$	${\left[-5,5\right]}^{2}$	0.398	2
$F_{18}\left(x\right)=\left[1+{\left(x_{1}+x_{2}+1\right)}^{2}.\left(19-14.x_{1}+3.x_{1}^{2}-14.x_{2}+6.x_{1}.x_{2}+3.x_{2}^{2}\right)\right].\left[30+{\left(2.x_{1}-3.x_{2}\right)}^{2}.\left(18-32.x_{1}+12.x_{1}^{2}+48.x_{2}-36.x_{1}.x_{2}+27x_{2}^{2}\right)\right]$	${\left[-2,2\right]}^{2}$	3	2
$F_{19}\left(x\right)=-\sum_{i=1}^{4}c_{i}.exp\left(-\sum_{j=1}^{3}b_{ij}.\left(x_{j}-p_{ij}\right)\right)$	${\left[1,3\right]}^{3}$	−3.86	3
$F_{20}\left(x\right)=-\sum_{i=1}^{4}c_{i}.exp{\left(-\sum_{j=1}^{6}b_{ij}.\left(x_{j}-p_{ij}\right)\right)}^{2}$	${\left[0,1\right]}^{6}$	−3.32	6
$F_{21}\left(x\right)=-\sum_{i=1}^{5}{\left[\left(X-b_{i}\right).{\left(X-b_{i}\right)}^{T}+c_{i}\right]}^{-1}$	${\left[0,10\right]}^{n}$	−10.1532	4
$F_{22}\left(x\right)=-\sum_{i=1}^{7}{\left[\left(X-b_{i}\right).{\left(X-b_{i}\right)}^{T}+c_{i}\right]}^{-1}$	${\left[0,10\right]}^{n}$	−10.4028	4
$F_{23}\left(x\right)=-\sum_{i=1}^{10}{\left[\left(X-b_{i}\right).{\left(X-b_{i}\right)}^{T}+c_{i}\right]}^{-1}$	${\left[0,10\right]}^{n}$	−10.5363	4

**Table A2:** Description of CEC2019 benchmark functions.

Name	Benchmark Function Name	Dimensions	[Lb,Ub]	$G_{\mathrm{best}}$
CEC19_01	STORN’S CHEBYSHEV POLYNOMIAL FITTING PROBLEM	9	[−8,192,8,192]	1
CEC19_02	INVERSE HILBERT MATRIX PROBLEM	16	[−16,384, 16,384]	1
CEC19_03	LENNARD-JONES MINIMUM ENERGY CLUSTER	18	[−4,4]	1
CEC19_04	RASTRIGIN’S FUNCTION	10	[−100,100]	1
CEC19_05	GRIEWANGK’S FUNCTION	10	[−100,100]	1
CEC19_06	WEIERSTRASS FUNCTION	10	[−100,100]	1
CEC19_07	MODIFIED SCHWEFEL’S FUNCTION	10	[−100,100]	1
CEC19_08	EXPANDED SCHAFFER’S CEC06 FUNCTION	10	[−100,100]	1
CEC19_09	HAPPY CAT FUNCTION	10	[−100,100]	1
CEC19_10	ACKLEY FUNCTION	10	[−100,100]	1

**Table A3:** Statistical criteria results of PCOA and other algorithms on classic benchmark functions, over 51 independent runs.

Function		PCOA	AVOA	PSO	DE	RW_GWO	WOA	HHO	LSHADE	GBO	EBOwithCMAR
	Min	0.00	0.00	$3.76\times 10^{-24}$	$4.92\times 10^{-12}$	$6.74\times 10^{-97}$	$1.04\times 10^{-216}$	$3.70\times 10^{-231}$	$5.49\times 10^{-76}$	$1.11\times 10^{-306}$	0.00
	Mean	0.00	0.00	$3.89\times 10^{-21}$	$8.46\times 10^{-12}$	$4.75\times 10^{-94}$	$5.56\times 10^{-199}$	$1.31\times 10^{-207}$	$1.25\times 10^{-73}$	$1.66\times 10^{-298}$	$8.88\times 10^{-65}$
F1	Median	0.00	0.00	$1.15\times 10^{-21}$	$7.94\times 10^{-12}$	$7.48\times 10^{-95}$	$1.17\times 10^{-206}$	$1.21\times 10^{-217}$	$3.86\times 10^{-74}$	$5.89\times 10^{-302}$	$1.96\times 10^{-66}$
	Max	0.00	0.00	$4.84\times 10^{-20}$	$1.37\times 10^{-11}$	$4.52\times 10^{-93}$	$2.62\times 10^{-197}$	$6.06\times 10^{-206}$	$2.78\times 10^{-72}$	$7.10\times 10^{-297}$	$3.65\times 10^{-63}$
	Std	0.00	0.00	$7.92\times 10^{-21}$	$2.21\times 10^{-12}$	$9.74\times 10^{-94}$	0.00	0.00	$3.96\times 10^{-73}$	0.00	$5.10\times 10^{-64}$
	Min	0.00	0.00	$1.66\times 10^{-15}$	$4.87\times 10^{-08}$	$3.34\times 10^{-55}$	$4.60\times 10^{-125}$	$8.52\times 10^{-125}$	$6.54\times 10^{-37}$	$6.57\times 10^{-156}$	0.00
	Mean	0.00	0.00	$6.04\times 10^{-14}$	$6.92\times 10^{-08}$	$5.90\times 10^{-54}$	$3.74\times 10^{-115}$	$2.17\times 10^{-110}$	$1.82\times 10^{-35}$	$5.41\times 10^{-151}$	$1.02\times 10^{-31}$
F2	Median	0.00	0.00	$3.06\times 10^{-14}$	$6.74\times 10^{-08}$	$4.19\times 10^{-54}$	$1.08\times 10^{-118}$	$6.66\times 10^{-115}$	$1.21\times 10^{-35}$	$2.69\times 10^{-153}$	$9.14\times 10^{-34}$
	Max	0.00	0.00	$3.44\times 10^{-13}$	$9.95\times 10^{-08}$	$2.43\times 10^{-53}$	$1.40\times 10^{-113}$	$6.65\times 10^{-109}$	$8.67\times 10^{-35}$	$2.63\times 10^{-149}$	$4.33\times 10^{-30}$
	Std	0.00	0.00	$8.42\times 10^{-14}$	$1.19\times 10^{-08}$	$5.50\times 10^{-54}$	$2.02\times 10^{-114}$	$1.04\times 10^{-109}$	$1.80\times 10^{-35}$	$3.68\times 10^{-150}$	$6.11\times 10^{-31}$
	Min	0.00	0.00	$2.75\times 10^{0}$	$1.28\times 10^{4}$	$1.59\times 10^{-36}$	$4.47\times 10^{1}$	$7.80\times 10^{-209}$	$7.35\times 10^{-28}$	$4.39\times 10^{-255}$	$9.62\times 10^{-18}$
	Mean	0.00	0.00	$1.21\times 10^{1}$	$2.12\times 10^{4}$	$2.31\times 10^{-30}$	$2.50\times 10^{3}$	$3.91\times 10^{-189}$	$2.50\times 10^{-22}$	$1.55\times 10^{-247}$	$1.49\times 10^{-15}$
F3	Median	0.00	0.00	$1.11\times 10^{1}$	$2.19\times 10^{4}$	$5.91\times 10^{-33}$	$1.69\times 10^{3}$	$1.50\times 10^{-199}$	$7.59\times 10^{-24}$	$1.17\times 10^{-250}$	$1.68\times 10^{-17}$
	Max	0.00	0.00	$3.41\times 10^{1}$	$2.82\times 10^{4}$	$8.70\times 10^{-29}$	$8.39\times 10^{3}$	$1.16\times 10^{-187}$	$5.41\times 10^{-21}$	$7.30\times 10^{-246}$	$2.55\times 10^{-14}$
	Std	0.00	0.00	$7.30\times 10^{0}$	$3.73\times 10^{3}$	$1.22\times 10^{-29}$	$2.26\times 10^{3}$	0.00	$8.52\times 10^{-22}$	0.00	$4.29\times 10^{-15}$
	Min	0.00	0.00	$2.21\times 10^{-1}$	$1.55\times 10^{0}$	$3.78\times 10^{-26}$	$2.73\times 10^{-15}$	$2.84\times 10^{-118}$	$2.65\times 10^{-20}$	$1.85\times 10^{-142}$	$5.00\times 10^{-13}$
	Mean	0.00	0.00	$4.63\times 10^{-1}$	$2.02\times 10^{0}$	$3.28\times 10^{-24}$	$1.15\times 10^{1}$	$3.24\times 10^{-104}$	$1.77\times 10^{-18}$	$4.71\times 10^{-139}$	$1.55\times 10^{-11}$
F4	Median	0.00	0.00	$4.30\times 10^{-1}$	$2.02\times 10^{0}$	$1.95\times 10^{-24}$	$1.96\times 10^{-1}$	$4.04\times 10^{-109}$	$6.34\times 10^{-19}$	$1.10\times 10^{-139}$	$8.55\times 10^{-12}$
	Max	0.00	0.00	$1.17\times 10^{0}$	$2.4\times 10^{0}$	$1.43\times 10^{-23}$	$8.26\times 10^{1}$	$1.39\times 10^{-102}$	$2.66\times 10^{-17}$	$3.49\times 10^{-138}$	$2.31\times 10^{-10}$
	Std	0.00	0.00	$2.11\times 10^{-1}$	$2.35\times 10^{-1}$	$3.34\times 10^{-24}$	$2.20\times 10^{1}$	$1.95\times 10^{-103}$	$3.84\times 10^{-18}$	$8.34\times 10^{-139}$	$3.34\times 10^{-11}$
	Min	$0.00\times 10^{0}$	$8.57\times 10^{-9}$	$4.28\times 10^{0}$	$2.65\times 10^{1}$	$2.46\times 10^{1}$	$1.61\times 10^{-2}$	$1.39\times 10^{-11}$	$1.01\times 10^{-24}$	$1.79\times 10^{-2}$	$0.00\times 10^{0}$
	Mean	$0.00\times 10^{0}$	$4.13\times 10^{-7}$	$3.98\times 10^{1}$	$3.58\times 10^{1}$	$2.61\times 10^{1}$	$2.50\times 10^{1}$	$1.29\times 10^{-4}$	$1.56\times 10^{-1}$	$3.55\times 10^{0}$	$0.00\times 10^{0}$
F5	Median	$0.00\times 10^{0}$	$1.48\times 10^{-7}$	$2.49\times 10^{1}$	$3.39\times 10^{1}$	$2.62\times 10^{1}$	$2.55\times 10^{1}$	$5.68\times 10^{-5}$	$1.06\times 10^{-20}$	$3.28\times 10^{0}$	$0.00\times 10^{0}$
	Max	$0.00\times 10^{0}$	$6.96\times 10^{-6}$	$1.34\times 10^{2}$	$6.08\times 10^{1}$	$2.71\times 10^{1}$	$2.60\times 10^{1}$	$7.88\times 10^{-4}$	$3.99\times 10^{0}$	$9.19\times 10^{0}$	$0.00\times 10^{0}$
	Std	$0.00\times 10^{0}$	$1.02\times 10^{-6}$	$2.98\times 10^{1}$	$7.33\times 10^{0}$	$7.38\times 10^{-1}$	$3.58\times 10^{0}$	$1.80\times 10^{-4}$	$7.82\times 10^{-1}$	$2.60\times 10^{0}$	$0.00\times 10^{0}$
	Min	$0.00\times 10^{0}$	$6.68\times 10^{-14}$	$3.30\times 10^{-25}$	$4.13\times 10^{-12}$	$3.66\times 10^{-6}$	$2.28\times 10^{-5}$	$1.78\times 10^{-9}$	$0.00\times 10^{0}$	$0.00\times 10^{0}$	$0.00\times 10^{0}$
	Mean	$0.00\times 10^{0}$	$9.20\times 10^{-13}$	$6.58\times 10^{-21}$	$8.10\times 10^{-12}$	$1.20\times 10^{-1}$	$8.05\times 10^{-5}$	$1.03\times 10^{-6}$	$0.00\times 10^{0}$	$8.86\times 10^{-31}$	$0.00\times 10^{0}$
F6	Median	$0.00\times 10^{0}$	$6.68\times 10^{-13}$	$9.35\times 10^{-22}$	$7.78\times 10^{-12}$	$7.06\times 10^{-6}$	$7.45\times 10^{-5}$	$8.20\times 10^{-7}$	$0.00\times 10^{0}$	$1.97\times 10^{-31}$	$0.00\times 10^{0}$
	Max	$0.00\times 10^{0}$	$4.89\times 10^{-12}$	$7.05\times 10^{-20}$	$1.33\times 10^{-11}$	$6.58\times 10^{-1}$	$1.75\times 10^{-4}$	$4.06\times 10^{-6}$	$0.00\times 10^{0}$	$1.61\times 10^{-29}$	$0.00\times 10^{0}$
	Std	$0.00\times 10^{0}$	$7.91\times 10^{-13}$	$1.32\times 10^{-20}$	$2.19\times 10^{-12}$	$1.76\times 10^{-1}$	$3.53\times 10^{-5}$	$1.00\times 10^{-6}$	$0.00\times 10^{0}$	$2.45\times 10^{-30}$	$0.00\times 10^{0}$
	Min	$3.34\times 10^{-5}$	$7.11\times 10^{-7}$	$2.87\times 10^{-3}$	$1.29\times 10^{-2}$	$3.50\times 10^{-5}$	$1.50\times 10^{-5}$	$5.79\times 10^{-7}$	$4.33\times 10^{-4}$	$1.37\times 10^{-5}$	$2.75\times 10^{-4}$
	Mean	$3.66\times 10^{-4}$	$1.92\times 10^{-5}$	$6.13\times 10^{-3}$	$2.13\times 10^{-2}$	$1.76\times 10^{-4}$	$5.71\times 10^{-4}$	$1.81\times 10^{-5}$	$1.27\times 10^{-3}$	$1.18\times 10^{-4}$	$1.07\times 10^{-3}$
F7	Median	$3.82\times 10^{-4}$	$1.63\times 10^{-5}$	$5.74\times 10^{-3}$	$2.04\times 10^{-2}$	$1.52\times 10^{-4}$	$3.12\times 10^{-4}$	$1.21\times 10^{-5}$	$1.20\times 10^{-3}$	$1.01\times 10^{-4}$	$9.55\times 10^{-4}$
	Max	$8.40\times 10^{-4}$	$9.81\times 10^{-5}$	$9.76\times 10^{-3}$	$3.34\times 10^{-2}$	$4.53\times 10^{-4}$	$2.88\times 10^{-3}$	$1.65\times 10^{-4}$	$2.40\times 10^{-3}$	$4.37\times 10^{-4}$	$2.38\times 10^{-3}$
	Std	$2.14\times 10^{-4}$	$1.73\times 10^{-5}$	$1.87\times 10^{-3}$	$4.29\times 10^{-3}$	$1.04\times 10^{-4}$	$6.53\times 10^{-4}$	$2.63\times 10^{-5}$	$4.40\times 10^{-4}$	$8.10\times 10^{-5}$	$4.75\times 10^{-4}$
	Min	$-1.53\times 10^{4}$	$-1.26\times 10^{4}$	$-1.66\times 10^{3}$	$-1.26\times 10^{4}$	$-1.18\times 10^{6}$	$-1.26\times 10^{4}$	$-1.26\times 10^{4}$	$-1.26\times 10^{4}$	$-1.22\times 10^{4}$	$-1.55\times 10^{3}$
	Mean	$-1.38\times 10^{4}$	$-1.25\times 10^{4}$	$-1.04\times 10^{3}$	$-1.23\times 10^{4}$	$-1.01\times 10^{5}$	$-1.19\times 10^{4}$	$-1.26\times 10^{4}$	$-1.26\times 10^{4}$	$-1.56\times 10^{3}$	$-1.26\times 10^{3}$
F8	Median	$-1.38\times 10^{4}$	$-1.26\times 10^{4}$	$-1.12\times 10^{3}$	$-1.24\times 10^{4}$	$-1.98\times 10^{4}$	$-1.24\times 10^{4}$	$-1.26\times 10^{4}$	$-1.26\times 10^{4}$	$-1.39\times 10^{3}$	$-1.22\times 10^{3}$
	Max	$-1.26\times 10^{4}$	$-1.16\times 10^{4}$	$-1.74\times 10^{3}$	$-1.15\times 10^{4}$	$-1.53\times 10^{4}$	$-1.21\times 10^{3}$	$-1.26\times 10^{4}$	$-1.25\times 10^{4}$	$-1.28\times 10^{3}$	$-1.84\times 10^{3}$
	Std	$6.37\times 10^{2}$	$1.55\times 10^{2}$	$7.12\times 10^{2}$	$2.78\times 10^{2}$	$3.03\times 10^{5}$	$1.08\times 10^{3}$	$8.86\times 10^{-3}$	$1.66\times 10^{1}$	$7.94\times 10^{2}$	$7.70\times 10^{2}$
	Min	$0.00\times 10^{0}$	$0.00\times 10^{0}$	$1.99\times 10^{1}$	$4.62\times 10^{1}$	$0.00\times 10^{0}$	$0.00\times 10^{0}$	$0.00\times 10^{0}$	$0.00\times 10^{0}$	$0.00\times 10^{0}$	$-1.90\times 10^{2}$
	Mean	$0.00\times 10^{0}$	$0.00\times 10^{0}$	$3.86\times 10^{1}$	$5.88\times 10^{1}$	$0.00\times 10^{0}$	$1.11\times 10^{-15}$	$0.00\times 10^{0}$	$3.34\times 10^{-15}$	$0.00\times 10^{0}$	$-1.90\times 10^{2}$
F9	Median	$0.00\times 10^{0}$	$0.00\times 10^{0}$	$3.58\times 10^{1}$	$5.94\times 10^{1}$	$0.00\times 10^{0}$	$0.00\times 10^{0}$	$0.00\times 10^{0}$	$0.00\times 10^{0}$	$0.00\times 10^{0}$	$-1.90\times 10^{2}$
	Max	$0.00\times 10^{0}$	$0.00\times 10^{0}$	$8.66\times 10^{1}$	$6.99\times 10^{1}$	$0.00\times 10^{0}$	$5.68\times 10^{-14}$	$0.00\times 10^{0}$	$1.14\times 10^{-13}$	$0.00\times 10^{0}$	$-1.90\times 10^{2}$
	Std	$0.00\times 10^{0}$	$0.00\times 10^{0}$	$1.17\times 10^{1}$	$4.91\times 10^{0}$	$0.00\times 10^{0}$	$7.96\times 10^{-15}$	$0.00\times 10^{0}$	$1.77\times 10^{-14}$	$0.00\times 10^{0}$	$1.23\times 10^{-10}$
	Min	$8.88\times 10^{-16}$	$8.88\times 10^{-16}$	$2.00\times 10^{-13}$	$5.12\times 10^{-7}$	$7.99\times 10^{-15}$	$8.88\times 10^{-16}$	$8.88\times 10^{-16}$	$4.44\times 10^{-15}$	$8.88\times 10^{-16}$	$-1.07\times 10^{13}$
	Mean	$8.88\times 10^{-16}$	$8.88\times 10^{-16}$	$8.25\times 10^{-12}$	$7.67\times 10^{-7}$	$9.60\times 10^{-15}$	$3.60\times 10^{-15}$	$8.88\times 10^{-16}$	$5.69\times 10^{-15}$	$8.88\times 10^{-16}$	$-1.07\times 10^{13}$
F10	Median	$8.88\times 10^{-16}$	$8.88\times 10^{-16}$	$4.92\times 10^{-12}$	$7.64\times 10^{-7}$	$7.99\times 10^{-15}$	$4.44\times 10^{-15}$	$8.88\times 10^{-16}$	$4.44\times 10^{-15}$	$8.88\times 10^{-16}$	$-1.07\times 10^{13}$
	Max	$8.88\times 10^{-16}$	$8.88\times 10^{-16}$	$3.54\times 10^{-11}$	$1.01\times 10^{-6}$	$1.51\times 10^{-14}$	$7.99\times 10^{-15}$	$8.88\times 10^{-16}$	$7.99\times 10^{-15}$	$8.88\times 10^{-16}$	$-1.07\times 10^{13}$
	Std	$0.00\times 10^{0}$	$0.00\times 10^{0}$	$8.34\times 10^{-12}$	$9.95\times 10^{-8}$	$2.69\times 10^{-15}$	$2.42\times 10^{-15}$	$0.00\times 10^{0}$	$1.71\times 10^{-15}$	$0.00\times 10^{0}$	$1.97\times 10^{-3}$
	Min	$0.00\times 10^{0}$	$0.00\times 10^{0}$	$0.00\times 10^{0}$	$1.77\times 10^{-11}$	$0.00\times 10^{0}$	$0.00\times 10^{0}$	$0.00\times 10^{0}$	$0.00\times 10^{0}$	$0.00\times 10^{0}$	$0.00\times 10^{0}$
	Mean	$0.00\times 10^{0}$	$0.00\times 10^{0}$	$1.36\times 10^{-2}$	$7.01\times 10^{-11}$	$1.07\times 10^{-3}$	$1.70\times 10^{-3}$	$0.00\times 10^{0}$	$0.00\times 10^{0}$	$0.00\times 10^{0}$	$9.67\times 10^{-5}$
F11	Median	$0.00\times 10^{0}$	$0.00\times 10^{0}$	$9.86\times 10^{-3}$	$6.09\times 10^{-11}$	$0.00\times 10^{0}$	$0.00\times 10^{0}$	$0.00\times 10^{0}$	$0.00\times 10^{0}$	$0.00\times 10^{0}$	$0.00\times 10^{0}$
	Max	$0.00\times 10^{0}$	$0.00\times 10^{0}$	$7.12\times 10^{-2}$	$2.07\times 10^{-10}$	$3.46\times 10^{-2}$	$2.44\times 10^{-2}$	$0.00\times 10^{0}$	$0.00\times 10^{0}$	$0.00\times 10^{0}$	$4.93\times 10^{-3}$
	Std	$0.00\times 10^{0}$	$0.00\times 10^{0}$	$1.47\times 10^{-2}$	$4.10\times 10^{-11}$	$5.18\times 10^{-3}$	$5.49\times 10^{-3}$	$0.00\times 10^{0}$	$0.00\times 10^{0}$	$0.00\times 10^{0}$	$6.91\times 10^{-4}$
	Min	$1.57\times 10^{-32}$	$1.33\times 10^{-14}$	$2.34\times 10^{-26}$	$6.27\times 10^{-13}$	$2.09\times 10^{-7}$	$4.69\times 10^{-6}$	$1.30\times 10^{-10}$	$1.57\times 10^{-32}$	$1.57\times 10^{-32}$	$1.57\times 10^{-32}$
	Mean	$1.57\times 10^{-32}$	$7.99\times 10^{-14}$	$7.13\times 10^{-22}$	$1.05\times 10^{-12}$	$8.90\times 10^{-3}$	$1.28\times 10^{-5}$	$1.47\times 10^{-7}$	$1.57\times 10^{-32}$	$3.77\times 10^{-31}$	$1.57\times 10^{-32}$
F12	Median	$1.57\times 10^{-32}$	$7.08\times 10^{-14}$	$4.53\times 10^{-24}$	$1.03\times 10^{-12}$	$6.58\times 10^{-3}$	$1.20\times 10^{-5}$	$5.30\times 10^{-8}$	$1.57\times 10^{-32}$	$2.86\times 10^{-32}$	$1.57\times 10^{-32}$
	Max	$1.57\times 10^{-32}$	$2.20\times 10^{-13}$	$3.21\times 10^{-20}$	$2.00\times 10^{-12}$	$3.34\times 10^{-2}$	$2.89\times 10^{-5}$	$1.32\times 10^{-6}$	$1.57\times 10^{-32}$	$1.32\times 10^{-29}$	$1.57\times 10^{-32}$
	Std	$2.76\times 10^{-48}$	$4.62\times 10^{-14}$	$4.50\times 10^{-21}$	$2.95\times 10^{-13}$	$8.04\times 10^{-3}$	$5.53\times 10^{-6}$	$2.63\times 10^{-7}$	$2.76\times 10^{-48}$	$1.86\times 10^{-30}$	$2.76\times 10^{-48}$
	Min	$1.35\times 10^{-32}$	$1.75\times 10^{-12}$	$1.53\times 10^{-25}$	$2.16\times 10^{-12}$	$2.88\times 10^{-6}$	$6.56\times 10^{-5}$	$1.17\times 10^{-9}$	$1.35\times 10^{-32}$	$1.35\times 10^{-32}$	$1.35\times 10^{-32}$
	Mean	$1.35\times 10^{-32}$	$1.10\times 10^{-11}$	$8.62\times 10^{-4}$	$5.14\times 10^{-12}$	$1.12\times 10^{-1}$	$1.95\times 10^{-3}$	$1.35\times 10^{-6}$	$1.35\times 10^{-32}$	$7.50\times 10^{-3}$	$1.35\times 10^{-32}$
F13	Median	$1.35\times 10^{-32}$	$1.08\times 10^{-11}$	$2.38\times 10^{-22}$	$4.72\times 10^{-12}$	$1.00\times 10^{-1}$	$2.13\times 10^{-4}$	$5.19\times 10^{-7}$	$1.35\times 10^{-32}$	$9.87\times 10^{-30}$	$1.35\times 10^{-32}$
	Max	$1.35\times 10^{-32}$	$2.78\times 10^{-11}$	$1.10\times 10^{-2}$	$8.83\times 10^{-12}$	$4.14\times 10^{-1}$	$1.13\times 10^{-2}$	$9.86\times 10^{-6}$	$1.35\times 10^{-32}$	$4.39\times 10^{-2}$	$1.35\times 10^{-32}$
	Std	$2.76\times 10^{-48}$	$7.23\times 10^{-12}$	$2.98\times 10^{-3}$	$1.56\times 10^{-12}$	$1.03\times 10^{-1}$	$4.01\times 10^{-3}$	$1.91\times 10^{-6}$	$2.76\times 10^{-48}$	$1.10\times 10^{-2}$	$2.76\times 10^{-48}$
	Min	$9.98\times 10^{-1}$	$9.98\times 10^{-1}$	$9.98\times 10^{-1}$	$9.98\times 10^{-1}$	$9.98\times 10^{-1}$	$9.98\times 10^{-1}$	$9.98\times 10^{-1}$	$9.98\times 10^{-1}$	$9.98\times 10^{-1}$	$9.98\times 10^{-1}$
	Mean	$9.98\times 10^{-1}$	$9.98\times 10^{-1}$	$9.98\times 10^{-1}$	$9.98\times 10^{-1}$	$1.02\times 10^{0}$	$1.04\times 10^{0}$	$9.98\times 10^{-1}$	$9.98\times 10^{-1}$	$9.98\times 10^{-1}$	$9.98\times 10^{-1}$
F14	Median	$9.98\times 10^{-1}$	$9.98\times 10^{-1}$	$9.98\times 10^{-1}$	$9.98\times 10^{-1}$	$9.98\times 10^{-1}$	$9.98\times 10^{-1}$	$9.98\times 10^{-1}$	$9.98\times 10^{-1}$	$9.98\times 10^{-1}$	$9.98\times 10^{-1}$
	Max	$9.98\times 10^{-1}$	$9.98\times 10^{-1}$	$9.98\times 10^{-1}$	$9.98\times 10^{-1}$	$1.99\times 10^{0}$	$2.98\times 10^{0}$	$9.98\times 10^{-1}$	$9.98\times 10^{-1}$	$9.98\times 10^{-1}$	$9.98\times 10^{-1}$
	Std	$0.00\times 10^{0}$	$2.11\times 10^{-16}$	$0.00\times 10^{0}$	$0.00\times 10^{0}$	$1.39\times 10^{-1}$	$2.78\times 10^{-1}$	$1.60\times 10^{-14}$	$0.00\times 10^{0}$	$0.00\times 10^{0}$	$0.00\times 10^{0}$
	Min	$3.07\times 10^{-4}$	$3.07\times 10^{-4}$	$3.07\times 10^{-4}$	$3.52\times 10^{-4}$	$3.07\times 10^{-4}$	$3.08\times 10^{-4}$	$3.07\times 10^{-4}$	$3.07\times 10^{-4}$	$3.07\times 10^{-4}$	$3.07\times 10^{-4}$
	Mean	$3.07\times 10^{-4}$	$3.07\times 10^{-4}$	$4.17\times 10^{-4}$	$6.00\times 10^{-4}$	$3.76\times 10^{-4}$	$5.26\times 10^{-4}$	$3.12\times 10^{-4}$	$3.07\times 10^{-4}$	$4.40\times 10^{-4}$	$3.07\times 10^{-4}$
F15	Median	$3.07\times 10^{-4}$	$3.07\times 10^{-4}$	$3.07\times 10^{-4}$	$6.04\times 10^{-4}$	$3.07\times 10^{-4}$	$3.55\times 10^{-4}$	$3.09\times 10^{-4}$	$3.07\times 10^{-4}$	$3.07\times 10^{-4}$	$3.07\times 10^{-4}$
	Max	$3.07\times 10^{-4}$	$3.07\times 10^{-4}$	$1.30\times 10^{-3}$	$7.46\times 10^{-4}$	$1.80\times 10^{-3}$	$1.23\times 10^{-3}$	$4.26\times 10^{-4}$	$3.07\times 10^{-4}$	$1.59\times 10^{-3}$	$3.07\times 10^{-4}$
	Std	$1.18\times 10^{-19}$	$6.87\times 10^{-10}$	$3.02\times 10^{-4}$	$8.53\times 10^{-5}$	$2.84\times 10^{-4}$	$3.27\times 10^{-4}$	$1.66\times 10^{-5}$	$9.14\times 10^{-20}$	$3.40\times 10^{-4}$	$1.03\times 10^{-19}$
	Min	$-1.03\times 10^{0}$	$-1.03\times 10^{0}$	$-1.03\times 10^{0}$	$-1.03\times 10^{0}$	$-1.03\times 10^{0}$	$-1.03\times 10^{0}$	$-1.03\times 10^{0}$	$-1.03\times 10^{0}$	$-1.03\times 10^{0}$	$-1.03\times 10^{0}$
	Mean	$-1.03\times 10^{0}$	$-1.03\times 10^{0}$	$-1.03\times 10^{0}$	$-1.03\times 10^{0}$	$-1.03\times 10^{0}$	$-1.03\times 10^{0}$	$-1.03\times 10^{0}$	$-1.03\times 10^{0}$	$-1.03\times 10^{0}$	$-1.03\times 10^{0}$
F16	Median	$-1.03\times 10^{0}$	$-1.03\times 10^{0}$	$-1.03\times 10^{0}$	$-1.03\times 10^{0}$	$-1.03\times 10^{0}$	$-1.03\times 10^{0}$	$-1.03\times 10^{0}$	$-1.03\times 10^{0}$	$-1.03\times 10^{0}$	$-1.03\times 10^{0}$
	Max	$-1.03\times 10^{0}$	$-1.03\times 10^{0}$	$-1.03\times 10^{0}$	$-1.03\times 10^{0}$	$-1.03\times 10^{0}$	$-1.03\times 10^{0}$	$-1.03\times 10^{0}$	$-1.03\times 10^{0}$	$-1.03\times 10^{0}$	$-1.03\times 10^{0}$
	Std	$0.00\times 10^{0}$	$1.94\times 10^{-16}$	$0.00\times 10^{0}$	$0.00\times 10^{0}$	$1.16\times 10^{-9}$	$1.07\times 10^{-13}$	$7.56\times 10^{-16}$	$0.00\times 10^{0}$	$0.00\times 10^{0}$	$0.00\times 10^{0}$
	Min	$3.98\times 10^{-1}$	$3.98\times 10^{-1}$	$3.98\times 10^{-1}$	$3.98\times 10^{-1}$	$3.98\times 10^{-1}$	$3.98\times 10^{-1}$	$3.98\times 10^{-1}$	$3.98\times 10^{-1}$	$3.98\times 10^{-1}$	$3.98\times 10^{-1}$
	Mean	$3.98\times 10^{-1}$	$3.98\times 10^{-1}$	$3.98\times 10^{-1}$	$3.98\times 10^{-1}$	$3.98\times 10^{-1}$	$3.98\times 10^{-1}$	$3.98\times 10^{-1}$	$3.98\times 10^{-1}$	$3.98\times 10^{-1}$	$3.98\times 10^{-1}$
F17	Median	$3.98\times 10^{-1}$	$3.98\times 10^{-1}$	$3.98\times 10^{-1}$	$3.98\times 10^{-1}$	$3.98\times 10^{-1}$	$3.98\times 10^{-1}$	$3.98\times 10^{-1}$	$3.98\times 10^{-1}$	$3.98\times 10^{-1}$	$3.98\times 10^{-1}$
	Max	$3.98\times 10^{-1}$	$3.98\times 10^{-1}$	$3.98\times 10^{-1}$	$3.98\times 10^{-1}$	$3.98\times 10^{-1}$	$3.98\times 10^{-1}$	$3.98\times 10^{-1}$	$3.98\times 10^{-1}$	$3.98\times 10^{-1}$	$3.98\times 10^{-1}$
	Std	$5.61\times 10^{-17}$	$5.61\times 10^{-17}$	$5.61\times 10^{-17}$	$5.61\times 10^{-17}$	$1.49\times 10^{-6}$	$1.19\times 10^{-8}$	$4.93\times 10^{-11}$	$5.61\times 10^{-17}$	$5.61\times 10^{-17}$	$5.61\times 10^{-17}$
	Min	$3.00\times 10^{0}$	$3.00\times 10^{0}$	$3.00\times 10^{0}$	$3.00\times 10^{0}$	$3.00\times 10^{0}$	$3.00\times 10^{0}$	$3.00\times 10^{0}$	$3.00\times 10^{0}$	$3.00\times 10^{0}$	$3.00\times 10^{0}$
	Mean	$3.00\times 10^{0}$	$3.00\times 10^{0}$	$3.00\times 10^{0}$	$3.00\times 10^{0}$	$3.00\times 10^{0}$	$3.00\times 10^{0}$	$3.00\times 10^{0}$	$3.00\times 10^{0}$	$3.00\times 10^{0}$	$3.00\times 10^{0}$
F18	Median	$3.00\times 10^{0}$	$3.00\times 10^{0}$	$3.00\times 10^{0}$	$3.00\times 10^{0}$	$3.00\times 10^{0}$	$3.00\times 10^{0}$	$3.00\times 10^{0}$	$3.00\times 10^{0}$	$3.00\times 10^{0}$	$3.00\times 10^{0}$
	Max	$3.00\times 10^{0}$	$3.00\times 10^{0}$	$3.00\times 10^{0}$	$3.00\times 10^{0}$	$3.00\times 10^{0}$	$3.00\times 10^{0}$	$3.00\times 10^{0}$	$3.00\times 10^{0}$	$3.00\times 10^{0}$	$3.00\times 10^{0}$
	Std	$9.55\times 10^{-16}$	$2.76\times 10^{-9}$	$6.65\times 10^{-16}$	$7.59\times 10^{-16}$	$4.01\times 10^{-7}$	$6.35\times 10^{-8}$	$3.85\times 10^{-14}$	$1.07\times 10^{-15}$	$8.72\times 10^{-16}$	$1.18\times 10^{-15}$
	Min	$-1.86\times 10^{0}$	$-1.86\times 10^{0}$	$-1.86\times 10^{0}$	$-1.86\times 10^{0}$	$-1.86\times 10^{0}$	$-1.86\times 10^{0}$	$-1.86\times 10^{0}$	$-1.86\times 10^{0}$	$-1.86\times 10^{0}$	$-1.86\times 10^{0}$
	Mean	$-1.86\times 10^{0}$	$-1.86\times 10^{0}$	$-1.86\times 10^{0}$	$-1.86\times 10^{0}$	$-1.86\times 10^{0}$	$-1.86\times 10^{0}$	$-1.86\times 10^{0}$	$-1.86\times 10^{0}$	$-1.86\times 10^{0}$	$-1.86\times 10^{0}$
F19	Median	$-1.86\times 10^{0}$	$-1.86\times 10^{0}$	$-1.86\times 10^{0}$	$-1.86\times 10^{0}$	$-1.86\times 10^{0}$	$-1.86\times 10^{0}$	$-1.86\times 10^{0}$	$-1.86\times 10^{0}$	$-1.86\times 10^{0}$	$-1.86\times 10^{0}$
	Max	$-1.86\times 10^{0}$	$-1.86\times 10^{0}$	$-1.86\times 10^{0}$	$-1.86\times 10^{0}$	$-1.85\times 10^{0}$	$-1.85\times 10^{0}$	$-1.86\times 10^{0}$	$-1.86\times 10^{0}$	$-1.86\times 10^{0}$	$-1.86\times 10^{0}$
	Std	$1.35\times 10^{-15}$	$9.50\times 10^{-16}$	$1.35\times 10^{-15}$	$1.35\times 10^{-15}$	$1.66\times 10^{-3}$	$1.49\times 10^{-3}$	$1.34\times 10^{-8}$	$1.35\times 10^{-15}$	$1.35\times 10^{-15}$	$1.35\times 10^{-15}$
	Min	$-1.32\times 10^{0}$	$-1.32\times 10^{0}$	$-1.32\times 10^{0}$	$-1.32\times 10^{0}$	$-1.32\times 10^{0}$	$-1.32\times 10^{0}$	$-1.32\times 10^{0}$	$-1.32\times 10^{0}$	$-1.32\times 10^{0}$	$-1.32\times 10^{0}$
	Mean	$-1.32\times 10^{0}$	$-1.26\times 10^{0}$	$-1.27\times 10^{0}$	$-1.32\times 10^{0}$	$-1.24\times 10^{0}$	$-1.25\times 10^{0}$	$-1.25\times 10^{0}$	$-1.32\times 10^{0}$	$-1.25\times 10^{0}$	$-1.32\times 10^{0}$
F20	Median	$-1.32\times 10^{0}$	$-1.32\times 10^{0}$	$-1.32\times 10^{0}$	$-1.32\times 10^{0}$	$-1.20\times 10^{0}$	$-1.20\times 10^{0}$	$-1.20\times 10^{0}$	$-1.32\times 10^{0}$	$-1.20\times 10^{0}$	$-1.32\times 10^{0}$
	Max	$-1.32\times 10^{0}$	$-1.20\times 10^{0}$	$-1.20\times 10^{0}$	$-1.32\times 10^{0}$	$-1.08\times 10^{0}$	$-1.14\times 10^{0}$	$-1.14\times 10^{0}$	$-1.20\times 10^{0}$	$-1.20\times 10^{0}$	$-1.32\times 10^{0}$
	Std	$5.33\times 10^{-16}$	$6.00\times 10^{-2}$	$5.99\times 10^{-2}$	$4.44\times 10^{-16}$	$7.28\times 10^{-2}$	$6.18\times 10^{-2}$	$6.62\times 10^{-2}$	$2.83\times 10^{-2}$	$5.91\times 10^{-2}$	$4.98\times 10^{-16}$
	Min	$-1.02\times 10^{1}$	$-1.02\times 10^{1}$	$-1.02\times 10^{1}$	$-1.02\times 10^{1}$	$-1.02\times 10^{1}$	$-1.02\times 10^{1}$	$-1.02\times 10^{1}$	$-1.02\times 10^{1}$	$-1.02\times 10^{1}$	$-1.02\times 10^{1}$
	Mean	$-1.02\times 10^{1}$	$-1.02\times 10^{1}$	$-1.76\times 10^{0}$	$-1.02\times 10^{1}$	$-1.58\times 10^{0}$	$-1.02\times 10^{1}$	$-1.75\times 10^{0}$	$-1.01\times 10^{1}$	$-1.35\times 10^{0}$	$-1.02\times 10^{1}$
F21	Median	$-1.02\times 10^{1}$	$-1.02\times 10^{1}$	$-1.10\times 10^{0}$	$-1.02\times 10^{1}$	$-1.02\times 10^{1}$	$-1.02\times 10^{1}$	$-1.06\times 10^{0}$	$-1.02\times 10^{1}$	$-1.02\times 10^{1}$	$-1.02\times 10^{1}$
	Max	$-1.02\times 10^{1}$	$-1.02\times 10^{1}$	$-1.63\times 10^{0}$	$-1.02\times 10^{1}$	$-1.06\times 10^{0}$	$-1.02\times 10^{1}$	$-1.06\times 10^{0}$	$-1.10\times 10^{0}$	$-1.06\times 10^{0}$	$-1.02\times 10^{1}$
	Std	$0.00\times 10^{0}$	$2.47\times 10^{-15}$	$3.24\times 10^{0}$	$2.27\times 10^{-12}$	$1.60\times 10^{0}$	$1.03\times 10^{-5}$	$1.77\times 10^{0}$	$7.07\times 10^{-1}$	$1.87\times 10^{0}$	$0.00\times 10^{0}$
	Min	$-1.04\times 10^{1}$	$-1.04\times 10^{1}$	$-1.04\times 10^{1}$	$-1.04\times 10^{1}$	$-1.04\times 10^{1}$	$-1.04\times 10^{1}$	$-1.04\times 10^{1}$	$-1.04\times 10^{1}$	$-1.04\times 10^{1}$	$-1.04\times 10^{1}$
	Mean	$-1.04\times 10^{1}$	$-1.04\times 10^{1}$	$-1.12\times 10^{0}$	$-1.04\times 10^{1}$	$-1.04\times 10^{1}$	$-1.00\times 10^{1}$	$-1.13\times 10^{0}$	$-1.04\times 10^{1}$	$-1.88\times 10^{0}$	$-1.04\times 10^{1}$
F22	Median	$-1.04\times 10^{1}$	$-1.04\times 10^{1}$	$-1.04\times 10^{1}$	$-1.04\times 10^{1}$	$-1.04\times 10^{1}$	$-1.04\times 10^{1}$	$-1.09\times 10^{0}$	$-1.04\times 10^{1}$	$-1.04\times 10^{1}$	$-1.04\times 10^{1}$
	Max	$-1.04\times 10^{1}$	$-1.04\times 10^{1}$	$-1.75\times 10^{0}$	$-1.04\times 10^{1}$	$-1.04\times 10^{1}$	$-1.77\times 10^{0}$	$-1.09\times 10^{0}$	$-1.04\times 10^{1}$	$-1.09\times 10^{0}$	$-1.04\times 10^{1}$
	Std	$1.99\times 10^{-15}$	$1.44\times 10^{-15}$	$3.20\times 10^{0}$	$1.31\times 10^{-15}$	$7.67\times 10^{-5}$	$1.47\times 10^{0}$	$2.13\times 10^{0}$	$3.55\times 10^{-16}$	$1.60\times 10^{0}$	$1.79\times 10^{-15}$
	Min	$-1.05\times 10^{1}$	$-1.05\times 10^{1}$	$-1.05\times 10^{1}$	$-1.05\times 10^{1}$	$-1.05\times 10^{1}$	$-1.05\times 10^{1}$	$-1.05\times 10^{1}$	$-1.05\times 10^{1}$	$-1.05\times 10^{1}$	$-1.05\times 10^{1}$
	Mean	$-1.05\times 10^{1}$	$-1.05\times 10^{1}$	$-1.10\times 10^{0}$	$-1.05\times 10^{1}$	$-1.03\times 10^{1}$	$-1.05\times 10^{1}$	$-1.87\times 10^{0}$	$-1.05\times 10^{1}$	$-1.69\times 10^{0}$	$-1.05\times 10^{1}$
F23	Median	$-1.05\times 10^{1}$	$-1.05\times 10^{1}$	$-1.05\times 10^{1}$	$-1.05\times 10^{1}$	$-1.05\times 10^{1}$	$-1.05\times 10^{1}$	$-1.13\times 10^{0}$	$-1.05\times 10^{1}$	$-1.05\times 10^{1}$	$-1.05\times 10^{1}$
	Max	$-1.05\times 10^{1}$	$-1.05\times 10^{1}$	$-1.42\times 10^{0}$	$-1.05\times 10^{1}$	$-1.13\times 10^{0}$	$-1.05\times 10^{1}$	$-1.13\times 10^{0}$	$-1.05\times 10^{1}$	$-1.13\times 10^{0}$	$-1.05\times 10^{1}$
	Std	$1.79\times 10^{-15}$	$1.76\times 10^{-15}$	$3.41\times 10^{0}$	$1.79\times 10^{-15}$	$1.06\times 10^{0}$	$1.80\times 10^{-3}$	$1.88\times 10^{0}$	$2.04\times 10^{-15}$	$1.99\times 10^{0}$	$2.04\times 10^{-15}$

**Table A4:** Statistical criteria results of PCOA and other algorithms on the 10D CEC2017, over 51 independent runs.

Function		PCOA	AVOA	PSO	DE	RW_GWO	WOA	HHO	LSHADE	GBO	EBOwithCMAR
	Min	100.00	102.70	100.96	135.13	2439.47	7915.27	36460.41	100.00	100.10	100.00
	Mean	100.00	4312.89	1723.10	1371.99	292277.87	295915.24	233033.39	100.00	1004.74	100.00
CEC_17/01	Median	100.00	3954.98	785.93	1230.12	11000.50	70542.61	218064.13	100.00	476.65	100.00
	Max	100.00	12739.83	12252.30	4280.86	7276035.49	2676940.69	577133.76	100.00	6084.59	100.00
	Std	0.00	3600.65	2329.07	1010.83	1269917.69	579476.71	117529.75	0.00	1235.43	0.00
	Min	300.00	300.00	300.00	879.94	300.06	310.19	300.20	300.00	300.00	300.00
	Mean	300.00	300.00	300.00	1936.66	304.15	511.89	300.78	300.00	300.00	300.00
CEC_17/03	Median	300.00	300.00	300.00	1922.24	302.75	468.70	300.73	300.00	300.00	300.00
	Max	300.00	300.00	300.00	3465.34	317.71	1241.93	301.66	300.00	300.00	300.00
	Std	0.00	0.00	0.00	564.41	4.39	187.70	0.35	0.00	0.00	0.00
	Min	400.00	400.02	400.03	404.94	400.20	400.12	400.06	400.00	400.00	400.00
	Mean	400.00	403.89	400.43	406.01	406.65	426.11	421.85	400.00	400.00	400.00
CEC_17/04	Median	400.00	404.55	400.37	406.10	407.17	407.60	407.04	400.00	400.00	400.00
	Max	400.00	406.25	401.52	406.57	407.76	550.26	500.39	400.00	400.00	400.00
	Std	0.00	1.54	0.32	0.36	1.63	38.06	30.38	0.00	0.00	0.00
	Min	500.00	506.96	508.95	505.94	506.97	517.04	513.32	501.03	503.98	500.00
	Mean	500.33	531.81	519.00	509.53	517.88	548.42	539.51	503.45	521.18	500.04
CEC_17/05	Median	500.00	531.84	517.91	509.57	516.05	545.77	539.87	503.18	520.89	500.00
	Max	503.98	557.87	544.77	513.22	530.26	584.60	567.02	505.16	543.78	500.99
	Std	0.71	11.63	7.20	1.64	6.02	17.64	14.66	1.04	8.67	0.20
	Min	600.00	600.15	600.00	600.00	600.03	606.41	601.79	600.00	600.00	600.00
	Mean	600.00	606.18	600.66	600.00	600.09	629.83	626.74	600.00	600.05	600.00
CEC_17/06	Median	600.00	602.26	600.00	600.00	600.06	629.63	625.50	600.00	600.00	600.00
	Max	600.00	627.73	608.72	600.00	600.51	660.34	654.49	600.00	601.01	600.00
	Std	0.00	7.53	1.59	0.00	0.09	12.25	12.64	0.00	0.18	0.00
	Min	710.46	719.00	707.96	714.85	713.96	724.62	725.78	710.74	716.88	710.37
	Mean	711.39	751.56	720.26	721.29	726.72	775.21	771.76	712.91	734.61	710.62
CEC_17/07	Median	711.13	749.90	719.26	721.55	725.60	772.32	775.75	712.87	733.43	710.60
	Max	716.24	790.49	736.39	724.17	747.55	849.95	811.68	714.86	767.46	711.33
	Std	0.90	15.91	5.69	1.68	8.69	25.87	19.81	0.82	10.55	0.21
	Min	800.00	806.96	804.97	806.37	803.98	811.95	808.04	801.10	806.96	800.00
	Mean	800.60	824.50	812.95	810.14	815.07	839.82	828.80	803.09	821.10	800.04
CEC_17/08	Median	800.00	821.89	812.93	810.45	814.95	839.80	828.95	803.11	820.89	800.00
	Max	803.98	852.73	824.87	814.72	831.00	872.64	853.80	805.13	837.81	800.99
	Std	0.87	10.70	4.66	1.96	5.70	14.67	10.09	0.99	6.99	0.18
	Min	900.00	900.00	900.00	900.00	900.01	955.95	936.51	900.00	900.00	900.00
	Mean	900.00	992.43	900.00	900.00	900.24	1329.27	1280.98	900.00	902.40	900.00
CEC_17/09	Median	900.00	945.72	900.00	900.00	900.15	1240.42	1251.71	900.00	900.54	900.00
	Max	900.00	1467.93	900.09	900.00	901.07	2230.91	1988.21	900.00	917.77	900.00
	Std	0.00	121.14	0.02	0.00	0.21	284.21	246.47	0.00	4.24	0.00
	Min	1000.31	1239.78	1122.10	1224.47	1006.99	1462.16	1364.00	1002.32	1033.47	1000.12
	Mean	1213.54	1807.25	1725.69	1499.66	1684.13	2035.03	1964.97	1053.54	1796.73	1040.74
CEC_17/10	Median	1148.94	1805.86	1731.51	1488.98	1684.84	2003.27	1955.32	1020.27	1758.07	1010.31
	Max	1690.47	2655.32	2288.46	1655.37	2408.16	2790.73	2509.85	1156.71	2472.82	1140.59
	Std	179.47	306.96	267.92	89.18	318.23	328.16	317.63	53.66	382.81	52.59
	Min	1100.00	1110.42	1101.00	1101.84	1102.02	1110.62	1106.76	1100.00	1100.99	1100.00
	Mean	1100.00	1129.01	1117.56	1103.23	1118.13	1213.21	1174.19	1100.26	1111.44	1100.00
CEC_17/11	Median	1100.00	1128.13	1116.64	1103.29	1115.48	1182.55	1148.59	1100.00	1109.95	1100.00
	Max	1100.00	1165.69	1136.53	1104.62	1139.40	1474.54	1438.76	1102.54	1140.51	1100.00
	Std	0.00	12.63	9.99	0.69	8.49	94.62	78.49	0.67	7.39	0.00
	Min	1200.01	2415.85	1477.84	36299.73	4111.51	5248.95	5698.61	1200.00	1756.71	1200.00
	Mean	1228.06	132474.92	11755.29	200376.87	411662.85	4206287.96	1162284.97	1254.76	9008.62	1305.65
CEC_17/12	Median	1200.24	62475.27	7636.71	194254.38	150558.47	1128022.07	693823.69	1200.42	5114.42	1318.65
	Max	1357.53	854825.32	42196.42	412382.29	2177557.30	16104189.76	5643740.41	1437.08	48238.37	1438.18
	Std	50.98	183238.78	9941.89	83886.38	609954.72	5158085.66	1369978.67	70.71	9667.51	54.85
	Min	1300.02	1614.52	1519.67	1399.24	1627.30	2001.09	1430.90	1300.00	1315.37	1300.00
	Mean	1303.74	9260.09	7000.24	2543.82	8099.66	17137.51	14957.44	1303.71	1617.12	1302.92
CEC_17/13	Median	1304.86	4196.33	6368.89	2251.27	5794.93	13227.39	11656.41	1304.84	1486.43	1304.84
	Max	1307.30	32117.88	25692.23	5957.97	20336.83	44508.74	36610.82	1307.95	4386.19	1308.37
	Std	2.37	9300.00	4786.33	1041.33	5340.20	12861.08	10314.04	2.24	481.00	2.88
	Min	1400.00	1438.49	1410.22	1402.10	1431.33	1461.81	1449.90	1400.00	1427.16	1400.00
	Mean	1401.58	1505.73	1493.21	1423.75	1951.21	1590.80	1516.01	1400.09	1479.00	1400.03
CEC_17/14	Median	1401.00	1473.64	1465.62	1415.58	1485.84	1532.57	1515.97	1400.00	1477.81	1400.00
	Max	1421.01	1940.64	2017.12	1617.52	4996.08	3587.22	1610.16	1401.08	1574.65	1401.00
	Std	3.25	97.23	96.73	31.55	1119.87	294.60	29.84	0.25	33.86	0.14
	Min	1500.00	1515.54	1510.37	1503.15	1509.98	1645.24	1552.72	1500.00	1502.51	1500.00
	Mean	1500.08	2374.80	1667.28	1534.60	2187.20	4243.55	1935.86	1500.12	1576.95	1500.19
CEC_17/15	Median	1500.04	1693.37	1591.87	1522.00	1748.71	2885.60	1754.63	1500.01	1555.35	1500.04
	Max	1500.41	4271.99	2707.72	1625.71	4619.45	13038.27	3637.64	1500.50	1795.85	1500.50
	Std	0.11	1091.57	211.38	30.78	949.00	2871.28	493.98	0.18	68.30	0.22
	Min	1600.02	1603.92	1600.61	1600.84	1603.97	1611.55	1604.36	1600.09	1600.95	1600.02
	Mean	1600.39	1761.75	1817.30	1602.59	1673.18	1806.85	1845.21	1600.55	1727.66	1600.41
CEC_17/16	Median	1600.29	1732.98	1839.48	1602.26	1624.72	1782.08	1859.80	1600.57	1723.56	1600.42
	Max	1601.60	2059.24	2019.73	1609.01	1985.85	2118.66	2105.10	1600.99	2005.07	1600.86
	Std	0.36	107.15	124.51	1.33	96.67	115.27	145.92	0.21	116.57	0.20
	Min	1700.18	1721.50	1706.33	1700.49	1718.12	1730.66	1727.06	1700.06	1702.09	1700.01
	Mean	1702.58	1764.90	1752.92	1701.89	1755.63	1809.73	1767.20	1700.42	1733.61	1700.15
CEC_17/17	Median	1701.66	1751.05	1746.15	1701.79	1745.94	1786.60	1761.39	1700.41	1733.00	1700.07
	Max	1720.49	1867.39	1864.15	1705.56	1885.46	1957.83	1856.01	1701.13	1784.32	1700.64
	Std	3.27	37.93	29.38	0.95	36.92	57.23	26.69	0.23	18.64	0.16
	Min	1800.05	2524.50	1904.43	1970.86	2542.69	2123.21	2023.05	1800.00	1844.02	1800.00
	Mean	1800.55	14537.39	8241.89	2780.07	24587.99	17612.72	14585.47	1800.98	2022.97	1800.31
CEC_17/18	Median	1800.50	10776.59	4850.99	2591.89	24762.65	15656.72	12745.97	1800.06	1937.78	1800.39
	Max	1801.54	46035.86	29748.03	4465.11	55129.22	42302.15	48567.29	1820.14	3156.85	1800.50
	Std	0.37	11349.36	7398.50	641.46	16644.94	10840.98	11808.48	3.91	255.17	0.18
	Min	1900.02	1934.90	1907.17	1900.43	1914.98	1987.16	1960.31	1900.00	1903.82	1900.00
	Mean	1900.16	6716.24	2965.90	1934.34	3633.70	19300.84	9184.00	1900.02	1945.88	1900.02
CEC_17/19	Median	1900.08	3708.05	2051.55	1913.50	1938.32	9366.37	6255.76	1900.01	1933.75	1900.02
	Max	1901.56	21532.73	10403.01	2353.13	13273.83	231816.56	28920.82	1900.07	2077.16	1900.04
	Std	0.26	5461.81	1815.59	66.95	3568.13	33453.35	7683.22	0.02	40.65	0.01
	Min	2000.00	2017.23	2002.30	2000.00	2018.47	2039.55	2038.79	2000.00	2000.31	2000.00
	Mean	2000.03	2069.90	2080.23	2000.00	2064.13	2140.01	2148.19	2000.01	2047.36	2000.16
CEC_17/20	Median	2000.00	2052.41	2049.09	2000.00	2044.78	2124.64	2147.36	2000.00	2033.25	2000.31
	Max	2000.57	2172.84	2191.00	2000.00	2189.77	2290.38	2318.93	2000.31	2196.48	2000.31
	Std	0.10	41.34	57.84	0.00	49.65	62.09	71.40	0.04	47.01	0.16
	Min	2200.00	2200.00	2200.00	2212.26	2200.03	2206.74	2200.10	2200.00	2200.00	2200.00
	Mean	2208.96	2231.38	2259.97	2264.83	2306.31	2305.83	2303.38	2273.53	2241.59	2228.10
CEC_17/21	Median	2200.00	2202.29	2203.24	2255.08	2317.26	2332.06	2333.67	2303.90	2202.11	2200.00
	Max	2304.63	2350.22	2349.48	2319.04	2332.98	2398.64	2404.68	2307.78	2336.21	2302.84
	Std	19.66	54.68	61.84	37.49	35.66	61.37	70.63	47.08	58.33	45.68
	Min	2200.00	2239.42	2211.20	2258.63	2301.00	2247.75	2231.19	2200.00	2219.29	2300.00
	Mean	2250.02	2307.06	2298.89	2296.80	2304.74	2313.36	2308.94	2298.07	2299.93	2300.00
CEC_17/22	Median	2232.00	2307.09	2301.92	2300.57	2305.89	2313.40	2313.52	2300.00	2302.45	2300.00
	Max	2300.35	2321.07	2303.37	2301.42	2309.04	2336.06	2330.27	2300.35	2309.11	2300.00
	Std	39.37	10.83	15.49	9.53	2.91	11.94	20.33	14.01	14.93	0.00
	Min	2300.00	2609.40	2605.14	2607.02	2606.54	2615.62	2610.56	2602.86	2604.32	2600.00
	Mean	2599.22	2629.83	2624.95	2611.41	2619.61	2644.45	2657.81	2604.60	2624.02	2600.44
CEC_17/23	Median	2605.91	2627.48	2624.94	2611.60	2619.57	2642.83	2658.93	2604.60	2621.87	2600.00
	Max	2613.12	2663.67	2665.34	2615.19	2632.72	2695.53	2695.42	2607.27	2656.68	2604.11
	Std	42.83	11.90	12.33	1.86	5.90	16.34	20.39	1.02	10.90	1.13
	Min	2500.00	2429.87	2500.00	2620.00	2500.36	2501.92	2500.56	2500.00	2500.00	2500.00
	Mean	2545.93	2726.69	2726.34	2717.77	2742.39	2753.22	2771.01	2717.68	2689.53	2554.74
CEC_17/24	Median	2500.00	2763.09	2748.54	2743.72	2747.15	2777.74	2793.75	2732.03	2745.95	2500.00
	Max	2734.93	2823.81	2800.40	2750.26	2764.90	2856.97	2882.39	2735.65	2775.27	2729.54
	Std	68.36	104.54	79.72	40.92	34.99	85.54	94.40	50.33	112.35	91.60
	Min	2600.03	2600.19	2897.74	2899.84	2897.79	2606.80	2609.28	2897.74	2897.74	2897.74
	Mean	2896.52	2929.07	2923.15	2911.91	2924.10	2941.23	2926.75	2915.07	2925.92	2919.48
CEC_17/25	Median	2897.75	2946.50	2943.47	2909.79	2943.42	2951.52	2945.58	2898.03	2944.18	2899.58
	Max	2943.44	3024.18	2949.15	2944.47	2948.56	3052.13	2972.85	2945.80	2971.76	2943.37
	Std	44.49	53.74	23.30	10.71	23.50	66.69	51.10	22.42	25.43	22.73
	Min	2600.00	2600.00	2600.00	2715.72	2600.57	2601.90	2811.59	2900.00	2600.00	2800.00
	Mean	2833.28	3019.96	2885.47	2896.64	3004.37	3332.97	3316.84	2900.00	2953.87	2884.61
CEC_17/26	Median	2884.67	3025.99	2900.00	2918.33	2900.17	3137.58	3206.68	2900.00	2900.00	2900.00
	Max	2900.00	3462.30	3044.26	2952.48	3997.45	4801.41	4552.35	2900.00	3857.35	2900.00
	Std	86.07	153.11	81.83	54.60	305.99	487.08	426.84	0.00	173.27	36.08
	Min	3086.89	3089.52	3089.01	3089.05	3073.32	3090.24	3097.14	3089.01	3089.64	3089.52
	Mean	3088.66	3099.77	3106.63	3089.69	3079.47	3122.82	3131.65	3089.45	3103.45	3091.80
CEC_17/27	Median	3089.01	3097.51	3097.80	3089.66	3078.33	3106.23	3120.99	3089.52	3095.74	3090.75
	Max	3089.71	3131.03	3208.63	3090.47	3094.43	3221.35	3219.83	3089.52	3193.05	3095.98
	Std	0.82	8.74	27.44	0.25	4.22	35.35	30.88	0.18	22.92	2.38
	Min	2800.00	3100.00	3100.00	3172.85	3100.18	3100.40	3101.60	3100.00	3100.00	3100.00
	Mean	3200.01	3310.54	3199.33	3236.69	3235.88	3403.55	3337.01	3214.03	3330.47	3100.00
CEC_17/28	Median	3100.00	3383.75	3100.00	3210.61	3222.55	3411.82	3383.87	3100.00	3402.69	3100.00
	Max	3411.82	3411.82	3444.13	3411.82	3300.00	3749.37	3501.44	3411.82	3411.82	3100.00
	Std	158.46	124.69	135.72	66.54	64.23	185.04	115.05	145.87	120.05	0.00
	Min	3129.61	3145.87	3148.21	3145.35	3137.22	3156.68	3160.31	3128.05	3145.56	3128.28
	Mean	3140.45	3233.85	3212.09	3161.97	3175.42	3308.50	3300.66	3138.34	3234.63	3132.96
CEC_17/29	Median	3137.70	3226.35	3199.64	3161.65	3168.03	3300.99	3294.46	3138.01	3224.14	3132.98
	Max	3174.45	3412.72	3331.33	3178.83	3300.25	3473.89	3479.31	3154.30	3359.96	3139.27
	Std	9.88	58.82	45.48	7.60	30.91	77.33	75.93	5.89	55.10	2.43
	Min	3408.33	4296.06	3762.14	4657.82	4676.02	6296.85	4838.12	3394.50	3416.36	3394.50
	Mean	3451.00	150294.12	167688.20	17934.90	24185.38	564983.34	527231.40	99555.88	395498.50	3409.27
CEC_17/30	Median	3454.01	16180.83	6759.34	13574.83	15177.31	220962.22	121932.13	3442.66	3729.03	3407.44
	Max	3503.40	1237436.14	1261343.43	67896.82	168236.16	2136927.89	4219938.92	820578.06	1827680.48	3600.49
	Std	23.51	345469.25	359033.99	12874.06	27828.94	630800.00	851685.44	265899.84	540967.78	31.24

**Table A5:** Statistical criteria results of PCOA and other algorithms on the 30D CEC2017, over 51 independent runs.

Function		PCOA	AVOA	PSO	DE	RW_GWO	WOA	HHO	LSHADE	GBO	EBOwithCMAR
	Min	100.00	102.98	106.52	212.81	38377.90	665661.68	4499846.11	100.00	100.01	100.00
	Mean	100.00	4224.24	4050.68	1000.45	5136068.49	2268995.66	8054047.76	100.00	357.29	100.00
CEC_17/01	Median	100.00	2025.53	1986.28	587.59	3818964.65	1898323.36	8009196.91	100.00	156.76	100.00
	Max	100.00	19388.31	18359.26	5040.67	33430312.04	6686501.55	12662974.38	100.00	3532.36	100.00
	Std	0.00	5616.02	4865.16	1056.94	5856386.97	1315520.78	1815136.59	0.00	575.82	0.00
	Min	300.00	300.00	300.00	47264.28	405.55	39936.40	462.04	300.00	300.00	300.00
	Mean	300.00	302.89	300.00	77421.16	1925.99	167967.21	973.16	300.00	300.00	300.00
CEC_17/03	Median	300.00	300.00	300.00	78391.28	1336.95	168353.99	906.63	300.00	300.00	300.00
	Max	300.00	373.48	300.00	98397.77	6154.85	367619.37	2557.15	300.00	300.00	300.00
	Std	0.00	14.40	0.00	10561.32	1520.83	75225.44	378.24	0.00	0.00	0.00
	Min	400.00	400.73	400.00	487.35	440.77	483.63	440.26	400.00	400.03	400.00
	Mean	400.39	489.19	436.16	488.98	483.89	553.91	515.69	455.23	434.55	445.44
CEC_17/04	Median	400.00	488.35	447.78	488.68	486.23	547.25	519.29	458.56	404.44	458.56
	Max	403.99	534.77	487.59	493.69	593.64	675.67	633.33	464.12	510.29	467.90
	Std	1.20	25.08	30.71	1.24	36.46	43.13	30.06	13.97	36.26	26.25
	Min	500.99	621.38	552.73	607.68	561.60	630.76	649.15	508.25	577.61	503.98
	Mean	506.63	699.67	617.07	629.22	655.14	768.39	716.81	515.22	644.09	514.29
CEC_17/05	Median	506.96	701.06	611.44	629.25	654.70	768.62	718.20	515.14	647.25	514.92
	Max	512.93	793.51	685.06	649.54	697.00	904.92	793.23	522.55	750.73	519.90
	Std	2.63	38.33	28.97	8.97	23.45	53.97	30.14	3.04	37.53	3.40
	Min	600.00	617.78	600.35	600.00	600.09	642.00	632.14	600.00	601.49	600.00
	Mean	600.00	631.03	619.54	600.00	600.61	668.05	656.41	600.00	618.86	600.00
CEC_17/06	Median	600.00	630.55	618.99	600.00	600.53	667.82	657.00	600.00	615.65	600.00
	Max	600.00	645.96	648.00	600.00	601.68	692.51	671.42	600.00	645.56	600.00
	Std	0.00	7.36	11.05	0.00	0.39	10.07	7.85	0.00	9.99	0.00
	Min	733.09	890.89	782.91	832.55	771.27	970.15	1044.04	740.79	814.63	733.71
	Mean	736.96	1062.40	819.56	865.42	827.69	1219.07	1200.22	744.47	911.64	739.01
CEC_17/07	Median	736.22	1066.84	815.21	867.17	808.97	1227.84	1205.24	744.41	903.90	737.41
	Max	757.78	1195.42	873.22	880.81	948.63	1437.82	1317.70	749.17	1049.18	747.06
	Std	3.91	69.18	22.38	8.85	47.23	113.93	58.39	2.11	53.20	4.08
	Min	802.98	891.92	849.75	897.53	851.84	918.73	889.65	808.37	866.66	803.98
	Mean	807.14	952.39	898.01	931.39	950.07	1001.24	948.79	815.83	929.93	814.79
CEC_17/08	Median	806.96	951.23	893.53	931.45	954.46	988.21	945.44	816.16	931.33	815.92
	Max	810.94	1029.83	948.25	949.73	1007.11	1127.57	1002.39	826.00	994.02	820.89
	Std	1.93	27.01	23.36	10.01	28.89	50.08	27.20	3.37	28.61	3.77
	Min	900.00	3304.25	942.75	900.00	900.85	4045.01	4192.92	900.00	1269.23	900.00
	Mean	900.00	4960.05	2376.94	900.00	1072.20	7624.71	5741.65	900.04	2415.88	900.00
CEC_17/09	Median	900.00	4965.89	2556.07	900.00	950.54	6605.28	5746.24	900.00	2223.02	900.00
	Max	900.00	8133.58	4931.76	900.00	3758.81	19864.97	7353.05	900.45	4728.04	900.00
	Std	0.00	875.03	798.98	0.00	428.97	3182.47	627.85	0.09	827.60	0.00
	Min	2814.56	3600.94	3151.84	5570.60	5449.52	4456.07	3353.79	1959.27	3550.19	1915.75
	Mean	3544.44	5250.50	4603.72	6250.93	6555.74	5940.29	5328.90	2639.91	4875.87	2572.14
CEC_17/10	Median	3520.11	5250.54	4570.15	6264.21	6562.91	6004.70	5343.33	2694.72	4835.05	2618.47
	Max	4172.49	7671.22	5857.98	6874.20	7815.49	7265.31	6930.96	3075.74	6876.76	2978.07
	Std	358.80	721.49	665.92	302.42	608.37	719.10	662.72	266.31	660.29	268.76
	Min	1100.00	1149.29	1156.91	1172.03	1137.22	1291.20	1154.68	1105.97	1119.91	1101.99
	Mean	1104.90	1227.75	1211.95	1211.34	1236.15	1492.07	1251.09	1129.86	1228.93	1111.86
CEC_17/11	Median	1103.98	1229.11	1206.54	1212.00	1227.70	1466.25	1256.38	1121.90	1229.36	1108.95
	Max	1112.93	1314.20	1331.11	1230.11	1349.63	1706.81	1391.59	1181.87	1336.77	1168.93
	Std	2.60	40.64	38.37	12.62	53.21	106.58	47.02	23.32	51.24	14.52
	Min	1204.05	120736.43	3476.54	1928477.57	377318.88	2378332.84	1302535.90	1667.76	3327.10	1559.83
	Mean	1395.63	1100703.62	24088.16	4790126.13	9559114.35	44028601.18	8155343.18	2388.74	25753.76	2262.83
CEC_17/12	Median	1352.02	809576.42	23307.24	4514042.73	4376168.29	38116682.71	8424103.30	2338.33	22029.89	2236.68
	Max	1816.58	3563762.94	54707.01	9741650.12	59516470.09	136707295.16	19384765.91	3368.97	61419.69	3222.54
	Std	133.06	748699.40	11032.90	1613361.15	12304373.82	29508877.67	4214263.45	399.49	15347.92	362.11
	Min	1312.81	7660.82	1343.03	30473.74	35773.36	30091.76	35522.10	1305.97	1408.25	1303.00
	Mean	1344.16	28911.06	16171.10	146726.65	980345.93	144160.58	206240.87	1350.26	9108.23	1323.80
CEC_17/13	Median	1341.27	25854.18	7967.07	133370.81	136741.35	123359.28	163679.27	1334.62	5430.03	1321.78
	Max	1401.83	80043.26	62326.73	354936.28	7774765.83	384604.94	784358.31	1910.22	36223.86	1391.54
	Std	17.02	14649.55	16821.97	69413.70	1848724.45	87239.35	151738.41	83.12	8514.52	13.01
	Min	1423.98	2199.83	1638.88	11935.62	1715.83	23294.09	1947.64	1422.12	1520.90	1420.04
	Mean	1432.19	19239.67	4163.46	53701.50	31337.86	955063.45	29981.07	1428.79	1684.61	1427.89
CEC_17/14	Median	1430.95	12833.49	3348.02	47578.41	31225.57	463613.62	15836.48	1427.00	1668.28	1427.27
	Max	1446.87	58848.90	11083.32	138042.59	113684.69	3824324.28	182969.26	1450.91	1893.95	1437.93
	Std	5.38	16640.26	2309.51	25009.51	25513.18	1088994.45	36465.09	5.08	77.56	3.77
	Min	1502.43	2526.12	1671.24	4990.13	12331.29	6749.46	7755.33	1504.08	1609.74	1503.06
	Mean	1508.54	14482.50	11147.89	18124.53	54752.89	64357.96	49555.97	1525.09	4791.77	1511.50
CEC_17/15	Median	1507.47	10159.23	7065.03	14642.30	38400.49	51815.01	36241.07	1518.17	2776.77	1510.30
	Max	1531.31	45212.56	42505.78	51425.50	174560.03	264670.50	330469.83	1620.85	34792.95	1534.86
	Std	5.02	11175.95	10247.63	11210.95	38705.50	47347.85	50979.42	22.68	5478.39	6.02
	Min	1618.63	2001.29	2080.19	1858.60	1757.82	2628.65	2342.21	1629.54	1967.64	1602.80
	Mean	2007.96	2766.62	2573.18	2219.25	2470.15	3470.23	3077.23	1845.49	2651.18	1728.31
CEC_17/16	Median	2007.61	2775.27	2563.02	2207.27	2502.98	3499.70	3056.74	1866.02	2636.80	1729.03
	Max	2402.45	3560.38	3372.83	2461.32	3174.82	4992.73	3846.58	2092.32	3280.31	1978.39
	Std	178.70	350.94	292.69	128.78	335.57	503.71	350.49	123.12	316.06	107.19
	Min	1728.72	1785.89	1773.35	1804.48	1763.58	1996.83	1925.65	1723.09	1827.40	1710.19
	Mean	1756.85	2374.58	2160.49	1872.42	1944.33	2505.29	2479.08	1750.36	2267.10	1732.73
CEC_17/17	Median	1748.28	2409.20	2160.03	1869.52	1908.98	2522.54	2417.80	1748.21	2251.78	1735.06
	Max	1894.07	2974.73	2670.06	1957.60	2559.81	2965.75	3196.54	1862.02	2785.67	1748.07
	Std	32.24	248.37	186.86	37.72	137.17	260.12	297.26	19.68	203.65	9.05
	Min	1834.13	31369.81	9221.01	139286.37	58623.46	106357.24	44349.37	1821.42	2697.75	1823.29
	Mean	1850.49	196418.28	81372.01	434267.58	426071.81	2378285.00	551704.58	1870.87	17325.42	1843.23
CEC_17/18	Median	1849.17	151462.26	69280.04	394068.42	313723.60	1407405.99	418531.59	1851.22	14203.73	1836.22
	Max	1870.95	700979.12	205596.08	1030494.17	2123401.96	11153514.86	2187907.17	2228.37	65205.56	1931.80
	Std	8.35	153670.44	41025.70	198408.22	378599.12	2670246.23	522709.93	66.95	14492.49	22.00
	Min	1908.10	2198.05	2004.66	6791.08	8940.79	305811.87	20034.00	1904.56	1937.65	1904.79
	Mean	1916.22	13104.75	8094.25	20373.55	119739.32	3042004.00	167480.21	1914.71	2476.18	1913.38
CEC_17/19	Median	1916.15	9274.25	4551.45	18876.17	74442.16	2511558.92	159769.80	1911.43	2105.42	1911.50
	Max	1925.30	52272.64	35267.60	64664.69	663763.90	12760968.24	406243.80	1951.43	8195.16	1937.99
	Std	3.49	12669.47	8101.85	10908.20	123601.86	2539552.67	107615.39	9.71	1142.17	6.30
	Min	2027.65	2114.61	2207.68	2049.38	2131.39	2342.07	2263.56	2030.19	2123.39	2017.40
	Mean	2090.19	2545.71	2463.99	2147.24	2370.87	2697.81	2664.92	2078.53	2431.03	2062.60
CEC_17/20	Median	2060.46	2542.39	2458.83	2128.83	2344.58	2694.95	2671.11	2048.39	2437.53	2041.63
	Max	2217.96	2923.51	3070.79	2268.86	2730.48	3198.92	3144.70	2175.35	2818.73	2163.91
	Std	59.01	183.45	184.22	55.23	137.49	201.51	206.20	54.18	178.13	45.71
	Min	2200.00	2392.67	2345.60	2404.06	2342.50	2468.86	2398.43	2310.30	2356.23	2302.99
	Mean	2308.83	2480.64	2406.29	2432.32	2441.72	2559.22	2522.68	2316.63	2415.63	2312.41
CEC_17/21	Median	2309.60	2486.59	2398.34	2434.47	2443.02	2548.67	2519.62	2316.61	2417.02	2313.57
	Max	2324.63	2594.28	2488.09	2449.46	2481.53	2686.51	2667.02	2322.08	2495.22	2320.12
	Std	16.27	50.71	34.36	9.60	22.67	57.94	51.96	2.64	33.89	4.77
	Min	2300.00	2300.00	2300.00	2559.60	2310.52	2314.49	2316.49	2300.00	2300.00	2300.00
	Mean	2300.00	5217.29	3636.20	3254.76	6084.43	6341.02	4902.78	2300.00	3254.30	2300.00
CEC_17/22	Median	2300.00	6308.16	2302.45	3225.11	7258.64	7314.27	6042.55	2300.00	2302.46	2300.00
	Max	2300.00	8175.04	7606.95	4246.95	8508.85	8811.62	7858.86	2300.00	7977.74	2300.00
	Std	0.00	2129.19	2039.38	333.78	2506.43	2318.24	2321.01	0.00	1759.59	0.00
	Min	2644.69	2788.25	2715.95	2747.63	2704.46	2847.34	2914.39	2650.05	2713.95	2652.03
	Mean	2660.84	2897.02	2826.55	2779.46	2811.14	3055.07	3048.60	2665.11	2788.20	2660.83
CEC_17/23	Median	2658.62	2898.06	2827.39	2780.46	2811.92	3062.16	3032.12	2665.80	2789.27	2660.63
	Max	2679.33	3024.60	2983.14	2795.50	2863.25	3334.17	3247.26	2674.96	2914.16	2671.19
	Std	9.14	63.75	47.59	9.37	24.68	102.82	78.36	5.02	43.80	4.34
	Min	2826.46	2951.23	2895.97	2955.73	2924.29	2972.52	3041.84	2828.20	2880.57	2600.00
	Mean	2836.71	3114.91	2990.45	2983.34	2968.64	3155.93	3345.31	2835.97	2949.61	2826.84
CEC_17/24	Median	2832.99	3111.11	2985.80	2984.03	2968.28	3157.83	3364.12	2835.73	2943.68	2831.60
	Max	2853.29	3326.07	3134.56	3002.68	3018.77	3354.54	3641.83	2845.93	3084.74	2841.04
	Std	7.53	83.47	56.86	10.30	17.92	94.77	131.14	3.33	44.55	32.59
	Min	2883.39	2883.83	2883.48	2887.08	2884.17	2889.25	2884.33	2886.74	2883.72	2883.61
	Mean	2883.84	2900.64	2891.52	2887.33	2903.82	2938.48	2907.97	2886.84	2896.51	2886.65
CEC_17/25	Median	2883.44	2890.40	2887.94	2887.36	2904.20	2944.49	2908.69	2886.83	2889.71	2886.79
	Max	2887.12	2945.48	2941.42	2887.62	2940.71	2978.91	2950.68	2887.06	2940.78	2888.37
	Std	1.12	17.75	10.54	0.10	10.74	25.17	17.57	0.07	15.18	0.79
	Min	2800.00	2900.00	2800.00	4746.81	2901.70	3815.79	2864.07	3595.14	2800.00	2800.00
	Mean	3351.93	6193.00	4548.70	4931.23	5181.01	7397.92	6699.50	3691.92	4917.60	3544.95
CEC_17/26	Median	3590.58	6421.87	4980.99	4936.54	5244.56	7545.58	6956.79	3688.00	5152.93	3589.33
	Max	3996.54	8311.80	6911.57	5071.31	5868.04	9707.03	8755.10	3796.25	7554.15	3760.51
	Std	460.33	1185.95	1350.94	66.71	443.32	1282.68	1294.23	48.29	1152.94	221.23
	Min	3180.61	3209.05	3204.78	3201.77	3161.41	3251.25	3221.17	3181.59	3208.95	3182.53
	Mean	3200.89	3271.09	3249.76	3209.04	3198.05	3367.50	3312.66	3205.39	3248.64	3205.07
CEC_17/27	Median	3201.75	3259.92	3244.79	3209.00	3200.01	3342.96	3299.11	3206.41	3243.92	3205.45
	Max	3230.25	3416.33	3330.63	3213.64	3200.01	3696.48	3522.39	3224.62	3342.70	3226.91
	Std	9.43	39.14	28.08	2.47	8.11	90.82	64.17	8.24	30.46	6.98
	Min	3100.00	3100.00	3100.00	3208.26	3289.66	3236.60	3111.29	3100.00	3100.00	3100.00
	Mean	3100.00	3206.95	3141.43	3224.35	3299.80	3302.67	3241.66	3139.95	3133.20	3111.33
CEC_17/28	Median	3100.00	3209.17	3100.00	3221.75	3300.01	3298.13	3251.39	3100.00	3100.00	3100.00
	Max	3100.00	3266.64	3260.11	3265.69	3300.01	3498.62	3285.49	3266.64	3261.85	3253.93
	Std	0.00	42.59	57.89	11.09	1.45	44.02	29.58	55.62	55.95	35.25
	Min	3281.91	3443.19	3371.87	3473.61	3377.66	3895.34	3526.75	3288.74	3510.59	3278.90
	Mean	3451.27	4013.32	3779.36	3638.74	3710.66	4745.22	4261.91	3358.02	3948.38	3338.64
CEC_17/29	Median	3428.50	4071.52	3745.07	3642.60	3729.95	4721.59	4274.94	3359.23	3908.64	3335.43
	Max	3661.95	4603.29	4388.51	3799.41	4104.16	5734.41	4883.95	3453.61	4438.80	3444.73
	Std	81.12	270.07	227.78	78.59	192.77	358.21	280.45	24.36	232.72	23.70
	Min	5159.96	10476.10	5310.53	10044.83	177700.27	667293.22	162969.89	4941.81	5069.54	4984.43
	Mean	5543.69	28572.87	8248.09	24304.51	919685.11	9513847.27	885053.81	5088.99	7536.31	5131.40
CEC_17/30	Median	5536.86	27824.48	6880.08	23438.21	684404.15	8068347.15	858471.02	5052.27	6953.51	5078.41
	Max	5845.63	57458.41	16725.47	42467.45	3166194.32	30866546.23	2056085.10	6146.01	14109.65	5879.85
	Std	137.41	11102.58	2882.37	6656.45	658333.94	6809939.36	387608.27	181.57	2052.50	157.63

**Table A6:** Statistical criteria results of PCOA and other algorithms on the 50D CEC2017, over 51 independent runs.

Function		PCOA	AVOA	PSO	DE	RW_GWO	WOA	HHO	LSHADE	GBO	EBOwithCMAR
	Min	100.00	100.17	100.00	605.87	1670157.63	1684606.05	22820942.68	100.00	100.17	100.00
	Mean	100.00	4577.60	4447.91	8518.81	47380363.48	8174316.10	33116463.10	100.00	3289.60	100.00
CEC_17/01	Median	100.00	3236.96	2371.44	6211.29	44898327.03	6977503.00	31911908.27	100.00	1947.05	100.00
	Max	100.01	18512.74	24284.22	49166.80	128007344.64	25573323.10	48631908.15	100.00	18347.79	100.00
	Std	0.00	4578.47	6185.88	8557.35	28212702.75	5530006.96	5719541.68	0.00	3661.13	0.00
	Min	300.00	306.20	1228.41	137505.56	1431.51	28954.53	1311.27	300.00	300.00	300.00
	Mean	300.00	998.55	5926.34	195430.08	14082.67	78789.73	2653.51	300.00	300.15	300.00
CEC_17/03	Median	300.00	684.57	5042.26	197005.47	12760.09	66596.15	2586.33	300.00	300.05	300.00
	Max	300.00	4739.99	13706.63	230895.63	27996.63	202623.84	5566.20	300.00	302.35	300.00
	Std	0.00	998.83	3439.55	18389.98	6070.63	38408.67	931.04	0.00	0.36	0.00
	Min	400.00	403.99	400.15	504.27	469.50	566.49	453.15	400.00	400.16	400.00
	Mean	400.08	523.82	482.14	538.89	595.10	685.88	622.07	455.45	475.90	434.26
CEC_17/04	Median	400.00	541.42	497.41	530.26	598.10	684.39	623.37	428.51	475.35	428.51
	Max	403.99	618.09	553.03	617.55	693.39	958.28	791.56	542.31	563.58	542.31
	Std	0.56	50.54	41.67	26.78	52.87	66.72	59.94	52.55	47.77	43.49
	Min	508.95	720.88	638.30	782.97	619.14	806.99	788.76	520.15	705.96	524.87
	Mean	515.24	830.73	721.98	816.85	848.03	920.24	861.84	532.98	793.92	534.24
CEC_17/05	Median	514.92	837.46	716.90	818.73	862.98	903.70	867.18	533.17	797.49	533.83
	Max	523.88	920.86	825.35	845.72	922.78	1160.48	920.32	548.38	891.01	548.75
	Std	3.52	41.21	41.75	15.95	70.25	75.42	30.98	4.91	39.88	4.70
	Min	600.00	628.26	612.56	600.00	600.42	657.25	648.98	600.00	618.75	600.00
	Mean	600.00	636.55	635.33	600.00	602.59	675.59	667.58	600.00	636.30	600.00
CEC_17/06	Median	600.00	635.10	636.33	600.00	602.01	672.95	667.40	600.00	636.14	600.00
	Max	600.00	653.38	649.77	600.00	609.63	699.53	678.32	600.00	662.69	600.00
	Std	0.00	6.11	8.05	0.00	1.85	10.19	5.48	0.00	9.58	0.00
	Min	757.08	1241.20	856.68	1010.87	875.15	1209.27	1525.98	771.21	1020.05	772.87
	Mean	767.65	1445.83	964.49	1073.10	946.70	1689.24	1714.36	782.10	1227.97	781.21
CEC_17/07	Median	766.49	1445.43	954.03	1074.41	937.93	1673.31	1718.57	781.79	1218.88	781.85
	Max	805.23	1638.29	1087.63	1096.50	1142.95	1998.46	1902.58	791.55	1463.82	792.71
	Std	7.86	106.54	47.99	13.89	49.87	133.97	96.50	4.46	111.88	4.02
	Min	808.95	1034.81	963.17	1090.66	909.96	1069.19	1067.37	824.24	968.15	825.87
	Mean	816.68	1139.65	1045.09	1116.78	1136.92	1207.07	1155.61	831.90	1097.32	834.57
CEC_17/08	Median	816.91	1140.27	1036.80	1116.47	1148.04	1205.91	1162.08	831.30	1092.52	833.83
	Max	826.86	1248.21	1151.22	1141.02	1223.58	1360.98	1230.03	845.41	1204.35	848.75
	Std	3.98	43.45	44.20	11.75	78.35	70.14	37.00	4.30	55.07	4.62
	Min	900.00	10019.08	2530.20	900.00	1045.18	11991.17	11841.15	900.00	2861.72	900.00
	Mean	900.00	12890.15	7678.15	900.48	3808.95	20535.21	16271.33	901.10	7060.11	900.64
CEC_17/09	Median	900.00	12782.68	7372.11	900.03	3013.66	19032.21	15672.13	900.81	6799.83	900.54
	Max	900.00	16395.42	14306.88	902.98	15843.34	45133.38	21972.05	906.90	12180.18	902.73
	Std	0.00	1472.61	2319.52	0.82	2634.10	6412.27	2072.99	1.29	2249.90	0.64
	Min	3332.50	6042.83	5546.07	11154.90	9856.37	7707.26	6220.21	3652.64	4825.42	3136.77
	Mean	5130.57	8087.00	7460.04	12207.68	12087.03	9816.14	8451.49	4353.74	7772.80	4069.16
CEC_17/10	Median	5145.32	8070.98	7405.04	12224.63	12122.56	10113.93	8472.94	4346.98	7710.24	4100.14
	Max	6544.60	10039.90	10672.11	12727.89	14207.41	12600.48	11624.49	4918.79	10282.92	4622.84
	Std	611.83	835.46	1039.71	312.87	753.10	1209.41	1105.83	283.20	961.84	387.44
	Min	1122.24	1215.29	1195.81	1264.32	1228.31	1357.63	1286.76	1166.02	1263.53	1146.76
	Mean	1130.44	1326.52	1271.23	1286.75	1324.45	1567.94	1401.06	1233.41	1371.14	1185.64
CEC_17/11	Median	1130.20	1324.32	1257.56	1287.50	1327.23	1550.88	1388.34	1226.71	1364.97	1182.88
	Max	1137.17	1502.39	1350.09	1309.98	1541.03	1837.24	1645.54	1317.20	1499.33	1236.66
	Std	3.31	60.99	39.84	11.89	49.60	111.42	77.61	37.52	59.55	21.55
	Min	1502.54	541664.81	22885.18	22183437.30	3945280.51	36435630.20	15666921.05	3198.00	41347.21	2155.25
	Mean	2036.39	4334805.02	167111.75	48841523.31	55849602.53	211041964.56	60461136.84	7080.84	133934.55	4241.58
CEC_17/12	Median	2029.42	3478776.80	167428.00	45793562.32	28795701.87	192956616.65	58738578.48	6750.50	126105.55	3533.97
	Max	2536.91	11233159.09	330719.18	90649117.45	248514634.63	544732228.03	139533682.69	16107.62	381628.58	16989.93
	Std	224.63	2483709.01	75454.20	16916756.74	69329851.47	119281845.43	28163750.13	3047.10	74127.81	2356.40
	Min	1344.81	17950.58	1538.71	44047.62	60569.90	43545.93	353191.48	1336.81	1610.43	1357.71
	Mean	1399.54	49641.60	4604.70	195786.26	1470233.59	212953.29	1202608.24	1584.81	7795.81	1551.59
CEC_17/13	Median	1394.40	46756.82	3141.51	153888.96	260512.52	174262.04	992346.34	1519.43	4750.71	1498.09
	Max	1486.47	111187.29	21185.68	587126.47	26607924.61	853813.02	3601114.97	2730.35	31392.02	2425.29
	Std	30.76	21547.51	4332.33	125660.19	5003204.22	161046.90	642965.60	227.99	6572.06	189.65
	Min	1462.78	7829.86	2591.68	129784.28	7530.13	30026.58	30847.87	1510.58	1708.43	1467.78
	Mean	1510.64	73692.52	22866.05	501344.80	129492.00	562872.06	225388.93	1626.82	3160.28	1571.88
CEC_17/14	Median	1510.54	57312.01	17805.62	489629.74	108810.75	432352.25	183623.24	1603.09	2825.03	1562.28
	Max	1548.35	261793.38	74444.19	1085071.94	408864.93	2195056.48	759905.37	1844.88	8012.41	1708.56
	Std	19.22	61771.02	17749.84	203768.77	97343.30	456415.83	149083.37	76.73	1303.78	50.81
	Min	1543.21	6723.19	1841.29	12930.67	20479.62	19431.49	29535.92	1596.54	1650.06	1588.60
	Mean	1578.91	22042.42	7025.29	31395.13	330386.02	80865.55	178630.38	1823.88	9768.76	1732.96
CEC_17/15	Median	1577.05	20638.55	5305.91	28421.23	72135.47	61928.35	172952.17	1801.54	7890.76	1727.40
	Max	1621.91	39457.70	18283.74	103037.27	6284859.36	563154.26	482990.18	2151.67	32625.69	1923.92
	Std	17.90	8315.98	5075.64	17500.64	1139745.62	86140.80	93804.62	135.36	7511.56	83.44
	Min	1848.54	2743.41	2156.60	2761.71	2282.57	3211.69	2990.04	1891.16	2479.80	1739.65
	Mean	2326.64	3741.27	3236.92	3296.68	3417.32	4666.16	4049.99	2310.70	3585.17	2089.60
CEC_17/16	Median	2309.68	3605.40	3206.28	3293.88	3442.22	4582.45	4063.00	2307.25	3572.64	2075.95
	Max	2798.99	4626.45	4331.39	3695.49	4325.93	6396.89	5108.63	2601.15	4859.45	2406.47
	Std	209.81	481.71	471.81	189.67	452.79	681.30	553.28	187.47	488.68	158.76
	Min	1906.08	2994.78	2327.63	2305.85	2120.32	3043.46	2962.24	1915.93	2478.23	1912.16
	Mean	2385.76	3548.55	3063.94	2615.98	2969.23	3936.57	3636.28	2220.16	3142.14	2089.82
CEC_17/17	Median	2383.23	3608.18	3061.83	2629.85	3060.95	3939.66	3606.85	2224.63	3098.16	2093.00
	Max	2837.55	4228.98	3848.87	2909.34	3714.70	4991.35	4372.53	2502.21	3927.08	2276.62
	Std	196.42	300.37	326.63	141.65	400.26	441.69	360.51	130.62	343.61	88.32
	Min	1832.28	48011.63	28154.04	840139.45	237519.92	542752.40	194292.53	1838.56	4422.64	1843.20
	Mean	1849.90	354316.49	73927.78	2501584.71	2261881.46	6809268.87	2093474.45	2018.27	23812.73	1985.11
CEC_17/18	Median	1849.23	287513.31	66770.98	2421316.03	1705677.57	4958884.12	1708668.08	2003.72	17637.23	1978.10
	Max	1868.11	1106454.42	161628.10	5548910.83	11702982.76	23684567.30	7103693.87	2298.13	66047.00	2208.04
	Std	8.50	214449.43	30439.35	995450.01	1901410.71	5925562.18	1547116.39	127.19	16295.27	91.29
	Min	1944.75	3217.72	2029.38	12456.38	29290.32	25079.14	31026.66	1976.69	2303.36	1957.88
	Mean	1970.58	19193.86	14449.09	23141.82	642435.12	2274195.64	360263.60	2063.61	15394.75	2021.88
CEC_17/19	Median	1970.31	14505.56	9862.24	21944.33	343272.63	1977944.78	266742.62	2059.02	16293.59	2021.66
	Max	1996.76	45233.59	39089.58	42341.48	4979804.14	6803584.44	1689025.71	2195.55	38950.41	2093.70
	Std	12.65	12979.34	10236.87	6795.81	993844.02	1799909.54	320833.28	53.89	9665.91	35.16
	Min	2113.88	2631.72	2276.06	2396.19	2282.70	2924.69	2745.34	2094.55	2296.80	2068.02
	Mean	2501.74	3335.42	3047.48	2747.58	3010.75	3646.83	3382.18	2301.69	3156.51	2225.53
CEC_17/20	Median	2486.18	3373.39	3076.87	2752.59	3016.59	3665.40	3394.19	2317.75	3117.15	2235.82
	Max	2834.56	3954.94	3637.10	3030.08	3563.85	4253.41	3963.43	2573.23	3903.22	2422.09
	Std	168.46	323.72	311.47	159.36	285.64	319.59	280.25	129.31	324.58	99.48
	Min	2309.38	2579.89	2456.18	2579.22	2410.93	2708.49	2611.37	2322.16	2431.93	2327.30
	Mean	2317.19	2705.16	2535.43	2625.01	2618.72	2878.00	2784.42	2334.43	2568.09	2335.09
CEC_17/21	Median	2316.93	2700.11	2538.41	2628.95	2643.23	2874.29	2788.48	2335.33	2562.09	2335.39
	Max	2325.50	2810.69	2637.55	2654.08	2709.89	3114.15	2992.26	2344.39	2706.61	2345.97
	Std	3.54	61.10	44.90	17.26	80.99	96.67	78.67	4.54	57.59	3.95
	Min	2300.00	2302.95	6898.72	12342.48	11095.89	9227.51	8458.41	2300.00	2300.00	2300.00
	Mean	2424.86	9840.48	9365.78	13888.82	13516.13	11436.15	10582.39	5344.89	9337.36	3672.80
CEC_17/22	Median	2300.00	9989.38	9431.80	13975.88	13605.58	11395.87	10626.60	6009.33	9750.16	2300.00
	Max	8406.35	12161.63	11245.08	14616.39	14968.16	14998.30	12553.77	6789.42	11640.86	6610.96
	Std	855.11	1411.89	1067.85	440.87	725.31	1182.00	957.25	1464.83	1690.30	1811.90
	Min	2731.54	2976.87	2944.22	3012.29	2811.09	3308.55	3288.83	2739.99	2887.14	2736.18
	Mean	2755.84	3285.08	3127.89	3048.33	3100.27	3618.39	3564.60	2756.57	3079.81	2755.07
CEC_17/23	Median	2755.00	3266.63	3115.82	3048.57	3110.65	3614.99	3542.66	2756.57	3091.27	2755.04
	Max	2779.24	3526.33	3388.63	3069.78	3189.85	3933.48	3916.35	2773.69	3269.47	2772.21
	Std	10.89	111.48	99.51	11.84	65.62	155.09	143.91	8.91	83.30	7.33
	Min	2905.53	3248.89	3052.41	3219.89	3049.09	3303.61	3814.43	2915.14	3047.76	2911.17
	Mean	2931.17	3548.02	3254.22	3260.76	3258.40	3657.97	4135.41	2930.63	3199.21	2928.06
CEC_17/24	Median	2933.07	3560.76	3241.33	3263.11	3257.62	3662.49	4161.98	2929.73	3181.23	2929.25
	Max	2960.90	4019.27	3439.42	3285.52	3354.84	3938.13	4507.13	2953.11	3492.76	2938.01
	Std	14.28	141.97	92.81	16.06	50.17	136.43	178.96	7.91	95.53	5.73
	Min	2928.11	2961.20	2956.81	2980.33	2972.44	3082.32	3055.85	2979.76	2961.73	2928.74
	Mean	2982.25	3066.44	3033.35	2982.35	3090.91	3139.43	3119.34	3003.70	3062.66	3000.42
CEC_17/25	Median	2970.49	3073.20	3031.90	2980.35	3086.77	3134.80	3116.36	2980.40	3068.95	2991.97
	Max	3062.70	3112.73	3089.69	3021.85	3197.86	3205.37	3195.94	3068.65	3115.97	3068.87
	Std	42.70	32.20	41.45	7.79	49.19	31.29	34.90	33.79	34.52	28.16
	Min	2900.00	2900.00	2900.00	6512.07	5062.07	4745.58	2941.17	3785.35	2900.00	3855.22
	Mean	2900.00	9177.14	6762.93	6850.55	7305.15	12708.12	9385.51	4004.13	6649.52	4000.98
CEC_17/26	Median	2900.00	9368.39	7616.96	6856.33	7480.17	12848.07	10417.31	3995.68	7404.67	3994.59
	Max	2900.00	11914.36	9896.62	7074.37	8433.33	18304.43	12448.88	4221.68	11620.56	4206.09
	Std	0.00	1616.64	2441.30	118.53	739.53	2020.60	2718.65	89.51	3405.48	80.87
	Min	3204.39	3429.40	3286.84	3231.31	3200.01	3511.87	3458.62	3211.41	3276.20	3217.63
	Mean	3243.23	3680.69	3511.52	3248.21	3200.01	4217.39	3940.30	3246.49	3613.03	3262.68
CEC_17/27	Median	3232.34	3646.34	3491.55	3246.92	3200.01	4121.60	3892.13	3238.28	3598.62	3255.13
	Max	3331.13	4180.75	3878.28	3290.33	3200.01	5268.64	4906.91	3299.94	4084.62	3386.20
	Std	32.56	175.00	126.57	11.95	0.00	395.00	263.44	24.46	160.98	30.88
	Min	3253.35	3258.85	3253.35	3258.85	3278.78	3325.47	3263.56	3258.85	3258.52	3251.96
	Mean	3255.38	3302.02	3289.99	3259.37	3299.60	3420.71	3365.27	3286.41	3288.23	3284.03
CEC_17/28	Median	3253.35	3307.71	3296.67	3258.85	3300.01	3417.56	3364.36	3307.69	3296.67	3295.20
	Max	3258.85	3323.69	3397.50	3270.47	3300.01	3519.42	3481.01	3307.69	3314.63	3333.39
	Std	2.58	15.66	30.19	2.26	2.97	54.03	38.77	24.29	19.64	24.13
	Min	3345.28	3914.07	3990.26	3688.92	3754.56	5654.72	4335.02	3264.19	3945.35	3222.71
	Mean	3632.76	4804.78	4443.09	4081.53	4450.64	7135.84	5296.51	3351.09	4664.58	3277.07
CEC_17/29	Median	3617.96	4790.17	4413.46	4095.93	4432.81	7014.21	5193.53	3321.21	4616.44	3264.44
	Max	4069.12	5568.79	5310.72	4374.06	5307.27	9444.32	6839.25	3650.91	5687.80	3435.50
	Std	188.01	385.55	296.77	145.98	326.37	943.42	525.59	85.70	437.31	46.92
	Min	582421.93	662354.91	696401.55	850585.87	3377929.99	42870693.14	5586342.07	582411.37	582446.49	582412.37
	Mean	582464.54	1407176.80	879583.04	1162219.88	9028706.16	94011085.54	12390619.55	676334.24	659661.01	670999.37
CEC_17/30	Median	582468.40	1376082.93	879433.83	1142131.95	8106247.96	90051617.53	12662209.20	654647.65	611533.00	664055.13
	Max	582513.56	2605124.91	1125762.23	1478477.88	22851833.55	178721186.09	19096625.20	955209.29	1162667.73	928188.22
	Std	22.22	340680.34	104641.29	162242.13	3711188.45	30853888.68	3053384.13	90357.41	114279.47	73507.51

**Table A7:** Statistical criteria results of PCOA and other algorithms on the 100D CEC2017, over 51 independent runs.

Function		PCOA	AVOA	PSO	DE	RW_GWO	WOA	HHO	LSHADE	GBO	EBOwithCMAR
	Min	100.00	106.36	113.94	117.56	47597183.46	20394203.12	159317986.86	100.00	104.74	100.00
	Mean	100.03	7292.34	9396.68	6915.99	389024453.65	34942862.54	210098155.31	100.00	7066.34	100.00
CEC_17/01	Median	100.03	4314.88	6346.00	3694.66	312892414.16	32873854.80	208910955.17	100.00	3477.29	100.00
	Max	100.10	40027.33	53307.10	36982.74	1449521296.76	82459700.62	255597980.32	100.00	50189.20	100.00
	Std	0.02	8744.79	10688.93	8315.25	278111759.81	11030280.94	21593995.71	0.00	9615.04	0.00
	Min	300.00	8918.53	61770.16	503922.59	61255.51	198647.67	19415.13	300.00	301.65	300.00
	Mean	300.00	20295.04	111828.82	597385.37	106248.26	608994.27	34573.96	300.00	311.64	300.00
CEC_17/03	Median	300.00	19492.19	109143.15	597826.75	104786.41	583665.90	34386.47	300.00	310.19	300.00
	Max	300.00	35773.26	177245.41	676095.86	185637.71	1075156.07	54832.82	300.00	342.99	300.00
	Std	0.00	6439.85	25745.75	38176.92	23947.20	196928.93	6962.76	0.00	8.84	0.00
	Min	400.00	558.71	462.66	597.36	690.79	813.74	656.94	400.00	499.80	400.00
	Mean	400.47	649.66	577.58	626.55	759.28	1015.23	830.76	530.23	632.71	461.11
CEC_17/04	Median	400.00	643.02	587.52	628.40	759.57	1012.52	834.80	541.63	639.06	403.99
	Max	403.99	778.52	683.38	635.71	857.97	1247.64	947.87	625.55	712.64	678.37
	Std	1.30	47.40	52.05	5.41	40.57	94.58	65.52	57.87	42.38	87.32
	Min	528.85	1154.68	956.68	1345.19	830.70	1230.15	1324.37	566.56	1074.09	568.65
	Mean	541.57	1304.37	1120.69	1401.16	1173.62	1427.52	1416.68	600.34	1269.02	590.03
CEC_17/05	Median	539.80	1315.26	1123.83	1405.98	968.56	1413.57	1424.68	600.99	1278.05	590.54
	Max	558.70	1450.17	1311.88	1434.97	1581.45	1714.36	1504.74	627.14	1450.17	611.27
	Std	7.43	62.97	81.53	20.97	305.78	99.59	45.21	13.62	69.89	10.62
	Min	600.00	632.92	631.48	600.00	605.98	662.48	670.81	600.01	631.45	600.00
	Mean	600.00	642.44	645.94	600.00	613.41	680.16	676.44	600.08	647.02	600.00
CEC_17/06	Median	600.00	643.00	646.42	600.00	612.74	677.06	676.39	600.06	648.52	600.00
	Max	600.00	650.78	667.87	600.00	626.45	708.82	681.34	600.21	661.05	600.02
	Std	0.00	4.20	6.70	0.00	4.04	10.35	2.61	0.05	5.83	0.00
	Min	832.38	2376.48	1189.64	1642.50	1321.43	2972.41	2927.66	875.21	1996.15	864.61
	Mean	855.39	2774.21	1489.26	1724.85	1481.62	3243.23	3421.76	910.92	2436.84	881.74
CEC_17/07	Median	851.68	2782.91	1453.34	1724.26	1466.94	3245.97	3434.59	912.22	2346.11	880.44
	Max	919.89	3085.18	1966.92	1769.58	1697.51	3574.33	3644.18	940.91	3026.21	901.88
	Std	17.90	145.88	159.44	25.40	86.87	152.77	119.74	14.87	262.87	8.61
	Min	827.86	1489.50	1295.49	1608.26	1105.93	1688.20	1724.51	869.15	1383.04	870.64
	Mean	839.54	1696.14	1452.59	1698.16	1417.13	1878.96	1855.67	898.88	1627.56	887.28
CEC_17/08	Median	837.81	1692.47	1453.68	1704.44	1225.52	1858.74	1860.78	897.71	1625.81	887.56
	Max	859.70	1880.51	1612.87	1733.68	1916.19	2206.28	1972.05	926.93	1874.54	907.46
	Std	7.50	87.65	76.97	25.45	287.00	110.68	61.03	12.38	96.91	8.18
	Min	900.00	19188.23	13422.43	1342.00	10463.32	24489.16	24051.09	908.95	16066.52	900.91
	Mean	900.00	22622.90	19061.46	1932.65	22940.81	37080.47	29653.90	930.32	20616.59	908.37
CEC_17/09	Median	900.00	22484.84	19039.48	1992.69	17993.22	32944.74	29295.43	925.30	20480.79	906.81
	Max	900.00	27160.69	32497.28	2607.85	42231.28	74169.65	43704.81	1037.96	25898.33	939.60
	Std	0.00	1272.09	3290.08	289.40	9776.38	11443.15	3693.05	20.34	2142.49	7.24
	Min	9016.54	13759.83	11548.80	27479.20	25557.97	15580.23	15382.11	8821.63	12489.90	8966.89
	Mean	10920.61	16370.65	14782.49	29299.73	28340.45	19940.44	18241.74	10345.32	15433.88	9817.42
CEC_17/10	Median	10902.76	16343.15	14847.32	29193.75	28490.16	19838.72	18114.85	10436.24	15508.93	9835.91
	Max	12384.87	20275.97	17402.09	30231.43	30593.49	24937.57	21005.64	11332.06	20267.56	10573.39
	Std	696.62	1554.47	1285.06	535.42	967.79	2168.75	1207.48	537.98	1436.49	428.80
	Min	1239.29	1848.73	1627.09	6354.41	2393.80	4033.27	2478.72	1674.85	1959.72	1500.73
	Mean	1333.43	2277.43	2011.98	8516.60	3116.09	7357.08	2939.04	2208.34	2414.40	1823.79
CEC_17/11	Median	1324.63	2280.45	2031.04	8572.75	3006.41	6847.67	2940.03	2236.43	2353.27	1806.17
	Max	1416.16	2852.79	2340.73	10928.74	5965.62	13729.68	3806.82	2713.59	3097.51	2336.70
	Std	44.46	187.43	175.96	1020.04	579.45	1870.76	214.05	208.45	268.16	178.37
	Min	3112.93	2368449.11	265973.64	289971578.25	56695946.84	149956101.44	112685152.18	6147.37	194903.47	3890.60
	Mean	3754.71	11722791.80	867352.13	370007827.41	187415887.72	632541372.99	290321315.26	20769.66	740216.59	17708.18
CEC_17/12	Median	3760.80	10945699.15	753391.33	367093730.84	191858837.94	644395114.00	283908994.44	13614.30	737664.49	5760.74
	Max	4283.18	24198208.40	2322942.31	463054570.00	313940010.21	1297508667.15	429434170.74	142477.88	1285629.80	96045.21
	Std	275.24	5633631.18	440810.05	39464063.10	71034897.79	246016502.73	79337205.20	19897.79	236865.55	21045.35
	Min	1316.03	16839.43	2019.83	2490.01	95682.29	42607.70	1393374.80	1399.01	2996.14	1483.15
	Mean	1427.70	41399.42	7633.80	12859.15	1915545.17	91569.22	3295965.04	2603.42	10365.71	1842.54
CEC_17/13	Median	1428.47	42419.99	6722.08	8068.77	1239717.51	83491.39	3361944.66	1765.89	8263.50	1702.86
	Max	1590.45	65687.61	23083.99	79267.35	10585325.12	207186.66	5176087.46	10061.48	23496.53	6071.14
	Std	69.91	10696.09	5066.00	12796.95	2109607.46	37464.80	920283.72	1805.33	5435.91	640.58
	Min	1510.54	70094.27	30038.80	4437636.27	467851.83	550918.16	295485.38	1628.25	7359.90	1609.29
	Mean	1550.52	218220.30	159297.81	9187110.98	1402592.17	1656839.85	918039.58	2011.82	25606.63	1908.01
CEC_17/14	Median	1555.31	210613.90	130539.08	9316782.63	1269257.83	1483296.94	842315.29	1993.56	24862.04	1891.61
	Max	1582.18	463861.89	387781.94	16067469.75	3147744.34	5223854.35	2776266.87	2410.78	57903.67	2151.73
	Std	16.58	83524.19	85194.60	2446545.59	644714.34	879379.60	412681.78	188.25	11804.89	106.93
	Min	1534.34	12112.15	1891.81	6968.13	37615.84	21935.67	155297.84	1697.09	2057.17	1660.59
	Mean	1605.89	24360.70	4526.43	37311.25	342701.37	139637.00	1045892.81	1851.45	5765.32	1810.78
CEC_17/15	Median	1606.79	22521.24	3498.30	32551.03	170216.27	60483.32	819538.49	1856.24	4587.87	1812.82
	Max	1679.42	54190.55	16000.41	113427.34	1554972.74	2713486.55	8152670.75	2076.46	20219.62	1962.34
	Std	34.12	8672.17	3074.51	21323.51	377160.39	381475.15	1151919.14	93.86	4420.88	78.76
	Min	2728.48	5139.83	3635.13	7813.65	4365.70	6306.64	5159.01	2960.91	4435.34	2832.72
	Mean	3581.62	6366.10	5309.06	8928.68	7729.06	9425.19	7073.44	3882.19	5788.48	3420.55
CEC_17/16	Median	3572.11	6289.08	5404.45	8976.22	8028.66	9226.19	6966.66	3952.76	5837.70	3352.84
	Max	4632.81	8060.63	6978.37	9657.37	9268.35	13151.66	8906.61	4324.21	8027.44	4191.14
	Std	481.39	697.75	675.23	374.51	943.32	1474.99	785.09	331.77	786.76	299.53
	Min	2749.83	4515.06	3247.45	4730.31	3933.47	5873.63	4888.74	3065.85	4726.02	2834.67
	Mean	3598.85	5916.83	4733.13	5318.08	6177.91	6990.83	6114.33	3573.35	5446.05	3175.20
CEC_17/17	Median	3599.23	5856.80	4716.28	5347.45	6310.98	6964.48	6291.02	3590.64	5330.69	3140.11
	Max	4331.39	7209.78	6256.67	5718.73	7306.02	8629.20	7080.62	4038.46	7107.64	3574.36
	Std	316.36	593.55	631.29	271.42	782.59	702.85	609.64	219.99	489.66	183.62
	Min	1857.31	118075.90	94759.25	10002816.41	239153.08	965001.72	817917.29	2121.40	25727.05	1954.79
	Mean	1901.99	333355.02	265321.50	19578179.65	1996903.93	2096164.85	2016116.41	3694.52	96433.83	2078.29
CEC_17/18	Median	1902.03	320188.66	243642.40	19755450.13	1841653.27	1799820.74	2031950.54	3209.81	95332.61	2057.81
	Max	1938.69	633659.66	698160.60	28866206.78	4433191.06	5041024.10	3261813.33	8092.27	185330.19	2322.14
	Std	16.97	113252.74	115568.25	4963306.71	1063032.69	889773.69	602230.93	1505.49	37561.54	80.37
	Min	1979.22	3645.17	2119.22	5895.21	601831.57	999357.93	884570.46	2056.57	2194.12	2025.04
	Mean	2050.45	11795.75	4578.58	37475.39	2211734.62	14068547.09	3405104.51	2190.07	5837.60	2090.38
CEC_17/19	Median	2040.66	10516.47	3540.85	31933.62	2005437.21	14206280.01	3137545.84	2152.21	3985.29	2088.73
	Max	2150.13	24138.80	13747.13	116706.42	4918732.46	29381540.11	7211833.46	3376.88	19070.20	2235.52
	Std	44.20	5605.63	2817.99	26291.64	1052654.66	7005404.79	1438985.84	204.52	4486.09	41.66
	Min	3038.45	4354.79	3280.58	4929.70	4700.98	4693.24	4328.29	3230.59	3869.38	2923.86
	Mean	3860.31	5660.28	5028.53	5470.97	5795.40	6148.91	5745.00	3696.51	5078.62	3453.90
CEC_17/20	Median	3903.55	5787.09	5089.81	5497.14	5772.72	6019.97	5789.40	3658.06	5089.31	3470.12
	Max	4333.81	6649.63	6025.60	5906.01	6482.45	7460.43	6751.95	4076.48	6102.80	3997.64
	Std	307.11	550.89	553.37	219.39	400.51	600.53	492.82	190.42	443.77	232.17
	Min	2354.83	3134.36	2928.72	3160.61	2589.21	3361.45	3483.50	2402.65	2849.28	2399.60
	Mean	2375.09	3447.98	3119.81	3224.29	2936.03	3886.00	3832.42	2429.30	3082.30	2418.10
CEC_17/21	Median	2374.70	3465.01	3102.94	3222.04	2743.00	3856.07	3821.95	2428.17	3081.61	2417.00
	Max	2397.00	3925.50	3446.58	3264.50	3395.99	4385.72	4278.84	2459.77	3352.96	2440.19
	Std	10.16	155.37	109.21	23.44	308.29	229.76	184.59	13.00	131.12	9.80
	Min	2300.00	15322.46	14134.68	30040.40	28082.65	19041.26	19548.56	11390.79	15023.76	2300.00
	Mean	6416.65	19158.68	18258.87	31420.19	30300.50	24108.83	22444.44	12791.02	18645.42	12082.06
CEC_17/22	Median	2300.00	19179.59	18326.86	31510.94	30375.36	24242.82	22584.01	12975.87	18933.05	12343.47
	Max	18290.62	22760.70	21370.02	32329.56	32837.36	28973.57	27314.35	13655.74	22061.04	13375.30
	Std	6098.59	1555.51	1728.11	461.90	1001.02	2245.82	1586.79	511.17	1582.70	1513.32
	Min	2877.73	3385.37	3541.98	3496.59	3660.48	4227.12	4170.77	2888.00	3383.84	2877.15
	Mean	2908.78	3761.69	3946.65	3547.74	3782.73	4800.32	4508.73	2911.06	3642.01	2906.42
CEC_17/23	Median	2909.49	3735.28	3935.77	3550.78	3786.83	4757.49	4483.89	2911.49	3624.90	2905.21
	Max	2945.80	4213.33	4463.11	3588.41	3927.03	5309.38	4922.31	2949.85	3994.87	2940.03
	Std	16.05	173.54	175.56	21.55	54.85	236.45	186.42	14.84	124.47	12.36
	Min	3323.94	4312.28	3912.05	4050.32	3494.07	5260.83	4866.99	3348.90	3950.78	3332.38
	Mean	3366.32	4716.47	4286.32	4098.86	3864.44	5973.98	5629.99	3399.84	4334.06	3373.11
CEC_17/24	Median	3366.91	4689.91	4261.24	4098.19	3679.54	5942.96	5653.54	3392.46	4317.62	3376.03
	Max	3420.37	5215.92	5012.06	4133.57	4309.87	6797.58	6633.74	3482.26	4952.77	3406.87
	Std	23.65	216.67	250.61	16.79	309.68	386.78	373.24	27.12	244.29	16.67
	Min	3058.73	3145.76	3120.85	3240.10	3436.59	3484.79	3344.22	3133.82	3198.20	3102.08
	Mean	3130.42	3291.65	3252.28	3328.44	3536.15	3616.48	3529.40	3241.06	3294.37	3224.33
CEC_17/25	Median	3109.63	3295.26	3258.19	3320.60	3528.77	3606.51	3525.23	3243.04	3287.75	3216.35
	Max	3242.78	3379.79	3345.70	3460.73	3820.87	3815.45	3712.70	3350.92	3476.38	3334.10
	Std	44.21	60.73	67.54	48.83	84.32	79.61	70.28	49.07	58.88	53.39
	Min	2900.00	15671.07	2900.00	14013.49	8777.62	25783.35	4660.26	6332.19	2900.00	6250.89
	Mean	6533.21	21071.83	14736.49	14553.56	11844.71	31617.05	23849.18	6792.19	18327.89	6564.92
CEC_17/26	Median	6586.69	20683.79	15842.00	14558.10	10076.84	31456.75	24353.22	6781.11	17959.83	6605.46
	Max	7405.62	26174.51	21962.55	15059.20	16794.81	39277.38	27392.53	7302.08	24343.20	6969.08
	Std	587.15	2353.12	5296.76	221.32	2950.54	3300.90	3089.12	203.27	3820.78	149.17
	Min	3304.29	3547.54	3484.85	3470.13	3200.02	3788.71	3662.10	3301.55	3445.33	3277.20
	Mean	3345.13	3905.64	3790.36	3543.66	3200.02	4974.13	4110.94	3373.20	3713.94	3353.90
CEC_17/27	Median	3341.02	3883.65	3773.45	3543.87	3200.02	4991.77	4086.12	3371.41	3702.54	3350.82
	Max	3404.09	4259.91	4090.91	3602.52	3200.02	6648.73	4819.35	3434.38	4172.46	3425.64
	Std	25.90	182.96	139.59	27.09	0.00	586.10	255.86	33.32	156.52	26.75
	Min	3100.00	3307.31	3288.49	3327.32	3300.02	3597.66	3428.13	3209.63	3301.39	3100.00
	Mean	3155.84	3366.18	3346.01	3347.40	3300.02	3718.60	3570.03	3324.66	3369.76	3283.82
CEC_17/28	Median	3100.00	3359.84	3340.80	3346.19	3300.02	3714.44	3568.69	3320.07	3363.29	3318.64
	Max	3321.67	3465.33	3422.81	3423.84	3300.02	3868.30	3690.78	3434.40	3436.87	3418.24
	Std	87.68	33.13	30.12	14.12	0.00	68.85	54.86	48.08	32.60	89.81
	Min	4591.75	6288.14	5572.02	6648.97	7001.12	9447.24	7363.53	4481.43	5471.30	3891.10
	Mean	5237.32	7887.23	6853.07	7269.34	8115.64	13591.33	8929.57	5025.78	7159.33	4447.44
CEC_17/29	Median	5259.43	7917.74	6745.60	7324.38	7955.99	13280.36	8823.02	5017.78	7143.42	4463.92
	Max	5945.51	9304.54	8091.26	7673.19	9897.92	18105.82	10486.00	5701.76	8218.33	5233.81
	Std	314.00	673.67	505.57	248.67	702.76	1731.80	654.17	304.74	509.78	246.88
	Min	5273.60	62774.26	5662.20	16891.33	40823905.93	70568462.27	10438810.60	5332.63	6649.44	5220.44
	Mean	5584.63	136331.10	9920.47	26961.79	64625903.41	220411229.62	19696989.78	5793.26	11182.19	5583.76
CEC_17/30	Median	5557.12	125436.83	8395.69	25904.40	64403338.35	191993438.42	19180090.35	5707.24	9618.69	5524.20
	Max	5998.00	291003.37	20481.54	42839.89	103310275.73	614413569.24	31214878.66	8781.00	21715.16	6321.60
	Std	187.47	53295.39	3704.77	6026.26	14556025.50	117525290.23	4647662.07	517.73	4049.77	246.34

**Table A8:** Statistical results of PCOA and other algorithms on the CEC2019, over 51 independent runs.

Function		PCOA	AVOA	PSO	DE	RW_GWO	WOA	HHO	LSHADE	GBO	EBOwithCMAR
	Min	1.00	1.00	15687.32	477575.81	1.00	8.79	1.00	1.00	1.00	1.00
	Mean	1.00	1.00	8542182.77	2006007.93	19824.68	3031265.52	1.00	1.00	1.00	18.48
CEC19_01	Median	1.00	1.00	2578552.14	1985994.72	897.58	2210414.02	1.00	1.00	1.00	1.41
	Max	1.00	1.00	90408077.34	3695753.18	208490.41	8949464.63	1.00	1.00	1.00	263.71
	Std	0.00	0.00	14190821.68	824405.80	46856.90	2921171.39	0.00	0.00	0.00	44.42
	Min	2.40	4.22	259.20	746.97	16.92	168.77	4.64	1.00	3.73	85.98
	Mean	3.06	4.54	2041.36	1170.76	239.10	7125.43	4.97	1.00	4.26	301.17
CEC19_02	Median	3.10	4.31	628.19	1165.37	231.73	6892.84	5.00	1.00	4.26	302.64
	Max	3.44	5.00	10311.45	1518.66	632.95	13211.22	5.00	1.00	4.52	564.38
	Std	0.16	0.35	2464.02	187.09	151.77	2648.86	0.09	0.00	0.10	106.03
	Min	1.00	1.00	1.41	1.60	1.00	1.41	1.00	1.00	1.00	1.00
	Mean	1.04	1.38	4.87	2.36	1.64	1.74	2.15	1.02	1.40	1.00
CEC19_03	Median	1.00	1.41	4.61	2.40	1.41	1.41	1.69	1.00	1.41	1.00
	Max	1.41	1.41	9.71	2.87	6.71	6.71	4.38	1.41	1.41	1.00
	Std	0.12	0.11	2.64	0.27	1.11	1.05	1.03	0.10	0.06	0.00
	Min	1.00	6.97	9.04	1.00	4.98	12.94	12.05	1.99	6.97	1.00
	Mean	1.00	22.34	24.98	1.06	14.48	42.13	45.21	3.52	23.29	1.00
CEC19_04	Median	1.00	19.90	24.52	1.01	13.46	39.81	43.94	2.99	21.89	1.00
	Max	1.00	46.77	44.58	2.03	27.63	73.63	80.82	5.97	45.41	1.00
	Std	0.00	9.30	8.72	0.20	4.89	14.34	13.45	1.10	9.01	0.00
	Min	1.00	1.08	1.03	1.03	1.08	1.17	1.59	1.00	1.03	1.00
	Mean	1.00	1.40	8.42	1.08	1.43	1.90	1.91	1.00	1.14	1.00
CEC19_05	Median	1.00	1.32	9.84	1.07	1.41	1.81	1.90	1.00	1.12	1.00
	Max	1.00	2.56	28.14	1.13	1.84	3.25	2.33	1.02	1.48	1.00
	Std	0.00	0.29	7.65	0.02	0.22	0.44	0.17	0.01	0.09	0.00
	Min	1.00	3.18	1.00	1.00	1.10	3.28	4.86	1.00	1.00	1.00
	Mean	1.00	5.93	4.65	1.07	1.59	7.92	8.06	1.00	3.74	1.00
CEC19_06	Median	1.00	5.99	4.67	1.01	1.25	7.74	7.92	1.00	3.55	1.00
	Max	1.00	9.32	9.03	1.48	4.31	11.76	11.26	1.00	8.08	1.00
	Std	0.00	1.41	2.08	0.13	0.78	1.54	1.52	0.00	1.85	0.00
	Min	1.12	245.49	238.45	4.12	105.53	482.69	369.43	1.19	300.26	1.06
	Mean	40.69	723.22	750.82	19.91	578.50	1059.58	983.63	33.65	797.86	46.17
CEC19_07	Median	4.60	727.08	718.49	17.71	565.65	1086.83	969.22	4.79	773.41	7.89
	Max	369.85	1281.30	1443.48	63.32	1137.52	1595.21	1564.28	238.52	1468.09	241.42
	Std	78.10	264.11	264.11	12.05	239.93	266.34	265.48	55.15	288.32	63.88
	Min	1.04	2.77	2.24	1.56	1.64	2.87	3.76	1.58	2.50	1.06
	Mean	1.39	3.76	3.59	2.21	3.00	4.19	4.40	2.20	3.59	1.21
CEC19_08	Median	1.26	3.76	3.60	2.20	3.05	4.17	4.43	2.22	3.61	1.21
	Max	2.62	4.51	4.52	2.80	4.04	4.98	4.98	2.65	4.55	1.83
	Std	0.37	0.37	0.54	0.28	0.61	0.35	0.30	0.24	0.43	0.15
	Min	1.01	1.09	1.04	1.07	1.04	1.07	1.10	1.02	1.04	1.00
	Mean	1.02	1.26	1.23	1.10	1.07	1.38	1.41	1.05	1.16	1.02
CEC19_09	Median	1.02	1.23	1.17	1.10	1.07	1.37	1.38	1.05	1.15	1.02
	Max	1.03	1.51	1.55	1.13	1.19	1.88	1.78	1.07	1.34	1.04
	Std	0.00	0.11	0.14	0.02	0.03	0.21	0.19	0.01	0.07	0.01
	Min	1.00	1.00	21.00	8.96	1.03	6.45	20.92	1.02	1.00	1.00
	Mean	12.62	20.63	21.02	20.16	19.71	20.76	21.01	9.06	19.13	1.19
CEC19_10	Median	15.04	21.00	21.01	21.04	21.25	21.00	21.00	3.02	21.00	1.00
	Max	21.00	21.25	21.14	21.07	21.37	21.34	21.10	21.00	21.22	3.01
	Std	8.27	2.80	0.04	2.73	5.34	2.05	0.03	9.15	5.86	0.54

**Table A9:** Statistical results of PCOA and other algorithms on the CEC2006, over 51 independent runs.

Function		PCOA	AVOA	PSO	DE	RW_GWO	WOA	HHO	LSHADE	GBO	EBOwithCMAR
	Function	PCOA	AVOA	PSO	DE	RW_GWO	WOA	HHO	L_SHADE	GBO	EBO_BIN
	Min	0.01	0.01	0.01	0.01	0.01	0.01	0.01	0.01	0.01	0.01
	Mean	0.01	0.01	0.01	0.01	0.01	0.01	0.01	0.01	0.01	0.01
CEC06_1	Median	0.01	0.01	0.01	0.01	0.01	0.01	0.01	0.01	0.01	0.01
	Max	0.01	0.02	0.02	0.01	0.01	0.02	0.02	0.01	0.01	0.01
	Std	0.00	0.00	0.00	0.00	0.00	0.00	0.00	0.00	0.00	0.00
	Min	0.00	0.00	0.00	0.00	0.00	0.00	0.00	0.00	0.00	0.00
	Mean	0.00	0.00	0.00	0.00	0.00	0.00	0.00	0.00	0.00	0.00
CEC06_2	Median	0.00	0.00	0.00	0.00	0.00	0.00	0.00	0.00	0.00	0.00
	Max	0.00	0.00	0.00	0.00	0.00	0.00	0.00	0.00	0.00	0.00
	Std	0.00	0.00	0.00	0.00	0.00	0.00	0.00	0.00	0.00	0.00
	Min	2522.48	2749.58	2749.58	2749.58	2751.00	2749.58	2749.63	2749.58	2749.58	2749.58
	Mean	2712.28	2750.28	2749.58	2749.58	2759.26	2886.49	2759.82	2749.58	2749.58	2749.58
CEC06_3	Median	2750.11	2749.83	2749.58	2749.58	2759.27	2776.88	2756.18	2749.58	2749.58	2749.58
	Max	2753.66	2754.02	2749.58	2749.58	2770.05	4205.24	2808.03	2749.58	2749.58	2749.58
	Std	64.90	0.99	0.00	0.00	4.29	284.02	11.74	0.00	0.00	0.00
	Min	464.66	464.80	464.68	466.05	464.92	478.80	468.23	464.65	464.68	464.65
	Mean	464.69	469.10	464.87	471.86	467.41	552.26	479.41	464.66	465.03	464.66
CEC06_4	Median	464.68	467.07	464.84	471.34	466.82	550.98	476.34	464.66	464.91	464.66
	Max	464.79	498.31	465.31	479.73	474.27	650.83	528.17	464.69	467.26	464.69
	Std	0.03	5.45	0.14	3.05	2.30	44.24	10.29	0.01	0.44	0.01
	Min	384.78	384.88	384.80	385.22	385.06	390.25	386.55	384.78	384.78	384.78
	Mean	384.82	385.72	385.00	386.09	386.34	471.09	397.62	384.79	384.99	384.78
CEC06_5	Median	384.81	385.59	384.94	386.05	386.40	465.47	396.08	384.79	384.91	384.78
	Max	384.93	387.54	385.82	387.54	388.30	624.02	418.70	384.81	385.44	384.81
	Std	0.04	0.58	0.20	0.48	0.73	60.32	7.57	0.01	0.18	0.01
	Min	455.35	455.50	455.37	457.97	455.80	461.81	462.46	455.35	455.36	455.35
	Mean	455.37	459.94	455.55	460.96	457.79	552.40	479.45	455.37	455.64	455.35
CEC06_6	Median	455.37	457.95	455.55	460.81	457.55	545.45	475.34	455.37	455.51	455.35
	Max	455.43	510.22	455.88	467.15	461.16	682.91	519.71	455.40	457.93	455.36
	Std	0.02	8.38	0.12	1.92	1.18	49.88	13.87	0.01	0.40	0.00

**Table A10:** Statistical results of PCOA and other algorithms on the CEC2011, over 25 independent runs.

Function		PCOA	AVOA	PSO	DE	RW_GWO	WOA	HHO	LSHADE	GBO	EBOwithCMAR
	Min	0.00	0.00	6.53	0.00	0.02	11.49	2.06	0.00	0.00	0.00
	Mean	1.41	15.73	16.48	4.60	13.46	19.05	19.41	0.02	14.66	0.00
CEC11_01	Median	0.01	16.66	14.93	3.80	12.32	19.85	21.42	0.01	14.91	0.00
	Max	9.79	22.75	24.14	12.84	21.97	24.03	25.10	0.09	24.75	0.00
	Std	3.00	5.15	5.28	4.09	5.72	3.77	4.89	0.03	6.71	0.00
	Min	−18.42	−18.42	−17.52	−13.65	−17.40	−16.31	−12.27	−15.25	−18.42	−12.43
	Mean	−16.66	−15.89	−13.90	−11.86	−14.58	−19.28	−14.95	−13.72	−16.70	−18.98
CEC11_02	Median	−17.11	−16.52	−15.33	−11.76	−15.36	−19.52	−15.17	−13.74	−16.52	−17.55
	Max	−18.86	−17.06	−11.11	−10.46	−10.27	−12.11	−1.73	−11.22	−15.34	−16.46
	Std	1.77	2.37	4.34	0.69	1.83	3.92	2.82	1.11	0.81	3.41
	Min	13.77	13.77	13.77	13.77	22.00	13.77	13.77	13.77	13.77	13.77
	Mean	13.77	13.93	15.74	19.04	22.00	13.86	14.06	14.08	13.96	14.56
CEC11_03	Median	13.77	13.77	14.33	20.82	22.00	13.77	13.98	14.33	13.85	14.33
	Max	13.77	14.33	20.96	21.54	22.00	14.33	14.33	14.33	14.33	20.96
	Std	0.00	0.26	3.00	2.92	0.00	0.21	0.27	0.28	0.24	1.39
	Min	−19.17	−19.17	−11.07	−11.84	−19.03	−19.16	−16.33	−19.16	−12.71	−139.58
	Mean	−15.72	−12.77	68.96	639993.58	−13.22	−19.58	−17.44	−19.15	599992.22	119927.45
CEC11_04	Median	−16.50	−13.01	28.67	999999.00	−13.01	−10.34	−16.84	−19.15	999999.00	−17.89
	Max	−11.27	−15.09	259.18	999999.00	−19.51	−12.42	−12.37	−19.09	999999.00	999999.00
	Std	2.91	3.64	89.24	489905.33	2.39	3.94	3.20	0.02	500008.48	331689.44
	Min	0.50	0.50	0.71	1.14	0.50	1.29	1.51	1.02	0.86	0.50
	Mean	0.50	0.92	0.94	1.45	1.12	1.84	1.79	1.24	1.12	0.61
CEC11_05	Median	0.50	0.96	0.98	1.48	0.95	1.84	1.76	1.26	1.11	0.60
	Max	0.50	1.76	1.23	1.62	2.24	2.11	2.09	1.41	1.48	0.94
	Std	0.00	0.42	0.15	0.11	0.45	0.19	0.16	0.10	0.17	0.10
	Min	1552.55	276.00	141739.85	70489.55	276.00	85737.56	636544.57	1445.63	1650.06	951.89
	Mean	2587.62	276.00	497339.93	77454.95	276.00	329594.72	866109.74	2320.93	27312.16	2746.01
CEC11_06	Median	2556.01	276.00	490832.51	78416.59	276.00	277460.83	829889.65	2172.22	6255.91	2486.81
	Max	4251.39	276.00	937154.93	86909.05	276.00	988037.86	1412178.14	3668.35	244113.32	6191.53
	Std	678.35	0.00	191825.36	4452.09	0.00	187525.11	172888.68	611.83	53248.10	1269.08
	Min	−11.74	50839.74	73657.95	2671395.85	179725.51	1079038.97	844051.65	51421.34	51516.81	49795.05
	Mean	−11.49	51931.42	977042.57	8108830.36	1226566.54	1283885.59	1077297.57	52340.32	52332.50	54633.15
CEC11_07	Median	−11.53	51966.51	864259.83	7906327.72	1166252.02	1289379.36	1075151.78	52431.04	52272.64	51036.64
	Max	−10.83	52886.07	2162549.98	17822571.76	2791576.91	1517540.11	1307857.18	53085.25	53129.64	140531.31
	Std	0.20	584.81	567785.02	3554121.32	696306.91	127610.82	107536.82	443.53	416.53	17905.28
	Min	50901.31	−1.00	1085435.80	2167360.78	1081583.43	4754050.00	5055128.46	1070988.13	1066501.11	1078713.57
	Mean	52052.82	−1.00	1150668.14	2424708.11	1164355.05	5734290.01	5636371.99	1073658.22	1089573.42	1091056.62
CEC11_08	Median	52011.79	−1.00	1125337.72	2409397.75	1145458.89	5705051.15	5732391.42	1073465.79	1076630.86	1089220.71
	Max	53009.27	−1.00	1432486.28	2592310.04	1305362.44	6836173.02	6136753.67	1077117.75	1170827.71	1116992.41
	Std	505.97	0.00	75513.32	108410.33	61487.58	517550.00	334879.67	1430.31	26038.43	8036.46
	Min	1074177.62	15459.24	15447.53	15451.83	15444.85	15482.09	15448.95	15444.19	15451.72	15447.68
	Mean	1077796.86	15509.64	15472.60	15466.50	15465.39	15542.90	15507.90	15444.19	15513.24	23783.56
CEC11_09	Median	1077450.40	15506.46	15467.74	15465.18	15464.02	15539.42	15502.58	15444.19	15489.84	15488.33
	Max	1084215.41	15619.66	15514.91	15497.40	15517.96	15689.94	15713.47	15444.19	15603.33	82872.58
	Std	2102.14	35.52	20.72	11.26	17.25	49.60	56.54	0.00	48.65	16470.80
	Min	15444.20	18751.11	18955.07	18864.00	18782.74	19045.79	18924.30	18018.81	18644.30	18117.65
	Mean	15444.40	19132.46	19236.57	19246.16	19162.64	19320.68	19331.89	18118.79	19010.07	18304.53
CEC11_10	Median	15444.33	19148.63	19276.62	19229.57	19144.88	19328.97	19365.14	18113.04	19005.19	18288.00
	Max	15445.15	19417.13	19573.09	19571.02	19528.45	19535.33	19681.85	18253.92	19360.71	18639.64
	Std	0.22	141.26	170.96	206.68	214.78	138.79	187.60	56.40	162.56	134.20
	Min	18028.35	32878.03	32902.41	32820.39	32886.66	33017.61	33011.09	32740.27	32748.88	33221.98
	Mean	18080.89	33179.87	33065.87	32908.11	33028.46	68765.72	79767.09	32740.79	32976.34	231364.35
CEC11_11	Median	18082.85	33180.67	33058.16	32896.03	33033.86	33330.44	33276.02	32740.56	32976.63	157042.72
	Max	18089.22	33444.90	33281.82	33017.64	33288.78	378760.69	351923.61	32743.42	33343.51	834765.88
	Std	11.04	131.17	94.80	44.91	93.90	81088.81	86648.34	0.69	118.44	235751.07
	Min	32747.13	128436.81	131901.91	132868.82	130831.47	136215.66	131465.71	124321.46	128658.44	123741.57
	Mean	32831.27	140780.47	138260.66	137048.59	138449.56	147430.49	147439.78	126600.93	142175.72	125055.74
CEC11_12	Median	32835.84	140744.76	137783.90	136949.60	137405.52	148383.66	147541.42	126430.83	143535.65	124859.69
	Max	32913.80	163201.86	146011.73	142049.26	152004.76	156292.50	160652.12	128586.34	153778.42	127272.45
	Std	39.71	8359.81	4299.43	2319.69	5202.79	5251.41	5928.64	1066.73	6792.22	859.40
	Min	122505.31	1930428.28	1945623.48	1944161.27	1945720.36	7726884358.46	2005470362.16	1861887.69	1927117.66	1855197.95
	Mean	123476.78	2561734.09	2494342.73	2186020.42	2503682.90	11903068077.39	3941812140.22	1885476.33	2395618.07	1971252.82
CEC11_13	Median	123485.62	2284701.14	2427551.13	2153785.96	2200330.35	11447819359.27	3797990141.83	1886486.24	2353480.53	1939378.23
	Max	124773.04	4399930.03	3962075.04	2521246.03	3890218.00	17528363172.57	6381060003.82	1913332.70	4529421.02	2283288.76
	Std	679.38	675718.43	538192.41	169472.29	572426.41	2835687898.14	1140520813.39	12151.17	583799.27	103990.59
	Min	1863920.05	941849.49	954638.61	1495756.99	951416.11	1126296.44	1143797.77	931788.50	940925.15	932554.77
	Mean	1903734.28	1274993.49	978836.56	2264687.02	969450.84	3778221.29	2518310.61	937371.26	1184095.29	936770.93
CEC11_14	Median	1902236.61	955856.60	974694.73	2228997.20	968567.12	2657983.25	2600770.02	937265.55	955554.68	936959.84
	Max	1929218.62	3168699.21	1113024.24	2956030.55	992366.83	14272534.86	3905498.51	941670.57	3140878.67	940695.62
	Std	15110.54	609751.60	29245.71	391035.75	9412.84	3045108.32	698322.05	2701.65	516519.40	2119.25
	Min	924988.38	1327658.14	1032135.13	2056007.54	1069126.57	1724267.96	1905637.22	937089.50	998182.72	937455.09
	Mean	926039.02	2046687.30	1258767.56	2828111.86	1309575.11	6264590.00	3297726.23	941015.11	1344472.06	942472.21
CEC11_15	Median	926037.70	1786470.00	1249938.18	2844296.39	1303127.52	4314947.66	2886428.18	940552.14	1279200.17	942014.10
	Max	927502.52	3751699.50	1487934.82	3416317.88	1663842.81	23442234.17	7092458.85	948157.10	2801185.32	953980.50
	Std	715.35	676209.07	134492.73	327779.35	140301.90	5275163.04	1255903.48	2589.49	421179.08	3497.43
	Min	928359.37	945322.70	957289.68	1533893.63	966100.40	1123529.06	1280726.03	933470.94	942804.32	932637.67
	Mean	933631.94	1316757.53	972938.11	2195326.57	987737.54	4475588.85	2367518.16	937300.70	1106441.65	936145.02
CEC11_16	Median	933990.16	1068889.56	972340.12	2109765.22	977394.59	2643035.90	1867048.70	937066.15	957536.60	936002.39
	Max	938931.36	2556828.08	987884.90	3014580.73	1097222.42	16686744.65	5039477.25	942275.84	2437343.25	939918.90
	Std	2536.51	506756.23	7858.08	398425.84	30763.56	4492618.92	1026801.36	2477.72	391839.26	1578.15
	Min	924739.22	19919191.15	957289.68	1533893.63	957360.13	1123529.06	1280726.03	933470.94	942804.32	932637.67
	Mean	926182.48	26324193.34	972938.11	2195326.57	973923.72	4475588.85	2367518.16	937300.70	1106441.65	936145.02
CEC11_17	Median	926235.72	26362916.56	972340.12	2109765.22	972845.75	2643035.90	1867048.70	937066.15	957536.60	936002.39
	Max	928083.87	31358238.73	987884.90	3014580.73	990713.06	16686744.65	5039477.25	942275.84	2437343.25	939918.90
	Std	926.03	2945458.10	7858.08	398425.84	7506.78	4492618.92				
